# Cytochrome P450 enzymes in the phenylpropanoid pathway: CYP73 and CYP98 candidate genes in *Chara braunii*, *Anthoceros agrestis*, *Marchantia polymorpha* and *Physcomitrium patens*

**DOI:** 10.1111/tpj.71152

**Published:** 2026-10-07

**Authors:** Christoph E. G. Aggarwal‐Kentrath, Maike Petersen

**Affiliations:** ^1^ Philipps‐Universität Marburg, Institut für Pharmazeutische Biologie und Biotechnologie Robert‐Koch‐Straße 4 Marburg 35037 Germany

**Keywords:** cytochrome P450, CYP73, cinnamate 4‐hydroxylase (C4H), CYP98, 4‐coumaroyl ester/amide 3‐hydroxylase, phenylpropanoid metabolism, bryophytes, streptophyte algae

## Abstract

CYP73 and CYP98 are two cytochrome P450 monooxygenase families present in bryophytes and tracheophytes, and they play key roles in the formation of phenolic compounds containing a caffeic acid moiety and more complex phenolic compounds. CYP73 encodes the second enzyme in the core phenylpropanoid pathway and is the first enzyme family in this pathway so far exclusively found in land plants and not in any water‐living sister clades, like the streptophyte algae. That makes CYP73 a prime candidate for investigations into necessary adaptations during terrestrialization. This study aims to support our knowledge on the evolution of this pathway by characterizing the enzymes translated from (putative) CYP73 genes from the bryophytes *Anthoceros agrestis*, *Marchantia polymorpha*, and *Physcomitrium patens* and CYP98 genes from *Marchantia polymorpha* and *Physcomitrium patens*. Furthermore, this insight into the function of bryophyte CYP73 informed a more specific search for potential CYP73 candidates in the streptophyte alga *Chara braunii*. The possibility that land plant CYP73 activity originated in streptophyte algae would be greatly supported if rudimentary CYP73 activity could be found in native *Chara* candidate genes or induced by slight mutations in inactive *Chara* enzymes. However, no such mutation led to product formation by enzymes encoded by *Chara braunii* candidate genes. The bryophyte CYP98 enzymes prefer 4‐coumaroyl amides as substrates, though 4‐coumaroyl esters are generally the common substrates of tracheophyte CYP98 enzymes. This shows a major difference in functionality. Thus, this study provides a comparative characterization of CYP genes from model bryophytes that are canonically associated with phenylpropanoid metabolism.

## Significance Statement

CYP73 and CYP98 candidate genes were studied in the genomes of the bryophytes *Anthoceros agrestis*, *Marchantia polymorpha*, and *Physcomitrium patens*, and the encoded proteins expressed in yeast for the determination of their substrate acceptance and affinities. While all active CYP73 proteins displayed true C4H activity, CYP98 did not catalyze the *meta*‐hydroxylation of the generally assumed substrates (4‐coumaroyl esters such as 4‐coumaroyl‐5’‐*O*‐shikimate, 4‐coumaroyl‐5’‐*O*‐quinate, 4‐coumaroyl‐4′‐hydroxyphenyllactate, 4‐coumaroyl‐3′,4′‐dihydroxyphenyllactate), but preferred *N*‐(4‐coumaroyl)‐anthranilate and *N‐*(4‐coumaroyl)‐3′‐hydroxyanthranilate.

## INTRODUCTION

The phenylpropanoid pathway provides precursors for a multitude of compounds, some of which are regarded as essential for the successful colonization of the land by plants (Cheynier et al., 2013; Rieseberg et al., 2023). Flavonoids and anthocyanins, as well as hydroxycinnamate esters, provide protection against increased UV‐irradiation (Cheynier et al., 2013; Stelzner et al., 2019). The emergence of water‐conducting tissues and the possibility to rise against gravity was facilitated by the monolignol polymer lignin (Boerjan et al., 2003). Cutin and suberin confer resistance against excessive water loss (Knosp et al., 2024). Furthermore, a number of phenolic compounds have antibacterial and antifungal properties and deter pathogens and herbivores with their taste (Singh et al., 2021).

Pathways leading to these compounds canonically involve cytochrome P450 monooxygenases. However, homologous genes for the first and most crucial one of those CYP enzymes were, to date, only found in embryophytes. Still, UV‐absorbing pigments have already been observed in predecessors of land plants, such as the Zygnematophyceae, raising the question of their biosynthesis. Algae such as *Ancylonema nordenskiöldii* or *A. alaskanum* (syn. *Mesotaenium berggrenii* var. *alaskanum*; Zygnematophyceae) live on the surface of glaciers in an intermediate environment between water and land and are exposed to high UV‐irradiation. A phenolic purpurogallin derivative, accumulated in vacuoles, was found to be responsible for the red‐brownish pigmentation observed under high light intensities (Bowles et al., 2024; Remias et al., 2012; Williamson et al., 2020). In the metagenome of *A. nordenskiöldii*, however, it was shown that extensive gene loss and contraction of gene families led to a reduction in the functions of the canonical phenylpropanoid metabolism (see below), generally associated with the biosynthesis of phenolic compounds (Bowles et al., 2024). A different “sunscreen strategy” was found in another species of the Zygnematophyceae, *Serrataenia testaceovaginata*, which produces an extracellular mucilage enriched with UV‐absorbing pigments under UV‐irradiation (Busch et al., 2024). After UV‐exposure, genes of the shikimate pathway leading to aromatic amino acids were upregulated, but genes encoding enzymes of the core phenylpropanoid and flavonoid pathways were either absent or not upregulated. Some genes similar to those encoding enzymes of phenolic metabolism in land plants (e.g., 4CL, CAD/CCR, *O*‐methyltransferases, glycosyltransferases) were detected, but their functionality was not proven.

Real lignin, composed of monolignol units, derivatives of the phenylpropanoid pathway, has been found in the secondary cell walls of a member of the Rhodophyta (*Calliarthron cheilosporioides*) (Martone et al., 2009). The transcriptomic analysis of a related species (*Calliarthron tuberculosum*) identified gene candidates for several enzymes involved in phenolic metabolism (4CL, CCR, CAD, CCoAOMT, CSE), while others seemed to be missing (PAL, C4H, HCT, C3H, COMT, F5H). Phylogenetic analyses, however, suggested that these gene candidates evolved independently from the respective land plant enzymes, strongly indicating a convergent evolution of the lignin biosynthetic pathway in red algae (Xue et al., 2022). Real lignin is still considered a unique trait for tracheophytes, and Renault et al. (2019) have suggested that lignin in *Calliarthon* could also be derived from driftwood, which has entered the ocean from the land and was possibly utilized by the red alga. Some bryophytes have already incorporated phenolics into their cell walls, long before being capable of the biosynthesis of real lignin, possibly achieving a similar purpose to a lesser extent (Renault et al., 2019). While the phenylpropanoid pathway may be the key to the successful colonization of land, the earlier iteration of phenolic metabolism in the land plant sister lineages and in bryophytes gives some insight into its evolution.

In embryophytes, the core phenylpropanoid pathway encompasses two cytosolic enzymes, phenylalanine ammonia‐lyase (PAL) and 4‐coumarate CoA‐ligase (4CL), as well as one ER‐associated CYP73 family cytochrome P450 monooxygenase, cinnamate 4‐hydroxylase (C4H, CYP73; E.C. 1.14.14.91) acting together with the electron transfer protein NADPH:cytochrome P450 reductase (CPR; E.C. 1.6.2.4), a flavoprotein containing FAD and FMN as prosthetic groups. CYP73 is a typical member of the class II ER‐bound monooxygenases acting in concert with CPR (Werck‐Reichhart & Feyereisen, 2000). As a cytochrome P450 monooxygenase, C4H activity needs O_2_ and NADPH, the electrons of which are transferred by CPR to the cytochrome. One atom of O_2_ is incorporated into the product (*t*‐cinnamate → 4‐coumarate) while the other atom of O_2_ is released as water (Figure 1, Figure S1).

**Figure 1:**
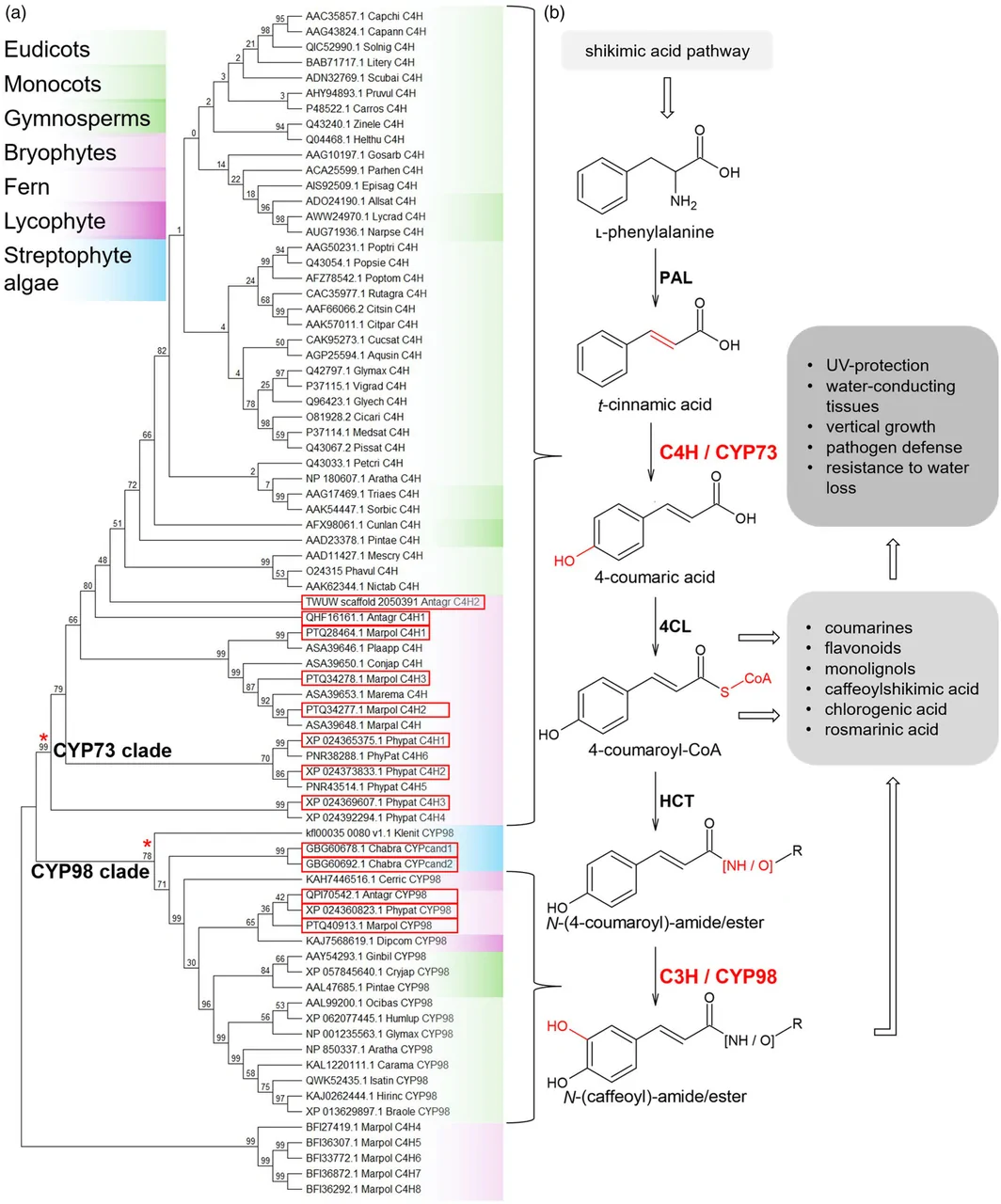
Overview of CYP73 and CYP98 phylogeny and their functions in the phenylpropanid pathway. (a) Phylogenetic tree depicting the evolutionary relationship between CYP73 and CYP98 amino acid sequences from different clades of streptophytes. Enzymes under investigation in this study are shown in red boxes. The color gradient of the tree backbone is shown in the left corner. For detailed species abbreviations and accession numbers, see Table S6. (b) Core phenylpropanoid pathway starting with l‐phenylalanine leading to 4‐coumaroyl‐CoA followed by the CYP98‐catalyzed hydroxylation of a 4‐coumaroyl ester/amide. PAL, phenylalanine ammonia‐lyase; C4H, cinnamic acid 4‐hydroxylase; 4CL, 4‐coumarate CoA‐ligase; HCT, hydroxycinnamoyltransferase; C3H, 4‐coumaroyl ester/amide 3‐hydroxylase. Changes introduced by the respective enzymes are marked in red.

The first C4H was already biochemically characterized in 1967 from pea seedlings (Russell, 1971; Russell & Conn, 1967). Generally, *t*‐cinnamate is assumed to be the preferred substrate of CYP73. A crystal structure of an *N*‐terminally truncated CYP73 (thus lacking the membrane anchor) from *Sorghum bicolor* (CYP73A33) in its detergent‐solubilized form was published in 2020 (pdb‐code: 6VBY) (Zhang et al., 2020). The authors describe a very similar overall structure compared to mammalian cytochrome P450s, with 16 α‐helices and strongly conserved heme‐binding sites and a heme‐binding loop. Heme is deeply buried in the overall structure of the protein. The amino acid residues involved in the binding of the substrate *t*‐cinnamate and the product 4‐coumarate, as well as oxygen and proton transfer, have been identified by docking studies and are marked in Figure S2 in the alignment of this crystallized CYP73 together with the CYP73 candidates described in this paper.

In general, CYP enzymes possess several conserved regions, including a proline‐rich *N*‐terminal hinge and the characteristic AxxxTT motif within the I‐Helix, which is critical for substrate binding and proton delivery and is typically accompanied by a nearby arginine residue, which was shown to be essential for C4H activity (Knosp et al., 2024). Other conserved regions are the FxPxRF motif in the F‐G loop region, which is important for substrate access, as well as two essential heme‐binding domains, ExxR and PFGxGRRxCPG, at the *C*‐terminal end of the protein. While the *N*‐terminal membrane anchor is one of the more variable regions, a high density of hydrophobic amino acids is generally observed, consistent with transmembrane location (Chen et al., 2020; Schoch et al., 2003; Xia et al., 2017).

The CYP73 family of cytochromes P450 has undergone major duplication and subfunctionalization events in land plants. Genes encoding CYP73 are reported to be absent from algae, although cytochrome P450 genes in general could be retrieved from algal genomes (de Vries et al., 2021; Renault, de Marothy, et al., 2017). The absence of CYP73 in algae but their functional presence in land plants was recently further investigated by Knosp et al. (2024) who pointed out an arginine residue in the active site to be essential for C4H activity with *t*‐cinnamate. They further showed that important structural features like the formation of a functional cuticle are based on C4H activity. Even though the formation of 4‐coumarate and its derivatives has been shown to be present in algae, it remains unclear how this is done, due to the absence of identifiable CYP73 candidates (Dadras et al., 2025; Goiris et al., 2014) and alternative pathways towards 4‐coumarate have to be taken into consideration. Recently, PAL as well as 4CL genes were identified in *Chara braunii*, and the encoded enzymes were proven to be active. Thus, the first and third enzymes of the core phenylpropanoid pathway are present and active in charophyte algae, even if their catalytic efficiency is low (Schwarze & Petersen, 2024). It is unlikely that 4‐coumarate is formed from l‐tyrosine by tyrosine‐ammonia lyase (TAL) activity since an ammonia‐lyase preferring l‐tyrosine could not be identified in *Chara braunii* or in bryophytes, and all respective enzymes definitively preferred l‐phenylalanine as substrate (Schwarze & Petersen, 2024) and thus form *t*‐cinnamate. Moreover, to date, enzymes with phenylalanine as well as tyrosine ammonia‐lyase activities (PTALs) have only been identified in monocotyledonous plants (Barros & Dixon, 2020).

The number of CYP73 genes in each species is variable. According to Ehlting et al. (2006) and Renault, Alber, et al. (2017), a gene duplication event occurred in the ancestor of seed plants which is retained in most angiosperms. One of these CYP73 genes has a simple *N*‐terminal transmembrane anchor and is assumed to be related to lignin biosynthesis, while the second has an elongated *N*‐terminus and variable functions. Nevertheless, the seed plant model organism *Arabidopsis thaliana* has only one CYP73 copy (Bak et al., 2011). In bryophytes and gymnosperms, multiple duplications are observable, which led to a multitude of gene copies (Ehlting et al., 2006).

The first C4H characterized from bryophytes in detail was obtained from the hornwort *Anthoceros agrestis* (Petersen, 2003). This first characterization in microsomes isolated from plant cell cultures was followed by gene identification and heterologous expression of AaC4H1 (CYP73A260) in *Physcomitrium patens* (Wohl & Petersen, 2020). Two isoforms of CYP73 from *Marchantia paleacea* and one from *Plagiochasma appendiculatum* (both liverworts) were expressed in the yeast strain WAT11 and analyzed by Liu et al. (2017). Recently, selected sequences from *Marchantia polymorpha*, *Physcomitrium patens*, and *Phaeoceros carolinianus* were expressed in yeast and shown to catalyze the conversion of *t*‐cinnamate to 4‐coumarate (Knosp et al., 2024). Knock‐out lines of *Physcomitrium patens* impairing the formation of active CYP73 resulted in strong phenotypes with high numbers of protonemata, slow growth of gametophores, and fused phyllids. Moreover, the content of 4‐coumaric and caffeic acids and their esters was reduced significantly, and the functionality of the plant's cuticle was impaired (Knosp et al., 2024). In *Marchantia polymorpha*, CRISPR‐mutants with non‐functional MpC4HA1 resulted in reduced thallus growth and reduced number of gemmae cups despite the presence of other functional CYP73 orthologs (Knosp et al., 2024).

A second cytochrome P450, belonging to the CYP98 family, is active in hydroxylating the 4‐coumaroyl moiety in phenolic esters, such as 4‐coumaroyl‐5’‐*O*‐quinate, 4‐coumaroyl‐5’‐*O*‐shikimate or 4‐coumaroyl‐3′,4′‐dihydroxyphenyllactate, to the caffeoyl substitution pattern, namely chlorogenate (caffeoyl‐5‐*O*‐quinate), caffeoyl‐5‐*O*‐shikimate and rosmarinate (caffeoyl‐3,4‐dihydroxyphenyllactate) (Eberle et al., 2009; Ehlting et al., 2006; Schoch et al., 2006) (Figure S3). As described for CYP73 above, CYP98 is dependent on O_2_ and electron transfer from NADPH by CPR and also shares similar conserved motifs to utilize the cofactors (Figure S4). Caffeate or caffeoyl‐CoA can then be released from the ester, in angiosperms especially from caffeoyl‐5‐*O*‐shikimate, by caffeoylshikimate esterase (CSE) (Vanholme et al., 2013) or by the reverse reaction of hydroxycinnamoyltransferases (Hoffmann et al., 2003), to enter into further metabolic pathways, such as the formation of monolignols for lignin biosynthesis or the delivery of phenylpropanoid moieties in cutin (Philippe et al., 2020; Renault, Alber, et al., 2017). Other caffeoyl esters, such as chlorogenate or rosmarinate, can be considered as the final stage of metabolic pathways. A functional CSE has recently been described to be active in the hornwort *Anthoceros agrestis* as the first bryophyte CSE. The enzyme efficiently releases caffeic acid from caffeoylshikimate, but also from chlorogenate and *N*‐(caffeoyl)‐3′‐hydroxyanthranilate, in addition to having lipase activity (Marks & Petersen, 2026).

CYP98 is mostly found in land plants as suggested by de Vries et al. (2021), although the same group found an ortholog of CYP98 in *Klebsormidium nitens* (de Vries et al., 2017). Algae, however, also contain a number of cytochrome P450 sequences with unknown functions. In *Chara braunii*, seven cytochrome P450 sequences have been retrieved, none of them showing convincing similarities to the CYP98 family (de Vries et al., 2021). With the exception of angiosperms harboring multiple CYP98 copies, all greenland plant lineages contain only one copy of CYP98. It is theorized that the encoded enzyme plays a crucial role in the biosynthesis of all compounds with a further elaborated substitution pattern in the phenylalanine‐derived aromatic ring (Alber et al., 2019). In *Physcomitrium patens*, CYP98 is highly expressed in erect aerial organs. Deletion of the only CYP98 resulted in altered gametophore development with organ fusion and gametophore abortion, as well as disturbed cuticle formation. Furthermore, the metabolic profile changed towards more 4‐coumaroyl‐ and less caffeoyl‐containing compounds, especially peaking in higher levels of 4‐coumaroyl‐4’‐*O*‐threonate (Renault, Alber, et al., 2017). A disturbance in organ formation after CYP98 knockout in *P. patens* has also been observed by Xin et al. (2023). While gymnosperm CYP98s were shown to accept 4‐coumaroyl‐5’‐*O*‐shikimate as preferred substrate, the corresponding enzymes from *Physcomitrium patens* (moss), *Selaginella moellendorffii* (lycophyte) and *Pteris vittata* (fern) as well as *Anthoceros agrestis* (hornwort) acted poorly on 4‐coumaroyl‐5’‐*O*‐shikimate but preferred *N*‐(4‐coumaroyl)‐anthranilate as best substrate (Alber et al., 2019; Ernst et al., 2022). However, *N*‐(4‐coumaroyl)‐ or *N*‐(caffeoyl)‐anthranilate have not been detected in these organisms to date. Since CYP98 underwent gene duplication and neofunctionalization in angiosperms, CYP98 isoforms in angiosperms accept a wide range of 4‐coumaroyl esters and amides as substrates, e.g., 4‐coumaroyl‐4′‐hydroxyphenyllactate in rosmarinate biosynthesis (Eberle et al., 2009) or *N* ^1^,*N* ^5^,*N* ^10^‐tricoumaroylspermidines as substrates for 3‐hydroxylation in the formation of important pollen coat constituents (Liu et al., 2016). The first biochemically characterized CYP98, CYP98A3 from *Arabidopsis thaliana*, was found to be the “missing link” in phenylpropanoid metabolism, catalyzing the *meta*‐hydroxylation of 4‐coumaroyl‐5′‐*O*‐shikimate and ‐quinate to the corresponding caffeoyl derivatives (Schoch et al., 2001). The release of caffeate/caffeoyl‐CoA from the ester opens the pathway to monolignols with caffeoyl (C‐monolignol), coniferyl (G‐monolignol), and sinapyl (S‐monolignol) substitution patterns and thus to lignification.

Since the organisms we investigated, *Anthoceros agrestis*, *Physcomitrium patens*, and *Marchantia polymorpha*, as well as the charophyte alga *Chara braunii*, are not lignified, but at least the former three contain one CYP98 gene each, the function of CYP98 in these organisms has to be investigated. In a metabolic profiling study, caffeic and chlorogenic acids have been reported to be present in the gametophores of *Physcomitrium patens* (Erxleben et al., 2012). Downregulation of CYP98 in *Physcomitrium patens* showed perturbations in phenolic metabolism. The authors report a decrease in chlorogenate and 4‐coumaroyltartrate contents, as well as a decrease in certain flavonoids (Xin et al., 2023). *Physcomitrium patens* CYP98 has been previously related to the formation of caffeoyl‐4‐*O*‐threonate, which has been shown to be important for cuticle formation (Renault, Alber, et al., 2017). *Selaginella moellendorffii*, on the other hand, contains lignin and one member of CYP98, which, in this case, is not involved in lignification. Here, CYP788A1 is involved in 3‐ as well as 5‐hydoxylation of the aromatic ring (Weng et al., 2010).

In the hornwort *Anthoceros agrestis*, rosmarinic acid (RA), an ester of caffeic and 3,4‐dihydroxyphenyllactic acids, is accumulated (Takeda et al., 1990; Vogelsang et al., 2006). The only CYP98 identified and characterized from this species mainly hydroxylates *N*‐(4‐coumaroyl)‐anthranilate and *N*‐(4‐coumaroyl)‐3′‐hydroxyanthranilate and to a considerably lower extent 4‐coumaroyl‐5’‐*O*‐shikimate (Ernst et al., 2022). The precursor for RA was not hydroxylated, leaving the question of how RA is formed in this hornwort open. However, the occurrence of caffeic acid derivatives in bryophytes makes the participation of CYP98 in the formation of these compounds probable.

Here we report on a thorough search for CYP73‐ and CYP98‐encoding genes in the hornwort *Anthoceros agrestis*, the liverwort *Marchantia polymorpha* and the moss *Physcomitrium patens*, as well as putative candidates in the charophyte alga *Chara braunii* as extant model organisms related to those taxa putatively involved in early terrestrialization. Comparing the land plant CYPs with potential CYP candidates found in aquatic plants may give insight into their importance during terrestrialization. Selected candidate sequences were codon‐optimized for expression in *Saccharomyces cerevisiae* and biochemically characterized to gain more insight into their catalytic activities as well as the evolution of the phenylpropanoid pathway.

## RESULTS

### Identification and analysis of CYP73 sequences (cinnamate 4‐hydroxylase)

The Phytozome 13 database returned six CYP73 sequences for *Physcomitrium patens* and eight for *Marchantia polymorpha* (Table S1). Pairwise comparisons of the sequences within one species were performed. The six *Physcomitrium patens* sequences revealed pairwise identities/similarities ranging between 43.9%/54.1% (identity/similarity) and 76.7%/86.2% (Table S2). Phylogenetic analyses showed that the six sequences were split into three closely related groups of two sequences each, suggesting that gene duplication events may have occurred (Table S2). One sequence of each pair was chosen for further characterization as PpC4H1, PpC4H2, and PpC4H3 (Table S1). The same analysis for CYP73 from *Marchantia polymorpha* resulted in a higher level of dissimilarity (Table S3). Here, the identities/similarities of the eight amino acid sequences were between 24.0%/42.8% and 82.0%/91.3%. Due to the absence of some of the important conserved sequence motifs, e.g. AxxxTT, in five out of the eight sequences, only three (MpC4H1, MpC4H2, MpC4H3) were chosen for further characterization (Table S1).

For *Anthoceros agrestis*, only two sequences were retrieved in the 1kP database, one of which had previously been partially analyzed as AaC4H1 expressed in *Physcomitrium patens* (Wohl & Petersen, 2020). The two sequences displayed a sequence identity of 66.5% and a similarity of 75.1% to each other.

A search for putative CYP73 sequences in *Chara braunii* retrieved no convincing results. However, the most similar sequence identified was rna‐CBR_g12414 (GenBank GBG60678.1) and was included in our research as CbCYP_cand_1. The second closest match was rna‐CBR_g12428 (GenBank GBG60692.1) and was also included in this study as CbCYP_cand_2. The sequences were selected based on the identification of conserved motifs and the annotation of CbCYP_cand_2 as CYP98, even if it showed no clear characteristic that would make it more likely to belong to the CYP98 family rather than the CYP73 family.

All sequences were analyzed for their signal peptides and membrane anchors (DeepTMHMM) (Table S1) since eukaryotic CYP proteins generally are anchored in the membranes of the endoplasmic reticulum (“microsomal type”; Omura, 2010) in close proximity to a CPR. Membrane anchors were identified for all CYP73 proteins except CbCYP_cand_1, MpC4H3 and PpC4H3. Interestingly, MpC4H3 was characterized as active, while the other two enzymes without identifiable membrane anchors were inactive in our hands.

Additional information about the existence of transmembrane domains can be found in the 3D structure predicted with AlphaFold 3. Each folded protein structure, including the ones of CbCYP_cand_1 (Figure 2), MpC4H3 and PpC4H3, shows an *N*‐terminal structure, not unlike a corkscrew, that includes a long stretch of hydrophobic amino acids suitable for membrane anchoring. The angle at which this corkscrew‐like structure extends from the globular body of the protein varies between all CYP enzymes, and some enzymes show a second corkscrew within an even longer *N*‐terminal end, forming a hairpin‐like structure (Figure S5).

**Figure 2:**
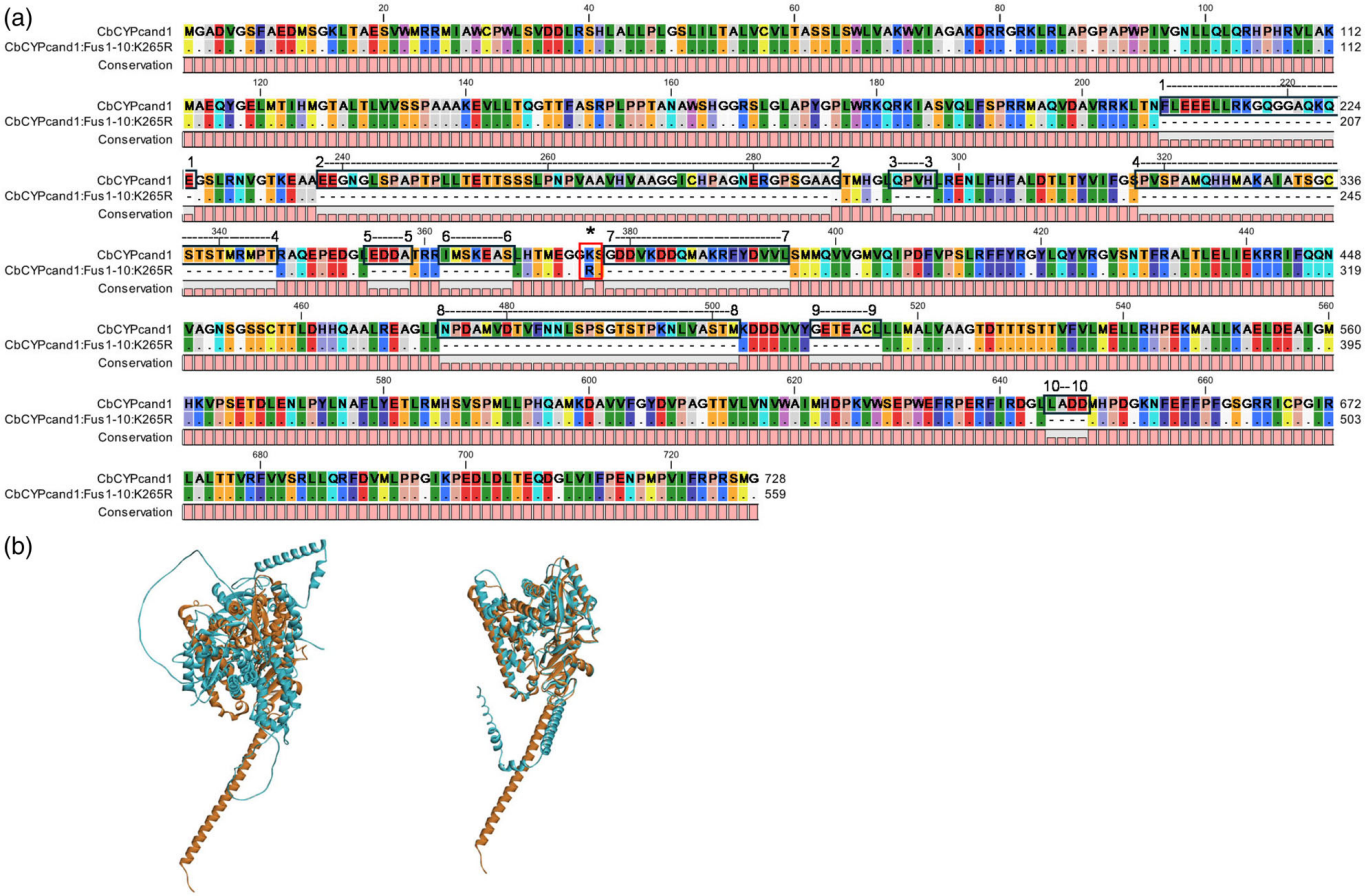
Adaptations made to CbCYPcand1 based on multiple sequence alignments and 3D structure comparison. (a) Alignment of CbCYP_cand_1 and CbCYP_cand_1:Fus1‐10:K265R in CLC Sequence Viewer 8. The ten black boxes mark the amino acid residues that did not align with other CYP73 amino acid sequences. The coding regions for these amino acids have been removed in CbCYP_cand_1:Fus1‐10:K265R. The red box with the asterisk above marks the l‐lysine residue that was mutated to l‐arginine in both CbCYP_cand_1:K376R and CbCYP_cand_1:Fus1‐10:K265R. (b) 3D models of proteins were predicted with AlphaFold 3 and aligned with Discovery Studio 2024 Client (BIOVIA). Left: alignment of CbCYP_cand_1 (blue) with PpC4H1 (orange), right: CbCYP_cand_1:Fus1‐10:K265R (blue) was aligned with PpC4H1 (orange).

### Comparison and alignment of CYP73 sequences

All amino acid sequences from the three bryophytes and *Chara braunii* were aligned. SbC4H1 from *Sorghum bicolor*, which had previously been crystallized and characterized (CYP73A33; GenBank AAK54447.1; pdb‐code: 6VBY) (Zhang et al., 2020), as well as Jerusalem artichoke CYP73 HtC4H1 (CYP73A1; GenBank Q04468.1) with identified substrate recognition sites (Schoch et al., 2003), were added to the alignment (Figure S2). Conserved amino acid residues for substrate recognition, heme‐binding, as well as conserved sequence motifs identified in *Sorghum bicolor* CYP73 were generally also found in the CYP73 genes of the investigated bryophytes but were largely missing in the *Chara* sequences. The AxxxTT motif of all bryophyte CYP73 enzymes investigated more closely was uniform (AAIETT) even within the variable amino acid residues. The highest deviation within the mosses was observed for PpC4H3, which – in our hands – did not display catalytic activity. Here, amino acid residues lining the substrate entrance channel and those involved in electron transfer showed the highest deviations, perhaps explaining the missing enzymatic activity.

The amino acid sequences of CbCYP_cand_1 and CbCYP_cand_2 were considerably longer than the bryophyte sequences, with 728 and 654 amino acids, respectively, and included interspersed stretches, especially in the first 2/3 of the protein, that also interrupted conserved sequence motifs. Especially an arginine residue, which was identified to be mandatory for C4H activity by Knosp et al. (2024), was lacking in CbCYP_cand_1 and 2, while it was found in every other CYP73 sequence of active enzymes. In addition, CbCYP_cand_1 and 2 lack the CYP73 consensus AAIETT motif in the I‐Helix. While CbCYP_cand_1 resembled the AGMDTT consensus sequence of CYP98 more closely, with one change in the variable region, CbCYP_cand_2 lacked this motif entirely. Lastly, the crucial first l‐Phe of the FxPxRF motif was substituted with l‐Leu in the CbCYP_cand_2 sequence.

Overall identities between the CYP73 sequences under investigation mostly ranged around or higher than 60% at the amino acid level. The highest differences were seen in the length and composition of the *N*‐terminus. Here, some bryophyte sequences are considerably longer than the *Sorghum* and *Helianthus* sequences. Exceptions were AaC4H1 and PpC4H2, where the *N*‐terminal sequences are visibly shorter than those of other bryophytes.

### Identification and analysis of CYP98 sequences (4‐coumaroyl ester/amide 3‐hydroxylase)

The same databases as for CYP73 were searched for CYP98 sequences using the already identified CYP98A147 (GenBank QPI70542.1) from *Anthoceros agrestis* (Ernst et al., 2022) as bait sequence. As expected, only one sequence with high similarity was returned for each of the bryophytic model organisms: Mapoly0037s0087 = MpCYP98 (GenBank PTQ40913.1) for *Marchantia polymorpha* and Pp3c22_19010 = PpCYP98 (GenBank XP_024360823.1) for *Physcomitrium patens*. No further CYP98 could be retrieved for *Anthoceros agrestis*. In *Chara braunii*, no fully convincing result could be retrieved, but the two most likely candidates for CYP73 (see above) were also included here. All bryophyte sequences carried membrane anchors as identified by DeepTMHMM (Table S4). Pairwise comparison of the amino acid sequences (except CbCYP_cand_1 and 2) showed overall identities of >60% and similarities of >75% (Table S5). This was also reflected in a sequence alignment (Figure S4).

When extending the search for CYP98 sequences to all available Charophyta genomes, one more partial sequence from *Mesotaenium kramstae* (Zygnematophyceae) showed high similarity (identity 36.5%, similarity 52.2%; Meskra_scaffold‐NBYP‐2004150) with the bait sequence. However, about 11 amino acids are missing at its *N*‐terminal end. Also, the AxxxTT motif was altered in two of the variable positions (Table S4).

### Comparison and sequence alignment for CYP98

All CYP98 sequences described in this paper were aligned together with *Ocimum basilicum* CYP98 (ObCYP98; GenBank AAL99200.1; Gang et al., 2002) for comparison with CYP98 from seed plants. The two candidate sequences from *Chara braunii* were also included. All sequences show the typical substrate recognition and heme‐binding sites, differing only in the AxxxTT motif when compared to CYP73. While in CYP73 the consensus sequence is AAIETT, the oxygen‐binding site is found to be AGMDTT in all CYP98 candidate genes investigated and AGTDTT for CbCYP_cand_1. The length of the embryophyte CYP98s was consistent between 506 and 529 amino acid residues (Figure S4).

### Expression analysis for CYP73 and CYP98

The expression data for verified and putative CYP73 and CYP98 were taken from PEATmoss (https://peatmoss.plantcode.cup.uni‐freiburg.de) for *Anthoceros agrestis* and *Physcomitrium patens* and MarpolBase (https://mbex.marchantia.info) for *Marchantia polymorpha*.

For *Anthoceros agrestis*, both C4H orthologs were expressed at high levels throughout all types of tissues. The only exception is the low expression of AaC4H1 in gametophytes. In *Marchantia polymorpha*, MpC4H1 was the isoform expressed at the highest level, especially in thalli, antheridiophores, archegoniophores, and archegonia. MpC4H2 and MpC4H3 only showed low expression in younger thalli and even less in all other tissue types. In *Physcomitrium patens*, PpC4H1 was the isoform expressed the most, with the highest levels in gametophores. PpC2H was actively expressed in germinating spores, protonemata, and protoplasts, whereas PpC4H3 was only weakly expressed (Figure S6).

CYP98 from *Anthoceros agrestis* was expressed at equally high levels throughout the organism, while MpCYP98 from *Marchantia polymorpha* has its highest expression in archegoniophores and antheridiophores, followed by thallus, archegonia, and sporophyte. PpCYP98 was barely expressed in dry spores of *Physcomitrium patens*, but the expression increased in germinating spores. A low expression can be seen in protonemata and protoplasts, while the highest level of expression is recorded for adult gametophores (Figure S6).

### Phylogenetic analysis

A phylogenetic tree (Figure 1) was calculated using the Maximum Likelihood algorithm (1000 bootstraps), including CYP73 and CYP98 amino acid sequences of various seed plant groups as well as the sequences of verified and putative CYP73 and CYP98 proteins from bryophytes and the two CYP candidates from *Chara braunii*. The detailed list of the abbreviations of the entries used can be found in Table S6.

At its base, the tree is partitioned into three monophyletic groups: The first clade includes all C4H sequences and has a convincing bootstrap support value of 99. The second group contains all CYP98 sequences as well as the three unconfirmed candidate sequences of *Chara braunii* (Chabra) and *Klebsormidium nitens* (Klenit). While the initial bootstrap value of the first branch of this clade is much lower at 78, the value also increases to 99 after the first two branches containing the unconfirmed genes split off. The last clade contains the five *Marchantia polymorpha* (Marpol) sequences MpC4H4‐8, which were initially annotated as C4H but already excluded from the characterization due to their lack of conserved motifs.

A large part of the C4H clade consists of eudicot C4Hs that often form clusters within families. For example, the six included Fabaceae C4Hs form a monophyletic group. The gymnosperms' C4Hs cluster closer to the bryophyte C4Hs, which are found on neighboring branches. The hornwort sequences from *Anthoceros agrestis* (Antagr) C4H1 and 2 are found closest to the gymnosperms and are followed by a smaller monophyletic group including the liverwort sequences. *Physcomitrium patens* (Phypat) C4H1‐6 follow that group and are separated into three final nodes containing two members each, from which one of each has been characterized in this study. *Marchantia polymorpha* C4H4‐8, which have been annotated as C4H but have not been included in this study due to a lack of conserved C4H motifs, form an outgroup without any other active CYP sequence.

The included CYP98 sequences follow a similar branching pattern, with one clade containing all the bryophyte CYP98 sequences clustering closely with the fern and lycophyte sequences, another containing the gymnosperm CYP98 sequences, and a last and most distant one containing the angiosperm CYP98 sequences.

### Heterologous expression of CYP73 and CYP98

The presence of the proteins under investigation in the microsome preparations after expression in the heterologous host *Saccharomyces cerevisiae* was proven by SDS‐PAGE and Western blot analysis (Figure S7).

The anti‐FLAG antibody blot targeting the cytochrome P450 resulted in strong signals for all CYPs at a size of around 55–65 kDa, according to the length of the respective CYP73 and CYP98 nucleotide sequences (1500–1700 base pairs).

The anti‐MYC antibody blot visualizes the protein encoded by the CsCPR sequence (GenBank AM980997.1) in the second integration site of the expression vector pESC‐Ura, which has a mass of 78.4 kDa. All microsome fractions analyzed with this antibody showed a band at that size, showing that the co‐expression of the two proteins has been successful.

### Biochemical characterization of CYP73 from bryophytes

The activity of CYP73 proteins expressed in yeast together with CPR from *Coleus scutellarioides* was tested in *in vitro* assays using microsome preparations with the substrates *t*‐cinnamate, 3‐phenylpropanoate and 3‐coumarate, of which only *t*‐cinnamate and 3‐coumarate were accepted by all active enzymes. 3‐Phenylpropanoate, lacking the double bond in the C_3_‐side chain, was not accepted as a substrate by any of the tested enzymes. As cinnamate is the proposed main substrate for C4H, all enzymes that were found to be active had a high affinity for and a high activity with this substrate. Both *Anthoceros agrestis* CYP73 proteins (AaC4H1, AaC4H2), all three *Marchantia polymorpha* (MpC4H1, MpC4H2, MpC4H3), and two of the *Physcomitrium patens* proteins (PpC4H1, PpC4H2) were proven to catalyze the formation of 4‐coumarate from *t*‐cinnamate. Additionally, the formation of caffeate from 3‐coumarate was demonstrated. The assays with microsomal proteins from yeast transformed with PpC4H3 or an empty vector showed no product formation (Figure 3).

**Figure 3:**
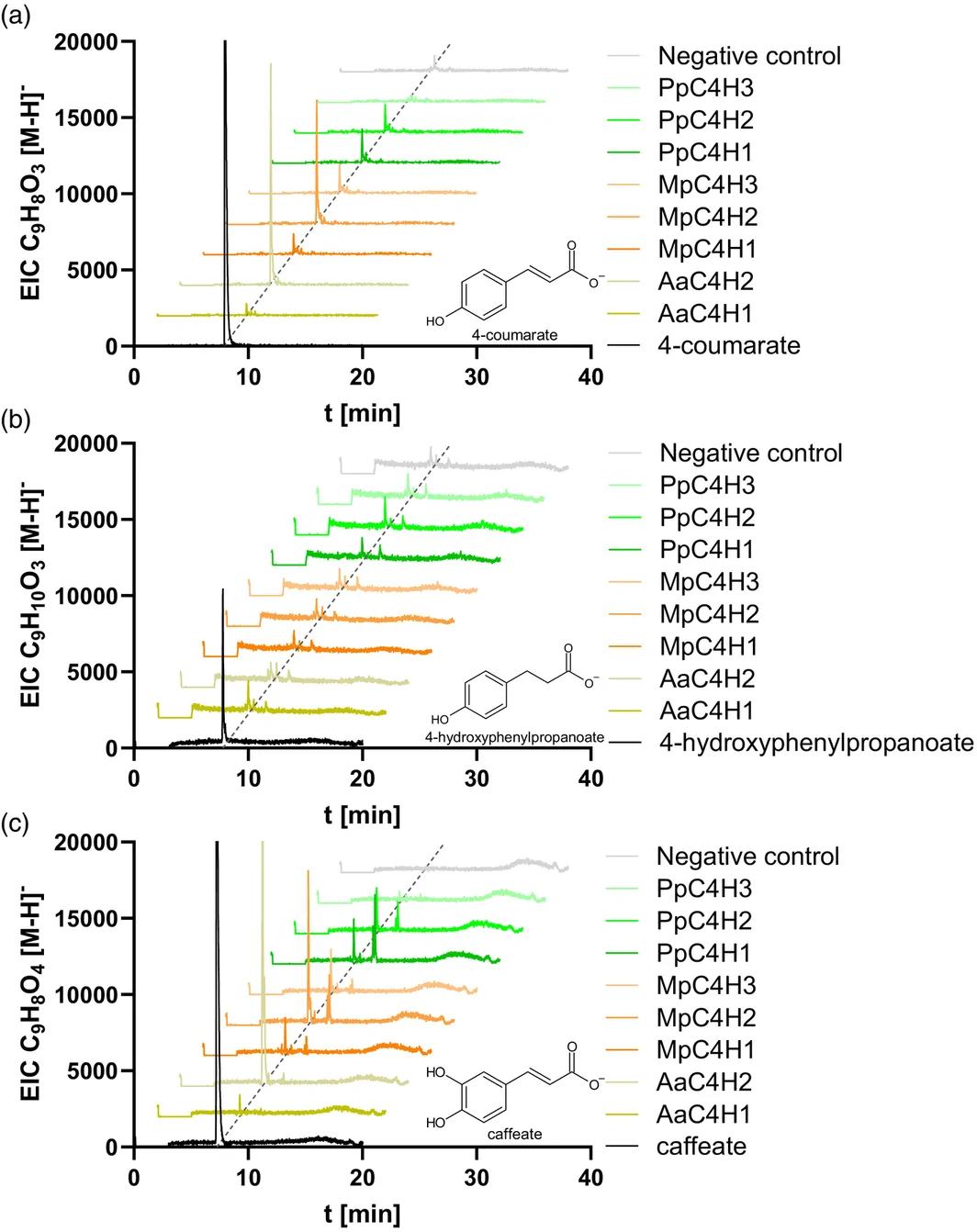
Extracted ion chromatograms (EIC) from LC–MS analyses of CYP73 enzyme assays with three different substrates. (a) Substrate conversion of *t*‐cinnamic acid to 4‐coumaric acid, (b) substrate conversion of 3‐phenylpropanoic acid without product formation, (c) conversion of 3‐coumaric acid to caffeic acid. The Y‐axis shows the molecular formula of the negatively charged ion of the product, the gray dotted line indicates the peak corresponding to the mass of the product, the colored lines depict the assays performed with the enzymes indicated in the legend on the right‐hand side.

All characterized CYP73 enzymes showed the lowest K_m_ values (therefore highest affinity) for *t*‐cinnamate, ranging from 1.10 μM to 74.28 μM, with an activity ranging from 8.03 fkat to 243.13 fkat. Each bryophyte species has at least one CYP73 isoform with a K_m_ value <20 μM and an activity >50 fkat. For *Anthoceros*, AaC4H2 shows a much lower K_m_ value and much higher activity than the previously investigated AaC4H1 (Wohl & Petersen, 2020). In the case of *Marchantia*, all three CYP73 show very similar K_m_ values and activities. For *Physomitrium*, PpC4H2 has a higher affinity towards *t*‐cinnamate than PpC4H1, but in contrast to the other enzymes, the activity of PpC4H1 with *t*‐cinnamate is higher than with 3‐coumarate (Table 1, Figure S8).

**Table 1:** K_m_ values and activities of all characterized CYP73 enzymes with *t*‐cinnamic acid and 3‐coumaric acid

Substrate	*t*‐cinnamic acid		3‐coumaric acid	
Enzyme	K_m_ [μM]	Activity [fkat]	K_m_ [μM]	Activity [fkat]
AaC4H1	43.95 ± 15.47	8.03 ± 2.44	346.33 ± 151.90	54.44 ± 24.93
AaC4H2	1.10 ± 0.31	139.97 ± 32.92	40.14 ± 8.09	281.73 ± 36.93
MpC4H1	13.70 ± 3.04	19.24 ± 14.79	125.63 ± 8.16	26.68 ± 6.36
MpC4H2	10.51 ± 1.58	59.87 ± 47.50	245.87 ± 48.52	180.97 ± 11.44
MpC4H3	11.70 ± 2.00	27.51 ± 0.90	202.53 ± 8.74	51.92 ± 4.95
PpC4H1	75.72 ± 16.27	243.13 ± 90.58	299.53 ± 33.20	138.93 ± 56.42
PpC4H2	17.24 ± 4.72	90.69 ± 51.72	130.52 ± 51.26	271.4 ± 95.03

All active CYP73s also accepted 3‐coumarate for hydroxylation to caffeate, but the K_m_ value for 3‐coumarate was generally higher. Interestingly, the activity was also higher with 3‐coumarate as compared to the supposed natural substrate *t*‐cinnamate. The K_m_ values for 3‐coumarate range from 40.14 μM to 378.90 μM, and the activity ranges from 26.68 fkat to 281.73 fkat. Again, AaC4H1 was more affine toward the substrate than AaC4H2 and showed a higher activity. For the three C4Hs from *Marchantia polymorpha*, the K_m_ values and activities with 3‐coumarate were in the same range. PpC4H2 was again the isoform with the higher affinity, and it also showed a higher activity than PpC4H1 (Table 1, Figure S8).

The products formed by 4‐hydroxylation of *t*‐cinnamate and 3‐coumarate were verified by LC–MS analyses to be 4‐coumarate and caffeate, respectively. In case of low product formation, incubation time courses for each sample were conducted and analyzed by HPLC to show the time‐dependent growth of the peak at the same retention time as the product's external standard (Figure S9).

### Biochemical characterization of CYP98 from bryophytes

A range of hydroxycinnamoyl acids, esters and amides was tested as substrates for the CYP98 candidates: *t*‐cinnamate, 3‐phenylpropanoate, 3‐coumarate, *N*‐(4‐coumaroyl)‐anthranilate, *N*‐(4‐coumaroyl)‐3′‐hydroxyanthranilate, *N*‐(4‐coumaroyl)‐tyramine, 4‐coumaroyl‐5’‐*O*‐shikimate, 4‐coumaroyl‐3’‐*O*‐quinate, 4‐coumaroyl‐5’‐*O*‐quinate, 4‐coumaroyl‐4′‐hydroxyphenyllactate, 4‐coumaroyl‐3′,4′‐dihydroxyphenyllactate, caffeoyl‐4‐hydroxyphenyllactate and 4‐coumaroyl‐4’‐*O*‐threonate (Figures S10 and S11). The assays with microsome preparations from yeasts transformed with an empty vector showed no product formation (Figure 4).

**Figure 4:**
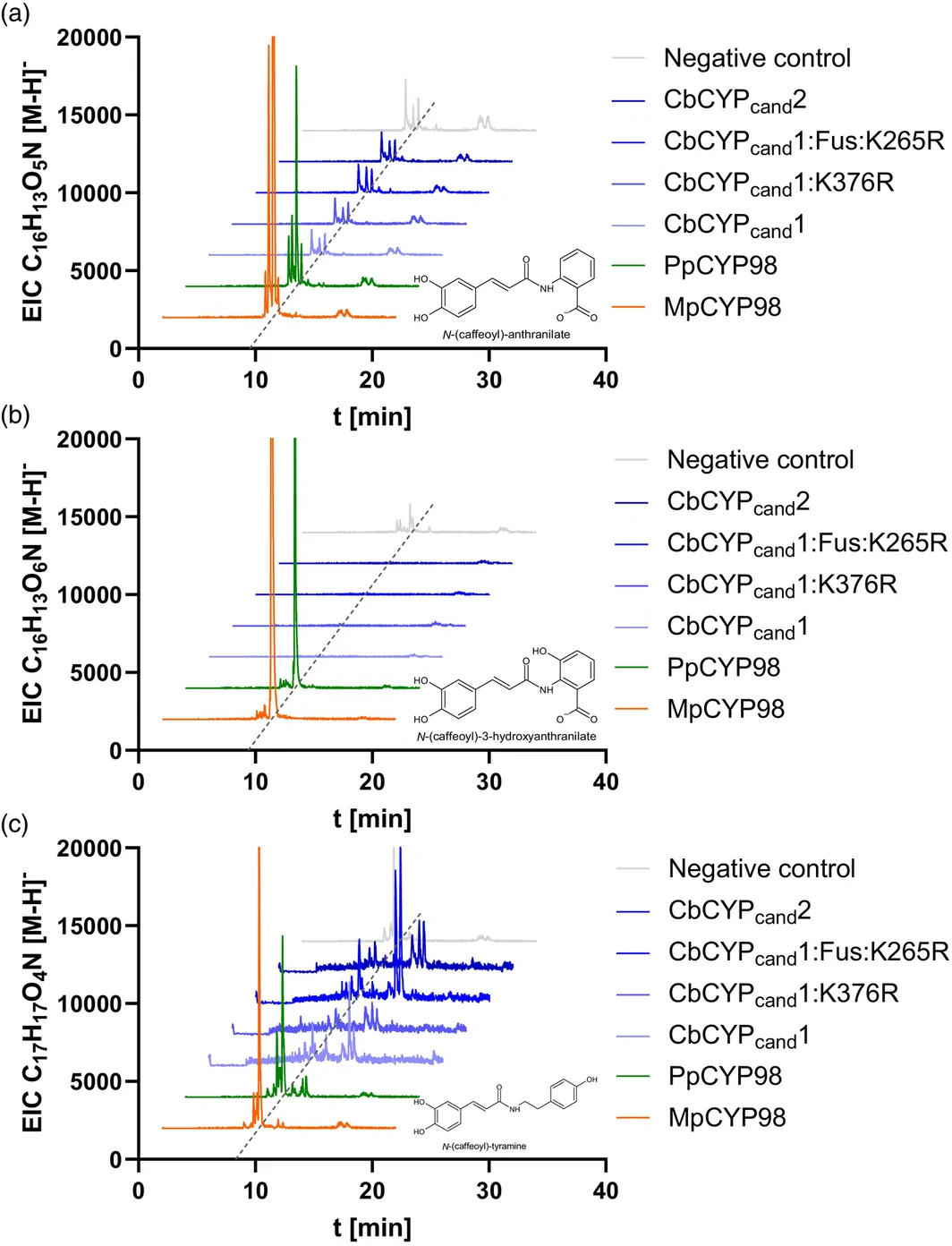
Extracted ion chromatograms (EIC) from LC–MS analyses of CYP98 enzyme assays with three different substrates. Substrate conversion of (a) *N*‐(4‐coumaroyl)‐anthranilic acid, (b) *N*‐(4‐coumaroyl)‐3′‐hydroxyanthranilic acid, (c) *N*‐(4‐coumaroyl)‐tyramine. The Y‐axis shows the molecular formula of the negatively charged ion of the product, the gray dotted line indicates the peak corresponding to the mass of the product, the colored lines depict the assays performed with the enzymes indicated in the legend on the right‐hand side. Product standards were not available.

*Marchantia polymorpha* MpCYP98 accepted *N*‐(4‐coumaroyl)‐anthranilate, *N*‐(4‐coumaroyl)‐3′‐hydroxyanthranilate and *N*‐(4‐coumaroyl)‐tyramine as substrates (Figure S12). The K_m_ values for the 4‐coumaroyl derivatives are similar, ranging from 22.19 μM to 31.16 μM. The activity is highest with *N*‐(4‐coumaroyl)‐3′‐hydroxyanthranilate at 600.20 fkat and lowest with *N*‐(4‐coumaroyl)‐tyramine (Table 2, Figure S13).

**Table 2:** K_m_ values and activities of all characterized CYP98 enzymes with *N*‐(4‐coumaroyl)‐anthranilate, *N*‐(4‐coumaroyl)‐3′‐hydroxyanthranilate and *N*‐(4‐coumaroyl)‐tyramine

Enzyme	MpCYP98		PpCYP98	
Substrate	K_m_ [μM]	Activity [fkat]	K_m_ [μM]	Activity [fkat]
*N*‐(4‐coumaroyl)‐anthranilate	22.19 ± 3.22	327.20 ± 68.42	83.47 ± 5.74	86.94 ± 18.20
*N*‐(4‐coumaroyl)‐3′‐hydroxyanthranilate	30.43 ± 6.04	600.20 ± 79.76	24.30 ± 2.33	57.08 ± 7.05
*N*‐(4‐coumaroyl)‐tyramine	31.16 ± 9.03	84.93 ± 21.27	Not quantifiable	Not quantifiable

*Physcomitrium patens* PpCYP98 accepted the amides *N*‐(4‐coumaroyl)‐anthranilate and *N*‐(4‐coumaroyl)‐3′‐hydroxyanthranilate for hydroxylation of the 4‐coumaroyl moiety. The affinity for *N*‐(4‐coumaroyl)‐3′‐hydroxyanthranilate is threefold higher at 24.30 μM compared to *N*‐(4‐coumaroyl)‐anthranilate, but the activity with *N*‐(4‐coumaroyl)‐anthranilate is higher at 86.94 fkat compared to 57.08 fkat (Table 2, Figure S13). *N*‐(4‐coumaroyl)‐tyramine was accepted as well, but only in traces.

### Activity assays with CYP candidates from *Chara braunii*

Both candidate genes identified in *Chara braunii* with the highest sequence similarity to CYP73, CbCYP_cand_1 and CbCYP_cand_2, were tested with all the above‐mentioned CYP73 and CYP98 substrates. However, both were found to be inactive in their native state.

After having identified the key differences in conserved motifs between CbCYP_cand_1 and other CYP73 genes, adjustments to the gene were made. First, a single base pair mutation was introduced via mutagenesis PCR, resulting in a K376R mutation (Table S1). After this change to a putatively essential amino acid residue, the translated protein still showed no conversion of *t*‐cinnamate, so further adjustments were made. A total of 10 interspaced sequences were identified that interrupt the alignment to other CYP73 genes (Figure 2). The modeled 3D structure showed loops that did not align with models of other CYP73. The amino acids in these loops were identified and removed from the coding sequence, retaining the mutation of lysine to arginine now in position 265. The new and shorter coding‐sequence CbCYP_cand_1:Fus1‐10:K265R (Table S1) was codon‐optimized and expressed in yeast. Unfortunately, the translated protein still showed no activity with any of the CYP73 or CYP98 substrates (Figure S10).

## DISCUSSION

### Phylogeny of CYP73 and CYP98

77 amino acid sequences encoding CYP73 or CYP98, including the sequences characterized in this work, were chosen to construct a phylogenetic tree to visualize the evolutionary relationship of the encoded enzymes. Overall, the phylogenetic tree followed an expected pattern of CYP73 and CYP98 protein sequences forming monophyletic clades, while the sequences within the clade tend to group according to their taxonomic relation.

The choice to characterize only three *Marchantia* C4Hs based on the presence of conserved motifs and overall length and similarity to other bryophyte C4Hs is also reflected in the distinct, distant second monophyletic branch formed by the liverwort's genes MpC4H4‐8, which might be erroneously annotated as C4H. All other bryophyte C4H sequences were grouped closely together on multiple branches within the CYP73 clade, with *Anthoceros agrestis* (Antagr) homologous sequences being located closer to the tracheophyte sequences. This illustrates the evolutionary relationship between these groups, probably reflecting their respective times of emergence.

A similar pattern is evident within the monophyletic CYP98 clade, which by themselves also forms a monophyletic system, where seed plant sequences form a monophyletic branch, distinct from branches containing sequences from non‐seed plants. The CYP98 sequence from the lycophyte *Diphasiastrum complanatum* (Dipcom) is more closely related to the CYP98 sequences found in bryophytes, whereas the CYP98 sequences found in the fern *Ceratopteris richardii* (Cerric) and putative CYP sequences found in algal genomes show earlier branching off. The distinct clades of seed plant CYP98 and non‐seed plant CYP98 are also reflected in their substrate acceptance. The bryophyte CYP98 genes characterized in this study catalyze the hydroxylation of 4‐coumaroyl amides, while the seed plant CYP98 genes are involved in the biosynthesis of 4‐coumaroyl esters, e.g., as a precursor for lignin. This neo‐functionalization supports the evolving functions of CYP98 as a key family during the biosynthesis of more complexly substituted phenolic compounds.

CYP73 and CYP98 belong to the same clan 71, which is the largest of the ten clans identified in plants by Nelson et al. (2004). While CbCYP_cand_1 was initially identified as a potential CYP73 gene, the phylogenetic analysis grouped it within the CYP98 clade, which shows high levels of similarity between these two CYP families from the same clade. But since neither the *Chara braunii* candidate nor the proposed CYP98 candidate from *Klebsormidium nitens* (Klenit) were proven to be active, it is not yet possible to verify their placement in the phylogenetic tree.

### CYP73 and C4H activity

For each of the chosen bryophyte species, several convincing CYP73 sequences were retrieved, but not all heterologously expressed proteins showed catalytic activity in our hands with the chosen C4H substrates (*t*‐cinnamate, 3‐phenylpropanoate, 3‐coumarate). All proteins carrying the conserved C4H motif AAIETT were active, while PpC4H3 with a distorted motif (AAIEST) was inactive. Here, mutagenesis to exchange the serine residue for threonine might be done to show whether the activity could be restored. Other putative CYP73 sequences without this motif or with a strongly changed amino acid composition in this motif have been excluded beforehand from further analysis. None of the active C4H enzymes accepted 3‐phenylpropanoate as substrate, suggesting the necessity of the double bond in the C_3_ side chain for the substrate to be accepted. Another important feature of the ER membrane‐bound cytochrome P450s seemed to be the membrane anchor that is necessary to fasten the protein in the membrane and position the main part of the protein into the cytosol. Here, it can interact with NADPH:cytochrome P450 reductase (CPR), which is also anchored in the ER membrane. The membrane anchor could not be predicted for all investigated cytochrome P450 sequences using DeepTMHMM. Especially the missing membrane anchors for MpC4H3 and PpC4H3 should be questioned. The prediction from DeepTMHMM might show some false negatives as compared to the sequence and 3D structure. All the AlphaFold3 predicted structures show the same corkscrew‐like structure in their *N*‐terminal region consisting of almost exclusively hydrophobic amino acids. The MpC4H1 and MpC4H2 models even showed two of these structures back‐to‐back (Figure S5). The proline‐rich hinge following the membrane‐anchor also varied between each candidate, making the corkscrew‐like membrane anchor stick out at different angles.

All investigated bryophytic CYP73 (except the inactive PpC4H3) have their highest affinity for the name‐giving substrate cinnamate. However, except for one enzyme, the activity with 3‐coumarate is higher. The K_m_ values and activities (Table 1) are in line with other characterized CYP73s, which range from <1 μM to 1 mM (https://www.brenda‐enzymes.org/). In our study, the K_m_ for cinnamate is in the range of 7‐ to 24‐fold lower than for 3‐coumarate – demonstrating the higher affinity for cinnamate. In contrast, the activities are maximally threefold higher with 3‐coumarate. For PpC4H1, the affinity towards cinnamate is overall the lowest and only four times higher than for 3‐coumarate. In this case, however, the activity with cinnamate is 1.5‐fold higher than with 3‐coumarate. Another exception is AaC4H1, where the activity is seven times higher with 3‐coumarate. This enzyme, however, shows the lowest activity overall, resulting in stronger factoring of the standard deviation. Overall, the activity is the least consistent metric between assays of the same enzyme, because the percentages of all membrane‐bound proteins vary greatly between each cell harvest and therefore during the process of preparing microsomal fractions. Thus, even if the microsome preparation is always diluted to the same total protein concentration of 1 mg/ml, the amount of CYP73 in each harvest might be different from the last. This cannot be standardized easily since a specific cytochrome P450 cannot be quantified in the membrane preparation.

Given the consistency in substrate affinity and conserved motifs, it is reasonable to classify all catalytically active enzymes described here as bona fide CYP73 with C4H activity. Although the activity favors 3‐coumarate in all cases but one, the margin for this is much lower than the preference in affinity towards *t*‐cinnamate. The same conclusion was reached for PpC4H2 and MpC4H2 by Knosp et al. (2024). However, this study did not include kinetic parameters or the other orthologs from these organisms. Recently, MpC4H1‐3 were kinetically characterized with *t*‐cinnamate, and the resulting K_m_ values are very close to those presented in this study (Yang et al., 2025). The authors, however, did not include more substrates or other C4H enzymes from bryophytes. This makes our study the first comprehensive study in which multiple candidate genes from all clades of bryophytes have been expressed under the same conditions and characterized with multiple substrates. Our results confirm that multiple isoforms of CYP73 are active in all bryophyte clades, indicating a strong need for C4H activity during their evolution and during terrestrialization. Even though all CYP73 orthologs accept the same substrates, their differential expression patterns might hint at different functions for the organism. One copy of C4H could serve as a failsafe in case the expression of another copy gets inhibited. In *Physcomitrium patens*, a gene duplication event might have also led to the increase in CYP73 orthologs. Still, it is noteworthy that each bryophyte class has multiple active CYP73s, even though no CYP73 orthologs or activity could be found in the closest water‐living relative.

Although a classical vascular system, as found in the tracheophytes, is missing in bryophytes, conducting strands surrounded by thick‐walled but unlignified cells are found in mosses. The formation of phenolic compounds involved in cell wall stabilization is a feature already found in streptophyte algae (Rieseberg et al., 2023). In addition, cuticle formation has been shown to be dependent on phenolic compounds (Renault, Alber, et al., 2017).

Flavonoids that are synthesized downstream of the general phenylpropanoid pathway, which ends with *p*‐coumaroyl‐CoA, are found in liverworts (e.g., riccionidin A, auronidins; Kunz et al., 1993, Berland et al., 2019) and mosses, but not in hornworts (Yonekura‐Sakakibara et al., 2019). Moreover, flavonoids as well as genes tentatively identified as enzymes of flavonoid biosynthesis were detected in cyanobacteria, rhodophytes, chlorophytes, haptophytes and ochrophytes (heterokontophyta). Whether these putative flavonoid biosynthesis genes really are involved in this biosynthetic pathway has not been tested (Goiris et al., 2014). Flavonoids as well as putative 4CL and chalcone synthase homologs were reported to be present in *Penium margaritaceum* (Zygnematophyceae) (Jiao et al., 2020). However, the full biosynthetic pathway involved in the formation of these flavonoids is unknown. Recently, Kunz et al. (2026) showed that UV irradiation of *Mesotaenium endlicherianum* upregulated several genes, among them a 4CL‐like sequence. While the biochemical activity is unknown, it showed higher similarities to fatty acid AMP‐ligases than to 4CL. Metabolomic studies of this alga identified coumarin‐like compounds as possible UV absorbants. In our own research with *M. endlicherianum*, two 4CL homologs were identified (Me4CL1: *N*‐terminus Me1_v2_0037890.1, *C*‐terminus Me1_v2_0103110.1; Me4CL2: Me1_v2_0064910.5), of which one (Me4CL2, renamed MeOCL2) catalyzed the activation of oxalic acid as CoA‐thioester (Marks, 2026). Kunz et al. (2026) came to the conclusion, that zygnematophytes such as *M. endlicherianum* do not synthesize their phenolic compounds through the canonical phenylpropanoid pathway of tracheophytes and instead make use of other currently unknown enzymes, which could be encoded by members of the UV‐upregulated pool of candidate genes.

The lack of convincing CYP73 candidates in *Chara braunii* is in line with previous studies that already found no CYP73 gene candidates *in silico* (de Vries et al., 2021). Our approach provides *in vitro* support for the lack of C4H activity in the closest CYP73 match found in *Chara braunii*. Even after adjusting the native sequence with minor or even major changes to match other consensus CYP73 sequences, no catalytic conversion of *t*‐cinnamate to 4‐coumarate could be measured. This result raises the question of how close the Charophyta were to C4H activity before terrestrialization, and whether a major shift was necessary.

Since no substrate was found that was converted by CbCYP_cand_1, a clue to its evolutionary standing could not be deduced. For example, the first enzyme of the phenylpropanoid pathway, phenylalanine ammonia‐lyase (PAL), is said to have emerged from histidine ammonia‐lyase (HAL) (Wu et al., 2014). However, a recent study showed that the enzymes from *Chara braunii*, which resembled HALs more closely, already had PAL activity, making it one of the first PALs characterized from an aquatic plant (Schwarze & Petersen, 2024). This is why enzymes from charophytes have an important standing in the elucidation of the evolution of the phenylpropanoid pathway during terrestrialization. Since both PAL and 4CL (the third enzyme of the phenylpropanoid pathway) are found in *Chara braunii* (Schwarze & Petersen, 2024), the presence of CYP73 genes was a reasonable expectation. However, as none of the candidate genes exhibited the desired reaction, it is possible that *Chara braunii* harbors a gene responsible for C4H activity that resembles known CYP73 genes even less. Alternatively, *Chara* could provide 4‐coumarate as a substrate for 4CL in a completely different manner.

### CYP98 and C3H activity

CYP98 has been detected as the “missing link” in monolignol and lignin biosynthesis, as the enzyme hydroxylating 4‐coumaroylshikimate to caffeoylshikimate, which is then cleaved by caffeoylshikimate esterase (CSE) to deliver caffeic acid moieties for further transformations (Schoch et al., 2001; Vanholme et al., 2013). These caffeic acid moieties are essential precursors for the formation of caffeyl, coniferyl, and sinapyl alcohols as building blocks for lignin (Boerjan et al., 2003). Besides this, CYP98 isoforms are known to specifically *meta*‐hydroxylate 4‐coumaroyl moieties in other esters or amides, e.g., in the formation of rosmarinic acid, caffeoylspermidine derivatives, or flavonoids (Eberle et al., 2009; Zhou et al., 2025).

Since bryophytes lack lignin, the native function of CYP98 in bryophytes remains unclear. However, the correct formation of a cuticle has been shown to be impaired after deletion of the only CYP98 gene in *Physcomitrium patens* (Pp3c22_19010; PpCYP98A34). Here, CYP98 has been described to transform 4‐coumaroyl‐4’‐*O*‐threonate to caffeoyl‐4‐*O*‐threonate (Renault, Alber, et al., 2017). Later, the same PpCYP98 was shown to prefer *N*‐(4‐coumaroyl)‐anthranilate but also to hydroxylate 4‐coumaroylshikimate (Alber et al., 2019). PpCYP98A34 could not complement an AtCYP98A3 loss‐of‐function mutant of *Arabidopsis thaliana*, demonstrating the low affinity and activity for 4‐coumaroylshikimate. In our hands, PpCYP98 did not convert 4‐coumaroyl‐5’‐*O*‐shikimate but showed efficient *meta*‐hydroxylation of *N*‐(4‐coumaroyl)‐anthranilate and *N*‐(4‐coumaroyl)‐3′‐hydroxyanthranilate with preference for the latter. These compounds, however, have not been found to date in this species or the other bryophytes under investigation, and a physiological function is unknown. CYP98 from *Marchantia polymorpha* additionally hydroxylates *N*‐(4‐coumaroyl)‐tyramine, whereas CYP98 from *Anthoceros agrestis* was shown to accept *N*‐(4‐coumaroyl)‐anthranilate, *N*‐(4‐coumaroyl)‐3′‐hydroxyanthranilate, and *N*‐(4‐coumaroyl)‐tyramine but only acted poorly in the hydroxylation of 4‐coumaroyl‐5’‐*O*‐shikimate and 4‐coumaroyl‐4’‐hydroxyphenyllactate (Ernst et al., 2022). The acceptance of the latter substrate would point to the formation of rosmarinic acid, which is accumulated in *Anthoceros agrestis*. However, to date, no hydroxycinnamoyltransferase transferring 4‐coumarate to 4‐hydroxyphenyllactate is known in this hornwort. Kriegshauser et al. (2021) showed that 4‐coumaroylshikimate is present in all three bryophytes (*A. agrestis*, *M. polymorpha*, *P. patens*), while caffeoylshikimate was not detected in the extract of *P. patens* gametophores. 4‐Coumaroyl‐4’‐*O*‐threonate derivatives were, on the other hand, only found in *P. patens*. Biochemical characterization showed that the K_m_ values are very similar for all substrates determined for MpCYP98, while PpCYP98 preferred *N*‐(4‐coumaroyl)‐3′‐hydroxyanthranilate. Also, the previously characterized AaCYP98 showed a preference for *N*‐(4‐coumaroyl)‐3′‐hydroxyanthranilate, followed by *N*‐(4‐coumaroyl)‐anthranilate and *N‐*(4‐coumaroyl)‐tyramine (Ernst et al., 2022). This difference in substrate acceptance of CYP98 from bryophytes versus the CYP98 from tracheophytes is also mirrored by the distinct clades in which they appear in the phylogenetic tree (Figure 1).

### Cytochrome P450 in *Chara braunii*

Previous publications have found no orthologous genes for CYP73 or CYP98 in *Chara braunii*, even though some potential CYP98 gene orthologs were found in other streptophyte algae (de Vries et al., 2017, 2021; Knosp et al., 2024). We identified two candidate genes with moderate homology to known CYP73/CYP98 sequences which, however, could not be safely categorized to one of the CYP families. Even though the phylogenetic analysis placed them in the CYP98 clade, we named them CbCYP_cand_1 and 2 and tested the heterologously expressed proteins with substrates for both enzyme families. Despite proof of successful expression, no product formation with any of the applied CYP73 or CYP98 substrates could be detected. This result underlines the proposed post‐terrestrialization emergence of many of the active CYP genes. Since the *Chara braunii* CYP candidate sequences did not show all the conserved motifs that are expected in CYP73/CYP98 genes, this outcome might support the importance of these domains.

We introduced changes into CbCYP_cand_1 by single‐site mutation and by deletion of obviously superfluous sequence stretches via gene synthesis. These were aimed at investigating how close CbCYP_cand_1 was to having C4H or C3H/C3′H activity. The single mutation at position 376 from l‐lysine to l‐arginine was inspired by the findings of Knosp *et al*. in (2024). They proved that this single mutation away from l‐arginine could result in total loss of C4H activity. For CbCYP_cand_1, the single mutation did not lead to the gain of hydroxylation activity in CbCYP_cand_1:K376R. Since the alignment of native CbCYP_cand_1 with known C4H genes showed the important l‐serine of the RSxxxQ triad identified by Knosp et al. (2024) directly after k376 and l‐glutamine after a stretch of 20 amino acids, which did not align with other CYP73 sequences, we decided to create a mutant that excludes these amino acids. The idea of these stretches being introns was quickly dismissed, as all used sequences from *Chara* stem from RNA‐seq data, which should have all introns eliminated. Still, the sequence was almost 500 bp longer than the other CYP73 sequences, and after careful evaluation of multiple alignments, 10 stretches in total were eliminated from the CbCYP_cand_1 sequence. After this, the alignment of CbCYP_cand_1:Fus1‐10:K265R showed major resemblance to other CYP73 sequences, both in the conserved sequences now including the RSxxxQ motif as well as in the 3D structure, which now had no more loops sticking out of the alignment (Figure 2). Unfortunately, even after this effort to align the most similar *Chara* sequence with the already characterized and studied bryophyte CYP73 genes, the translated protein of CbCYP_cand_1:Fus1‐10:K265R showed no activity in our hands with any verified CYP73 or CYP98 substrate.

Despite the fact that the first (PAL) and the third (4CL) enzyme of the general phenylpropanoid pathway have been shown to be active in *Chara braunii*, though with a lower catalytic efficiency when compared to bryophyte or tracheophyte activities (Schwarze & Petersen, 2024), the presence of a full phenylpropanoid pathway in *Chara braunii* remains elusive. The 4‐hydroxylation step from cinnamate to 4‐coumarate could not be shown, maybe because other enzymes – not yet known – might be active here. To date, no convincing CYP98 candidate sequence could be retrieved from streptophytic algae, although *Klebsormidium* species were reported to contain a putative CYP98 sequence (de Vries et al., 2017, 2021). Here, however, biochemical data have not been collected. An alignment of this sequence from *Klebsormidium nitens* (Phycocosm kfl00035_0080_v1.1) with CYP98 from *Anthoceros agrestis* (QPI70542.1) displayed a rather low identity of 30.1% and a similarity of 45.0% on an amino acid basis. In general, conservation rates within the same CYP family are usually higher, raising a question about the annotation of the sequence from *Klebsormidium*. It will have to be shown whether it encodes an active 4‐coumarate ester/amide 3‐hydroxylase.

If CYP73 and/or CYP98 activity could be proven with any substrate in one of the candidate genes from *Chara*, it could also help to identify a phenolic compound that could be considered as an “ancestral polymer” and predecessor of the diverse polymers found in embryophytes (Renault et al., 2019). But the lack of activity in *Chara* CYP‐candidates with the substrates offered in our enzyme assays and the fact that, to date, none of the hydroxylation products of substrates accepted in our studies by MpCYP98 and PpCYP98 were found in metabolomic studies, makes further investigation into the evolution of the phenylpropanoid pathway necessary.

In conclusion, this study shows that CYP73 and CYP98 are key cytochrome P450 monooxygenases in phenolic metabolism and that they are active in bryophytes. Gene sequences identified in *Chara braunii* as a member of the streptophyte algae could be heterologously expressed but failed to catalyze reactions typical of CYP73 or CYP98 in our hands. Comparative biochemical characterization of the bryophyte enzymes showed that CYP73 displays cinnamate 4‐hydroxylase activity as expected, with cinnamate as the best substrate, but also with a high turnover of 3‐coumarate. The affinities are slightly lower than those reported for C4Hs from most seed plants (<1 μM to 1 mM; www.brenda‐enzymes.org). Each of the investigated bryophytes contains several CYP73 isoforms, while only one ortholog of CYP98 is present in each species. The identified CYP98 genes from bryophytes encode active 3‐coumarate ester/amide 3‐hydroxylases, but the substrate acceptance was shown to be different from the respective enzymes from seed plants, as 4‐coumaryol‐5’‐*O*‐shikimate was not accepted as a substrate. Instead, the hydroxylases acted best on 4‐coumaroyl amides. The relevance of this finding and the pathway leading to specialized products with a caffeoyl substitution pattern in bryophytes should be clarified further.

## MATERIALS AND METHODS

### Identification and bioinformatic characterization of CYP73 and CYP98 sequences

To identify sequences encoding CYP73 and CYP98, the amino acid sequences for CYP73 (GenBank QHF16161.1) and CYP98 (GenBank QPI70542.1) from the hornwort *Anthoceros agrestis* were used as bait sequences (Ernst et al., 2022; Wohl & Petersen, 2020). For *Anthoceros agrestis* the 1kP database (https://db.cngb.org/onekp/) was searched; for *Marchantia polymorpha* and *Physcomitrium patens*, the Phytozome 13 database (https://phytozome‐next.jgi.doe.gov/) was used; and for *Chara braunii*, the ORCAE (https://bioinformatics.psb.ugent.be/orcae/) databank and Phycocosm (https://phycocosm.jgi.doe.gov/phycocosm/home) were used, and the search was supported with a text search for “cinnamate 4‐hydroxylase”.

Expression data for *Anthoceros agrestis* and *Physcomitrium patens* C4H and CYP98 orthologs were taken from PEATmoss (https://peatmoss.plantcode.cup.uni‐freiburg.de) and for *Marchantia polymorpha* MarpolBase (https://mbex.marchantia.info) was used.

Pairwise alignments were performed using EMBOSS Needle (https://www.bioinformatics.nl/cgi‐bin/emboss/needle). Multiple sequence alignments were performed with CLC Sequence Viewer 8.0 (Qiagen) and Clustal Omega (https://www.ebi.ac.uk/jdispatcher/msa/clustalo). Transmembrane helices and signal peptides were predicted by DeepTMHMM 1.0.57 (https://dtu.biolib.com/DeepTMHMM) (Hallgren et al., 2022). The molecular mass and the isoelectric points of the translated proteins were calculated using the ProtParam tool from ExPASY (https://web.expasy.org/protparam/).

Phylogenetic trees were constructed with the Maximum Likelihood method using MEGA12 (Tamura et al., 2021) with the LG algorithm with 1000 bootstraps and default settings. The LG model was chosen based on IQtree3 ModelFinder (Kalyaanamoorthy et al., 2017; Wong et al., 2026). Species abbreviations used for the tree are explained in Table S6.

3D models of proteins were predicted with the AlphaFold 3 model (https://alphafoldserver.com) to check for structural similarities with other CYP enzymes. For the alignments of the 3D structures, Discovery Studio 2024 Client (BIOVIA) was used.

### Cultivation of *Saccharomyces cerevisiae*

The untransformed *Saccharomyces cerevisiae* YPH501 strain (Agilent, Waldbronn, Germany) was maintained on YPD medium (Dymond, 2013) and subcultured every 2–4 weeks. Transformed strains were kept on SCD_‐Ura_‐dropout plates (Gietz & Woods, 2002) and subcultured at least once a week for a maximum of 4 weeks.

### Vector preparation

Coding sequences plus suitable 5′ and 3′ restriction sites were synthesized with codon optimization for expression in *Saccharomyces cerevisiae* and were delivered in a pUC57‐BsaI free vector (BioCat, Heidelberg, Germany). They were released from the vector by restriction digestion with EcoRI and NotI, specific for multiple‐cloning‐site I (MCS I) of the expression plasmid pESC‐Ura (Agilent, Waldbronn, Germany), which already contained a NADPH:cytochrome P450 reductase sequence from *Coleus scutellarioides* (GenBank AM980997.1) in MCS II (Eberle et al., 2009). After ligation of the coding sequences into the expression vector pESC‐Ura_CsCPR with T4‐DNA ligase, the plasmids were transferred into *E. coli* EZ strain (Qiagen, Hilden, Germany) for amplification. The plasmids were isolated, and the sequence was verified (Seqlab, Göttingen, Germany) for transformation of yeast.

For the *Chara braunii* sequences and PpCYP98, a PCR reaction using the AccuPrime™ *Taq* DNA Polymerase standard protocol (Invitrogen, Carlsbad, CA, USA) was performed using primers designed with a 19 bp overhang both with the CDS and with MCS I of pESC‐Ura for homologous recombination. The pESC‐Ura vector was linearized with EcoRI and NotI. The PCR amplicon and the digested vector were purified and used to transform *E. coli* EZ (Qiagen, Hilden, Germany) for homologous recombination without prior ligation. All primer sequences are listed in Table S7.

For CbCYP_cand_1:K376R, only a single base pair adjustment was made. For this, PCR with mutagenesis primers (Table S7) was conducted using the CloneAmp™ HiFi PCR Premix (Takara Bio, Mountain View, CA, USA). The elongation time was adjusted for full vector amplification of the pESC‐Ura vector containing CbCYP_cand_1 in MCS I and CsCPR in MCS II. The plasmid showing the desired mutation was transferred into *E. coli* EZ strain (Qiagen, Hilden, Germany) for amplification and used for transformation of yeast. All PCR setups can be seen in Tables S8 and S9.

### Transformation of yeast

The yeast strain YPH501 was transformed essentially according to the rapid transformation protocol of Gietz and Woods (2002). Salmon sperm carrier DNA was denatured at 95°C for 5 min. Yeast colonies, approximately the size of a pinhead, were picked from a plate not older than 5 days and transferred into 1 ml of deionized H_2_O. The suspension was spun down at 8000 ***g*** for 30 sec. After removal of the supernatant, 240 μl PEG 4000 (50% w/v) was added, followed by 36 μl lithium acetate (1 M) and 50 μl salmon sperm carrier DNA (2 mg/ml). Lastly, 34 μl plasmid (50 ng/μl) was mixed in carefully. A heat shock was conducted in a thermomixer at 42°C while shaking for at least 1 h, and then the mixture was centrifuged at 8000 ***g*** for 1 min. The pellet was resuspended in 200 μl sterile H_2_O, plated out on an SCD_‐Ura_‐dropout plate, and incubated at 30°C for 72 h.

To check for successful transformation, colony PCR was performed. A single colony of the transformed strain was picked with a toothpick from the SCD_‐Ura_‐dropout plate and suspended in 100 μl 100 mM lithium acetate with 1% sodium dodecyl sulfate. The same toothpick was stricken out again on a fresh SCD_‐Ura_‐dropout plate to preserve the clone. The suspension was heated to 70°C for 5 min, and then 300 μl 96% EtOH was added. After stirring, the cells were spun down at 15 000 ***g*** for 3 min, the supernatant was discarded, and the pellet was washed twice with 70% ethanol. After drying, the pellet was resuspended in 40 μl sterile H_2_O. 2 μl of this solution was used as a template for a PCR reaction using primers specifically designed for the two MCS (Table S7). PCR was performed according to the GoTaq® G2 DNA Polymerase protocol (Promega, Madison, CA, USA) (Tables S8 and S9), and the amplicons were checked by agarose gel electrophoresis.

### Expression in yeast

From transformed yeast strains growing on SCD_‐Ura_‐dropout plates, a pinhead‐sized group of colonies was added to 95 ml SCD_‐Ura_‐medium in a 500 ml‐baffled flask. The inoculated culture was incubated at 30°C with shaking at 140 rpm for 72 hours. The yeast cells were harvested by centrifugation at 3000 ***g*** and 4°C for 5 min, and the supernatant was discarded. The pellet was resuspended in 95 ml SCG_‐Ura_‐medium. To this, 95 μl FeSO_4_ (45 mM) and 95 μl 5‐aminolevulinic acid (200 mM) were added, and the cultures were incubated for another 24 h at 30°C while shaking at 140 rpm.

Yeast cells were collected by centrifugation at 3000 ***g*** and 4°C for 5 min and then resuspended in 10 ml 0.1 M Tris–HCl‐buffer (pH 7.5) containing 1 mM dithiothreitol (DTT) and 1 mM diethyldithiocarbamate (DIECA). The cell suspension was centrifuged again at 3000 ***g*** and 4°C for 5 min, and the supernatant was discarded. The resulting pellet was then resuspended in 1.5 ml of the same buffer and transferred into a 7 ml MiniLys‐tube containing 2.5 g acid washed glass beads (⌀ 425–600 μm, Merck). The tube was vigorously shaken at level 2 in a benchtop homogenizer (MiniLys) six times for 30 sec, with 30 sec of cooling on ice in between. The supernatant was transferred into a sterile 2 ml reaction tube. The beads were washed with 0.5 ml buffer by stirring vigorously and then letting the beads settle on ice again. The supernatant was combined with the first supernatant, and the tube was centrifuged at 5000 ***g*** and 4°C for 20 min. 1 M MgCl_2_ was added dropwise to the resulting supernatant, adjusting it to a final concentration of 50 mM MgCl_2_ while stirring on ice for 20 min. Afterwards, the soluble protein was separated from the membrane fraction by centrifugation at 60000 ***g*** and 4°C for 20 min, and the supernatant was discarded. The pellet was washed with 0.3 ml of the same buffer and then resuspended in 1 ml of buffer. The suspension was homogenized in a glass homogenizer (Potter‐Elvehjem), leading to the microsome preparation. The protein concentration was determined as described by Bradford (1976) and adjusted to 1 mg/ml with the same buffer for use in the standard assays.

### Western blot

Protein samples were separated by SDS‐PAGE (Laemmli, 1970). Successful expression was monitored by Western Blot analysis according to Mahmood and Yang (2012) with the buffer system from Towbin et al. (1979) using an anti‐FLAG antibody (Invitrogen; FG4R) to stain the FLAG‐tagged CYP protein and an anti‐MYC antibody (Progen; IgG1 9E10) for MYC‐tagged CsCPR. Goat anti‐mouse IgG Fc alkaline phosphatase conjugates (Life Technologies; A16087) were applied as secondary antibodies. Localization of antibodies coupled to alkaline phosphatase was visualized by nitro blue tetrazolium chloride (NBT)/5‐bromo‐4‐chloro‐3‐indolyl‐phosphate (BCIP) staining following standard protocols (https://www.sysy.com/protocols/westernblot‐ap‐detection).

### Standard enzyme assays for cytochrome P450 enzymes

The standard assay uses 7.5 μl of varying substrates (dissolved in 50% methanol, leading to 3% methanol per assay and final substrate concentrations of 0.6–600 μM depending on the stock concentration), 12.5 μl NADPH (2.5 mM) as co‐substrate and 50 μl microsomal protein (1 mg/ml) in a total volume of 125 μl 0.1 M Tris–HCl buffer (pH 7.5). The reaction was started by the addition of the microsome preparation. The samples were incubated in a thermomixer at 25°C while shaking at 1200 rpm. The incubation time was between 5 min and 120 min, depending on the activity of the enzyme, and was stopped by adding 50 μl HCl (6 N) and chilling on ice. The assays were extracted twice by adding 500 μl ethyl acetate, stirring vigorously, and then centrifuging at 16800 ***g*** for 5 min. The ethyl acetate phases were pooled and evaporated. The residues were first dissolved in 50 μl methanol with 0.01% H_3_PO_4_, and then 50 μl aqueous 0.01% H_3_PO_4_ was added. After centrifugation for 10 min the supernatant was analyzed by HPLC (Hitachi Chromaster system with a Hypersil ODS column; 250 × ⌀ 4 mm, 5 μm with 20 mm precolumn) using isocratic elution at a flow rate of 1 ml/min with 20–50% methanol +0.01% H_3_PO_4_ as mobile phase and monitored by a DAD system at 280–333 nm (see Table S10 for individual settings). LC–MS analysis was performed with an Agilent Technologies HPLC 1260 with a Multospher 120 RP18 column (250 × 2 mm, particle size 5 μm; CS‐Chromatographie Service) using a solvent system of solvent A (0.1% (v/v) aqueous formic acid) and solvent B (0.1% (v/v) formic acid in acetonitrile). For the verification of product formation in enzyme assays, the gradient was as follows: 0–10 min 5% B → 100% B, 10–15 min 100% B, 15–20 min 5% B, flow rate of 0.5 ml/min at 25°C. Detection was performed with a DAD at 190–400 nm and a micrOTOF‐Q III (Bruker Daltonics) with an ESI source calibrated with 5 mM CHNaO_2_ in the negative ion mode. Authentic standards (0.25–1.0 nmol) were injected for identification and quantification, if available. If not, the product was not quantified and was confirmed via the expected mass and fragmentation pattern.

Control assays contained microsomal proteins harvested from a yeast strain transformed with a pESC‐Ura vector only containing the CsCPR coding‐sequence in MCS II, but no CYP coding‐sequence in MCS I.

*t*‐Cinnamate, 3‐phenylpropanoate and 3‐coumarate were used as substrates in assays containing CYP73. For CYP98 assays the following substrates were used: *t*‐cinnamate, 3‐phenylpropanoate, 3‐coumarate, *N*‐(4‐coumaroyl)‐anthranilate, *N*‐(4‐coumaroyl)‐3′‐hydroxyanthranilate, *N*‐(4‐coumaroyl)‐tyramine, 4‐coumaroyl‐5’‐*O*‐shikimate, 4‐coumaroyl‐3’‐*O*‐quinate, 4‐coumaroyl‐5’‐*O*‐quinate, 4‐coumaroyl‐4′‐hydroxyphenyllactate, 4‐coumaroyl‐3′,4′‐dihydroxyphenyllactate, caffeoyl‐4‐hydroxyphenyllactate and 4‐coumaroyl‐4’‐*O*‐threonate (Figure S11).

### Evaluation of data

For determination of K_m_ values, assays were performed with increasing substrate concentrations, and the resulting activity was plotted against the substrate concentration in GraphPad Prism 11.0.0 and analyzed with the Michaelis–Menten equation. For each enzyme, at least three distinct harvests were tested, each with three assay replicates (*n* = 3). One K_m_‐value was calculated from each *n* = 3 replicate, and the mean of three different K_m_ values was taken (*n* = 3 × 3).

## CONFLICT OF INTEREST

The authors have no competing interests to declare that are relevant to the content of this article.

## Supporting information


**Figure S1.** Core phenylpropanoid pathway starting with L‐phenylalanine, leading to 4‐coumaroyl‐CoA via the enzymes phenylalanine ammonia‐lyase (PAL), cinnamate 4‐hydroxylase (C4H; CYP73), and 4‐coumarate CoA‐ligase (4CL). Changes introduced by the respective enzymes are marked in red.
**Figure S2.** Alignment (CLC Sequence Viewer 8) of all C4H candidate genes described in this work together with *Sorghum bicolor* SbC4H1 (GenBank AAK54447) and *Helianthus tuberosus* HtC4H1 (GenBank CAA78982). Conserved motifs are marked with black boxes, and variable or special amino acids are written in red.
**Figure S3.** Biosynthetic pathway leading to rosmarinic acid as an example of phenolic esters being hydroxylated by a tracheophyte CYP98 enzyme with C3H/C3′H activity. Changes introduced by the respective enzymes are marked in red.
**Figure S4.** Alignment of all CYP98 candidate genes (CLC Sequence Viewer 8) described in this work and *Ocimum basilicum* ObCYP98 (GenBank AAL99200.1). Conserved motifs are marked with black boxes, and variable or special amino acids are written in red.
**Figure S5.** AlphaFold 3 3D models of CYP73 enzymes (A MpC4H1, B MpC4H2) with two apparent corkscrew‐like transmembrane domains. The yellow (70 > plDDT > 50) and orange (plDDT < 50) parts of the models indicate a lower prediction confidence than the dark blue bodies.
**Figure S6.** Expression data of CYP73 and CYP98 in organs/tissues of *Anthoceros agrestis*, *Marchantia polymorpha* and *Physcomitrium patens*. Expression data were taken from PEATmoss (https://peatmoss.plantcode.cup.uni‐freiburg.de) for *A. agrestis* and *P. patens* and MarpolBase (https://mbex.marchantia.info) for *M. polymorpha*. A shaded color code is used to indicate expression levels: The darker the color, the higher the expression. Point of harvest for *M. polymorpha* is given below the organ or tissue; h – hours, d – days. Media used for the cultivation of *Physcomitirum patens* are abbreviated; BlqA – BCDA (ammonium) liquid; BslA, BCDA (ammonium) solid; Bsl, BCD solid.
**Figure S7.** Western blot analysis of all enzymes characterized in this work. The first lane shows the marker RotiMark® Tricolor. (A) Western blot of CYP proteins fused to the FLAG‐tag, expressed from a vector containing the bryophyte CYP candidate genes in MCS I, detected with anti‐FLAG antibodies coupled to alkaline phosphatase, from left to right: AaC4H1, AaC4H2, MpC4H1, MpC4H2, MpC4H3, PpC4H1, PpC4H2, PpC4H3, MpCYP98, PpCYP98. (B) Western blot of CsCPR protein fused to the MYC‐tag, expressed from a vector containing the CPR gene from *Coleus scutellarioides* in MCS II, detected with anti‐MYC antibodies coupled to alkaline phosphatase from the same strains as in (A). (C) Western blot of *Chara braunii* CYP proteins fused to the FLAG‐tag, expressed from a vector containing the *Chara* CYP candidate genes in MCS I, detected with anti‐FLAG antibodies coupled to alkaline phosphatase, from left to right: CbCYP_cand_1, CbCYP_cand_1:K376R, CbCYP_cand_1:Fus1‐10:K265R, CbCYP_cand_2. (D) Western blot of CsCPR protein fused to the MYC‐tag, expressed from a vector containing the CPR gene from *Coleus scutellarioides* in MCS II, detected with anti‐MYC antibodies coupled to alkaline phosphatase from the same strains as in (C). The arrows indicate the faint bands just above 70 kDa. (E) Expected mass of the expressed proteins with the respective tag attached and their location on the blot figures.
**Figure S8.** Determination of kinetic parameters for C4H by substrate saturation curves with *t*‐cinnamic acid (B–H) or 3‐coumaric acid (b–h) as substrates. (A, a) K_m_ values and activities. Selected Michaelis‐Menten graphs for AaC4H1 (B, b), AaC4H2 (C, c), MpC4H1 (D, d), MpC4H2 (E, e), MpC4H3 (F, f), PpC4H1 (G, g), PpC4H2 (H, h).
**Figure S9.** Time‐dependent formation of 4‐coumaric acid from *t*‐cinnamic acid in CYP73 assays. (A) Extracted ion chromatograms (EIC) showing the formation of 4‐coumaric acid by the enzymes indicated in the legend; the black line shows the external standard, and the gray dotted line indicates the peak corresponding to the mass of the product. (B to I) Time‐dependent increase of the product peak in reactions with AaC4H1 (B), AaC4H2 (C), MpC4H1 (D), MpC4H2 (E), MpC4H3 (F), PpC4H1 (G), PpC4H2 (H), and PpC4H3 (I). The gray dotted line represents the rising peak at the same retention time as the external standard depicted by the black lines; the legend indicates the incubation time in minutes.
**Figure S10.** Substrate conversion by (putative) CYP98 (see color code at the right side) shown by extracted ion chromatograms (EIC) of the LC‐MS analyses for all tested substrates (A–M). The Y‐axis shows the molecular formula of the negatively charged ion of the product. No standards were available for most products, but they are shown in black if available. The gray dotted line indicates the peak corresponding to the mass of the product. The following substrates were used: (A) *t*‐cinnamic acid, (B) 3‐phenylpropanoic acid, (C) 3‐coumaric acid, (D) *N*‐(4‐coumaroyl)‐anthranilic acid, E *N*‐(4‐coumaroyl)‐3′‐hydroxyanthranilic acid, F *N*‐(4‐coumaroyl)‐tyramine, (G) 4‐coumaroyl‐5′‐*O*‐shikimic acid, (H) 4‐coumaroyl‐3′‐*O*‐quinic acid, I 4‐coumaroyl‐5′‐*O*‐quinic acid, (J) 4‐coumaroyl‐4′‐hydroxyphenyllactic acid, (K) 4‐coumaroyl‐3′,4′‐dihydroxyphenyllactic acid, (L) caffeoyl‐4‐hydroxyphenyllactic acid, (M) 4‐coumaroyl‐4′‐*O*‐threonate.
**Figure S11.** CYP substrates with their respective expected hydroxylation products.
**Figure S12.** Time‐dependent hydroxylation of *N*‐(4‐coumaroyl)‐anthranilic acid (A), *N*‐(4‐coumaroyl)‐3′‐hydroxyanthranilic acid (B) and *N*‐(4‐coumaroyl)‐tyramine (C). Left: Extracted ion chromatograms (EIC) showing the product formation by the enzymes indicated in the legend; the gray dotted line indicates the peak corresponding to the mass of the product. Right: Time‐dependent increase of the product peak in reactions with MpCYP98. The gray dotted line indicates the peak corresponding to the mass of the product, the legend indicates the incubation time in minutes.
**Figure S13.** Determination of kinetic parameters for CYP98 by substrate saturation curves with *N*‐(4‐coumaroyl)‐anthranilic acid (B, b), *N*‐(4‐coumaroyl)‐3’‐hydroxyanthranilic acid (C, c) or *N*‐(4‐coumaroyl)‐tyramine (D; only for MpCYP98) as substrates. A K_m_ values and activities. Selected Michaelis‐Menten graphs for MpCYP98 (B, C, D) and PpCYP98 (b, c).
**Table S1.** CYP73 sequences (cinnamic acid 4‐hydroxylases, C4H) identified in this work with their amino acid sequences, lengths, and accession numbers, together with published C4H sequences from *Sorghum bicolor* (Zhang et al., 2020) and *Helianthus* *tuberosus* (Schoch et al., 2003). Light gray shading indicates parts of the protein inside the endoplasmic reticulum (ER), signal peptides are shaded turquoise, while transmembrane stretches are shaded red, as calculated by DeepTMHMM 1.0.57 on https://dtu.biolib.com. Unshaded parts of the protein are on the cytosolic side of the ER membrane. Small letters indicate expressed parts of the protein without function. Written in red are differences in the amino acid sequence compared to the native sequence. The enzymes characterized in this work and the signature motif for CYP73 AAIETT or variants thereof are written in bold. Aa, number of amino acid residues.
**Table S2.** (A) Pairwise sequence comparison (EMBOSS Needle) of all retrieved C4H amino acid sequences from *Physcomitrium patens*. (B) Small phylogenetic tree depicting the relationship of these sequences.
**Table S3.** (A) Pairwise sequence comparison (EMBOSS Needle) of all retrieved C4H amino acid sequences from *Marchantia polymorpha*. (B) Small phylogenetic tree depicting the relationship of these sequences.
**Table S4.** CYP98 sequences characterized in this work with their amino acid sequences, lengths, and accession numbers, together with CYP98 from *Ocimum basilicum* (Gang et al., 2002). Light gray shading indicates parts of the protein inside the endoplasmic reticulum (ER), signal peptides are shaded turquoise, while transmembrane stretches are shaded red, as calculated by DeepTMHMM 1.0.57 on https://dtu.biolib.com. Unshaded parts of the protein are on the cytosolic side of the ER membrane. The signature motif for CYP98 AGMDTT or variants thereof is written in bold. Aa = number of amino acid residues.
**Table S5.** Pairwise sequence comparison (EMBOSS Needle) of all retrieved amino acid sequences for CYP98 in *Anthoceros agrestis*, *Marchantia polymorpha* and *Physcomitrium patens*.
**Table S6.** CYP73 and CYP98 amino acid sequences used to construct the phylogenetic tree (Figure 1). CYP98 sequences are shaded in gray. Sequences characterized in this study are written in bold.
**Table S7.** List of primers used for PCR and sequencing. Underlined are the base pairs binding to the inserted gene, restriction sites are marked in bold letters.
**Table S8.** PCR setups for each method. Standard protocols for each PCR were used according to the manufacturer. The primer concentration was adjusted before adding 0.5 μl of each primer to the reaction.
**Table S9.** PCR programs for each method. Each protocol was preceded by an additional denaturation period at 95°C for 2 min and an additional elongation period of 8 min after the last cycle at the same temperature. The mutagenesis PCR used the CloneAmp™ HiFi PCR Premix (Takara Bio). Amplification for homologous recombination was performed with the AccuPrime™ *Taq* DNA polymerase standard protocol (Invitrogen), and Colony PCR used the GoTaq® G2 DNA polymerase standard protocol (Promega).
**Table S10.** Table of solvents and detection wavelengths used to analyze each product by HPLC.

## Data Availability

The data that support the findings of this study are available in the Supplementary Material of this article.
