# Supplementary material for: Cytochrome P450 enzymes in the phenylpropanoid pathway: CYP73 and CYP98 candidate genes in Chara braunii, Anthoceros agrestis, Marchantia polymorpha and Physcomitrium patens
Source: Plant J. 2026 Oct 7;128(1):e71152. doi: 10.1111/tpj.71152 (PMC13646711; doi:10.1111/tpj.71152)
Supplement: Supplementary file 1 [file TPJ-128-0-s001.pdf]

Supporting Material for

Cytochrome P450 enzymes in the phenylpropanoid pathway: CYP73 and CYP98 candidate genes in *Chara braunii*, *Anthoceros agrestis*, *Marchantia polymorpha* and *Physcomitrium patens*

Christoph E. G. Aggarwal-Kentrath and Maike Petersen

Supporting Figures

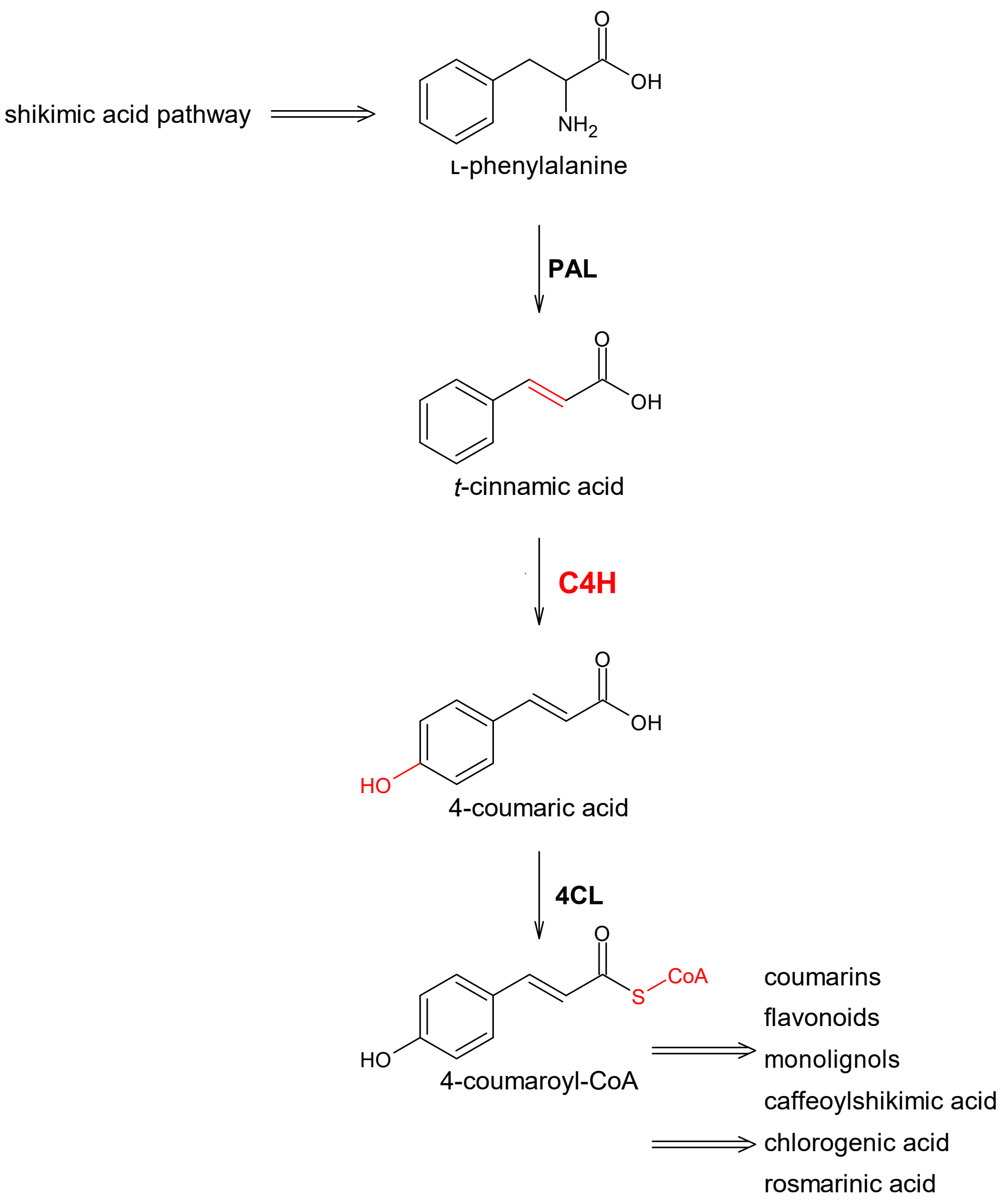

Figure S1 Core phenylpropanoid pathway starting with L-phenylalanine leading to 4-coumaroyl-CoA via the enzymes phenylalanine ammonia-lyase (PAL), cinnamate 4-hydroxylase (C4H; CYP73) and 4-coumarate CoA-ligase (4CL). Changes introduced by the respective enzymes are marked in red

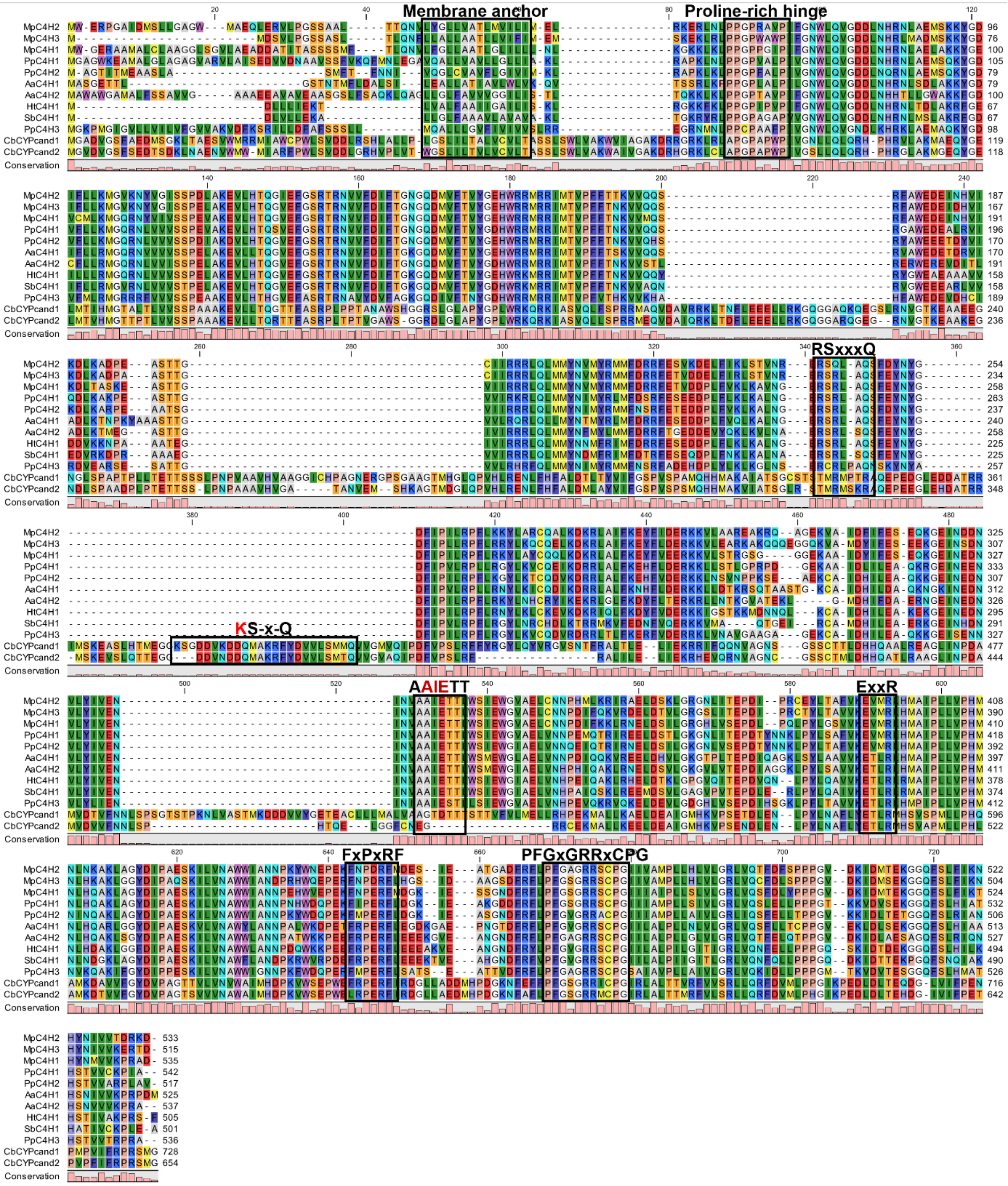

Figure S2 Alignment (CLC Sequence Viewer 8) of all C4H candidate genes described in this work together with *Sorghum bicolor* SbC4H1 (GenBank AAK54447) and *Helianthus tuberosus* HtC4H1 (GenBank CAA78982). Conserved motifs are marked with black boxes, and variable or special amino acids are written in red

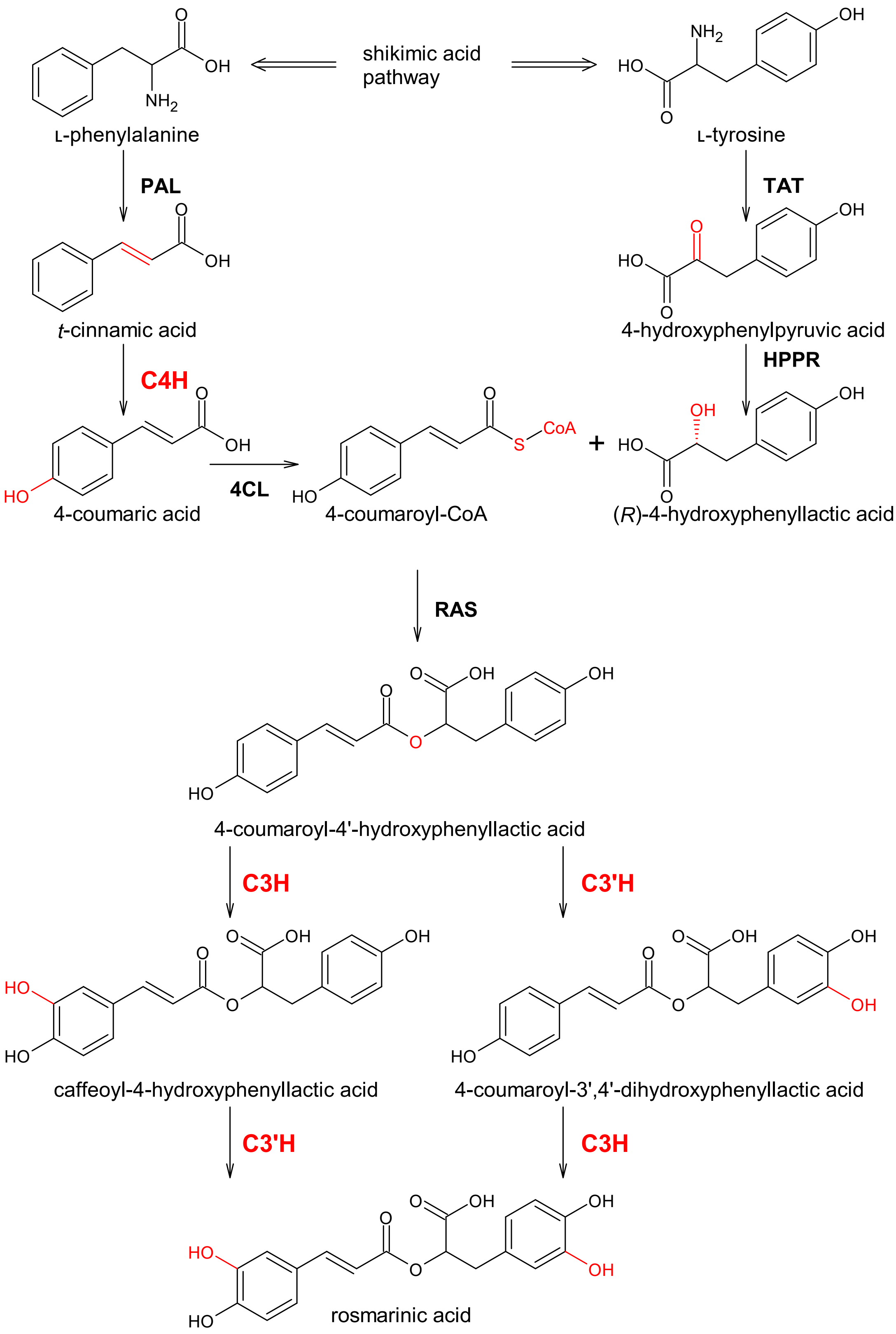

Figure S3 Biosynthetic pathway leading to rosmarinic acid as an example of phenolic esters being hydroxylated by a tracheophyte CYP98 enzymes with C3H/C3'H activity. Changes introduced by the respective enzymes are marked in red

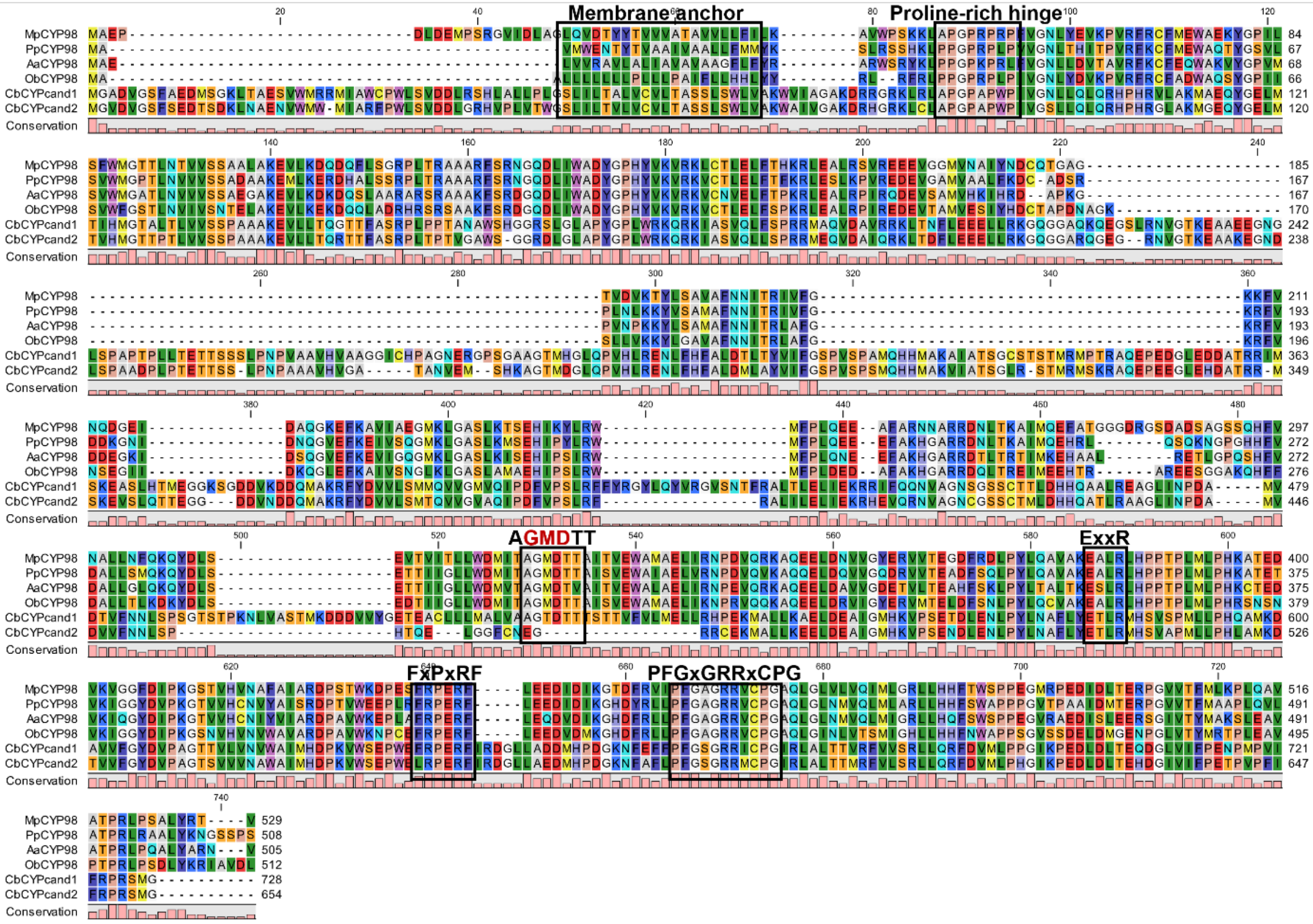

Figure S4 Alignment of all CYP98 candidate genes (CLC Sequence Viewer 8) described in this work and *Ocimum basilicum* ObCYP98 (GenBank AAL99200.1). Conserved motifs are marked with black boxes, and variable or special amino acids are written in red

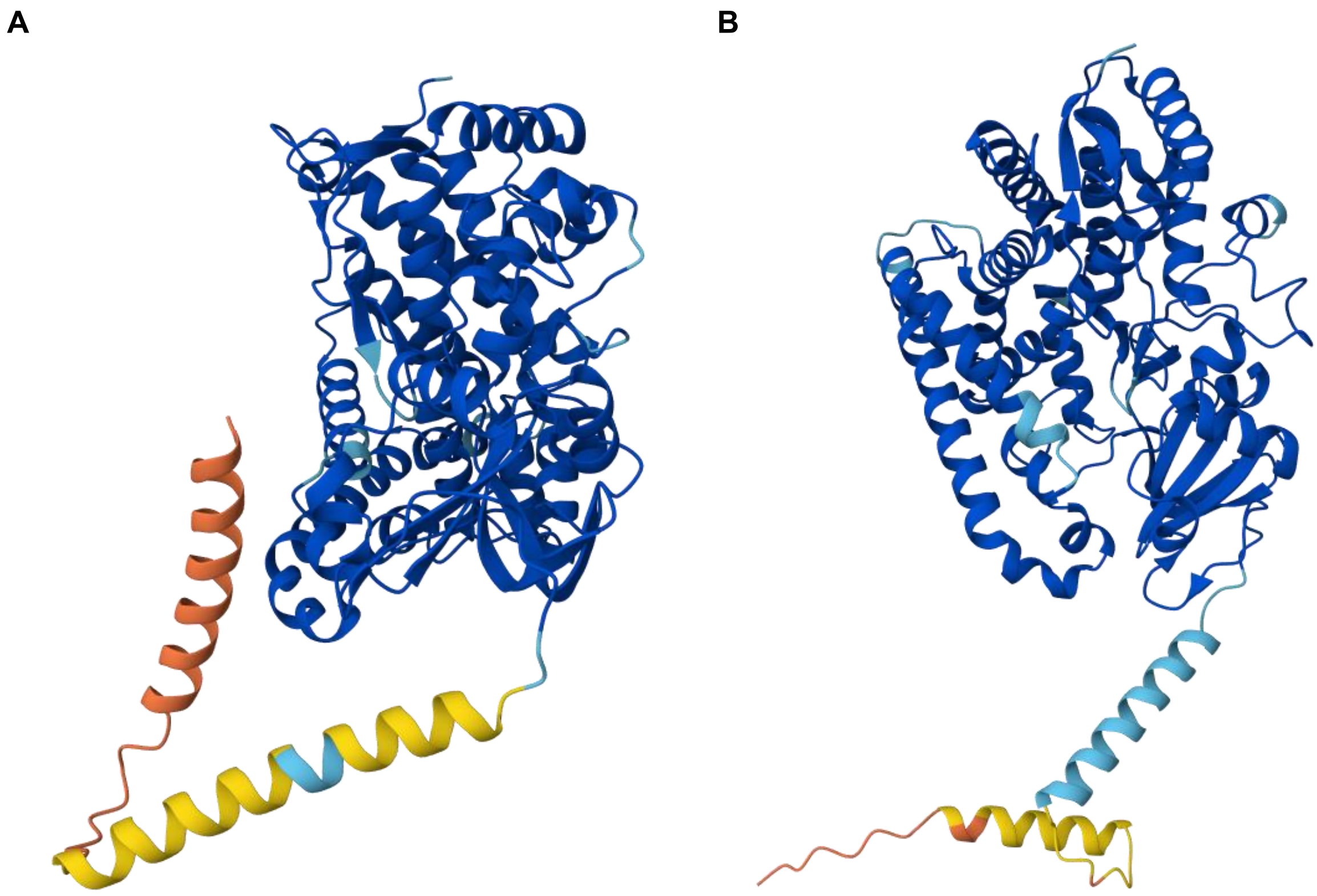

Figure S5 AlphaFold 3 3D models of CYP73 enzymes (**A** MpC4H1, **B** MpC4H2) with two apparent corkscrew-like transmembrane domains. The yellow (70 > plDDT > 50) and orange (plDDT < 50) parts of the models indicate a lower prediction confidence than the dark blue bodies

Organism	Organ or tissue					
Anthoceros agrestis	Gametophyte	Foot parenchym	Meristem	Columella	Sporogenous tissue	Assimilative tissue
PEATmoss	-	-	-	-	-	-
AaC4H1	180.86	2116.70	2316.64	2497.18	2343.68	2531.99
AaC4H2	5534.80	1569.05	1399.05	2252.15	1145.96	1381.66
AaCYP98	1775.92	2167.26	2441.46	2772.21	1420.98	2432.79
Marchantia polymorpha	Sporeling	Thallus	Antheridiophore (*) Antheridium (#)	Archegoniophore (*) Archegonia (#)	Gemma cup (*) midrib (#)	Sporophyte (*) apical cell (#)
MarpolBase	0 (*) / 72 (#) h	9 (*) / 14 (#) d	Tak-1 mature	Tak-2 mature	21 (*) d / 21 (#) d	13 (*) / 9 (#) d
MpC4H1	* 65.34	* 193.76	* 192.71	* 219.79	* 121.17	* 66.67
	# 76.17	# 78.27	# 23.24	# 248.97	# 108.43	# 114.29
MpC4H2	* 0.43	* 20.06	* 2.96	* 5.91	* 13.95	* 1.72
	# 33.06	# 17.31	# 0.20	# 13.66	# 13.53	# 0.66
MpC4H3	* 3.96	* 28.67	* 5.38	* 10.53	* 6.70	* 3.44
	# 3.50	# 8.26	# 1.02	# 5.40	# 5.36	# 0.95
MpCYP98	* 46.77	* 55.86	* 95.87	* 142.92	* 44.00	* 40.59
	# 28.64	# 48.83	# 14.60	# 43.37	# 17.75	# 20.29
Physcomitrium patens	Dry Spores	Imbibed spores	Germinating spores	Protonema	Adult gametophores	Protoplast
PEATmoss - Gransden WT	-	BlqA	BslA	Bsl	Bsl	BslA
PpC4H1	3.25	2.53	25.66	24.23	90.90	26.84
PpC4H2	2.56	4.58	46.11	45.08	28.54	72.13
PpC4H3	0.23	0.05	0.25	0.71	0.08	12.32
PpCYP98	0.20	0.00	19.75	8.34	27.57	6.31

Figure S6 Expression data of CYP73 and CYP98 in organs/tissues of *Anthoceros agrestis*, *Marchantia polymorpha* and *Physcomitrium patens*. Expression data was taken from PEATmoss (https://peatmoss.plantcode.cup.uni-freiburg.de) for *A. agrestis* and *P. patens* and MarpolBase (https://mbex.marchantia.info) for *M. polymorpha*. A shaded color code is used to indicate expression levels: The darker the color, the higher the expression. Point of harvest for M. polymorpha is given below the organ or tissue; h - hours, d - days. Media used for the cultivation of *Physocmitirum patens* are abbreviated; BlqA - BCDA (ammonium) liquid; BslA - BCDA (ammonium) solid; Bsl - BCD solid

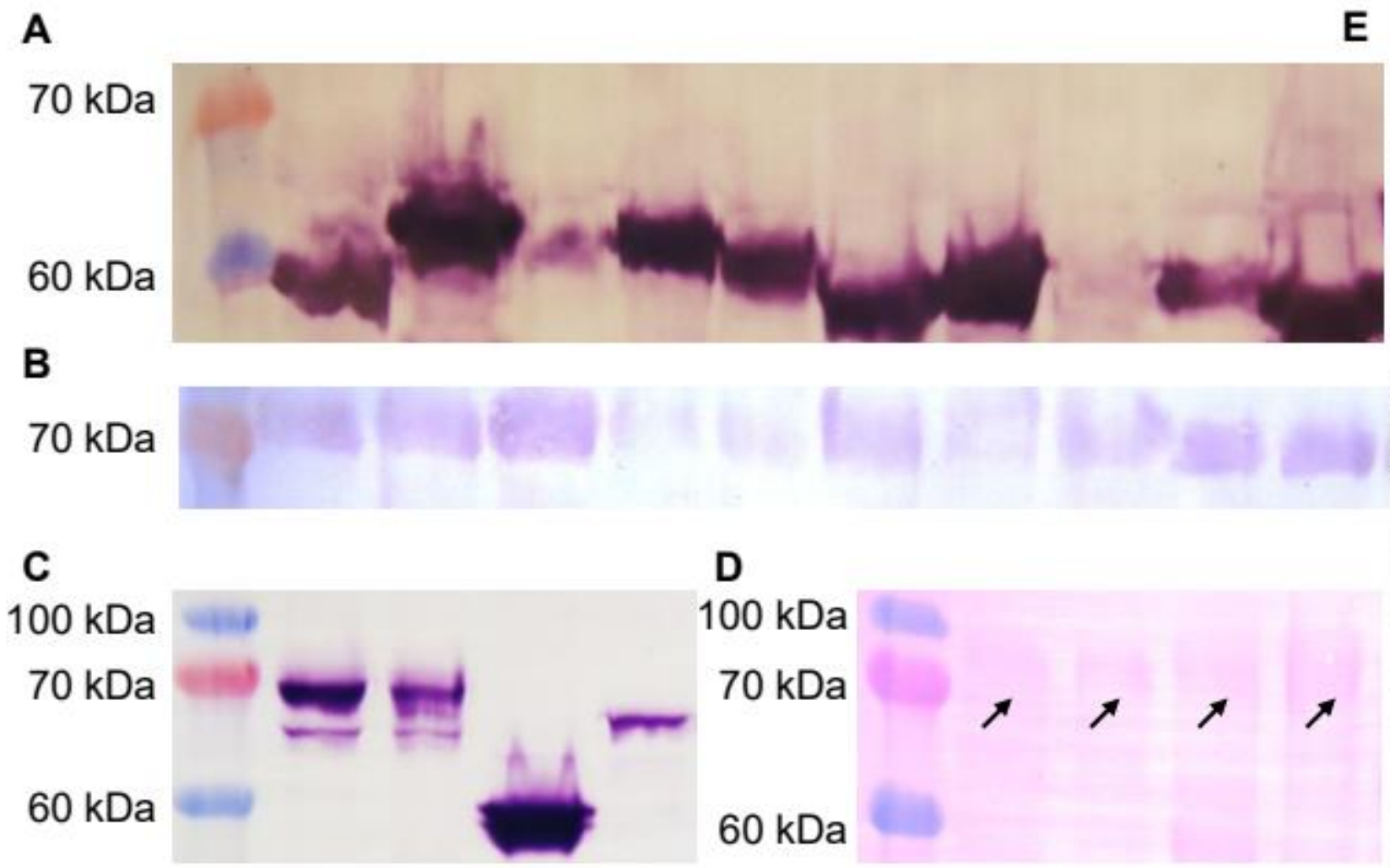

E

Enzyme	Figure	Lane	mass [kDa]
AaC4H1	A	1	60.58
AaC4H2	A	2	62.25
MpC4H1	A	3	62.27
MpC4H2	A	4	62.98
MpC4H3	A	5	61.09
PpC4H1	A	6	62.23
PpC4H2	A	7	60.50
PpC4H3	A	8	62.04
MpCYP98	A	9	60.77
PpCYP98	A	10	58.78
Cb$_{cand}$1	C	1	81.52
Cb$_{cand}$1:K376R	C	2	81.52
Cb$_{cand}$1: Fus1-10:K265R	C	3	64.02
Cb$_{cand}$2	C	4	74.02
CsCPR	B + D	All	79.69

Figure S7 Western blot analysis of all enzymes characterized in this work. The first lane shows the marker RotiMark® Tricolor. **A** Western blot of CYP proteins fused to the FLAG-tag expressed from a vector containing the bryophyte CYP candidate genes in MCS I detected with anti-FLAG antibodies coupled to alkaline phosphatase, from left to right: AaC4H1, AaC4H2, MpC4H1, MpC4H2, MpC4H3, PpC4H1, PpC4H2, PpC4H3, MpCYP98, PpCYP98. **B** Western blot of CsCPR protein fused to the MYC-tag expressed from a vector containing the CPR gene from *Coleus scutellarioides* in MCS II detected with anti-MYC antibodies coupled to alkaline phosphatase from the same strains as in **A**. **C** Western blot of *Chara braunii* CYP proteins fused to the FLAG-tag expressed from a vector containing the *Chara* CYP candidate genes in MCS I detected with anti-FLAG antibodies coupled to alkaline phosphatase, from left to right: CbCYP$_{cand}$1, CbCYP$_{cand}$1:K376R, CbCYP$_{cand}$1:Fus1-10:K265R, CbCYP$_{cand}$2. **D** Western blot of CsCPR protein fused to the MYC-tag expressed from a vector containing the CPR gene from *Coleus scutellarioides* in MCS II detected with anti-MYC antibodies coupled to alkaline phosphatase from the same strains as in **C.** The arrows indicate the faint bands just above 70 kDa. **E** Expected mass of the expressed proteins with the respective tag attached and their location on the blot figures

A

Enzyme \ Substrate	*t*-cinnamic acid	
	K_m [µM]	activity [fkat]
AaC4H1	43.95 ± 15.47	8.03 ± 2.44
AaC4H2	1.10 ± 0.31	139.97 ± 32.92
MpC4H1	13.70 ± 3.04	19.24 ± 14.79
MpC4H2	10.51 ± 1.58	59.87 ± 47.50
MpC4H3	11.70 ± 2.00	27.51 ± 0.90
PpC4H1	75.72 ± 16.27	243.13 ± 90.58
PpC4H2	17.24 ± 4.72	90.69 ± 51.72

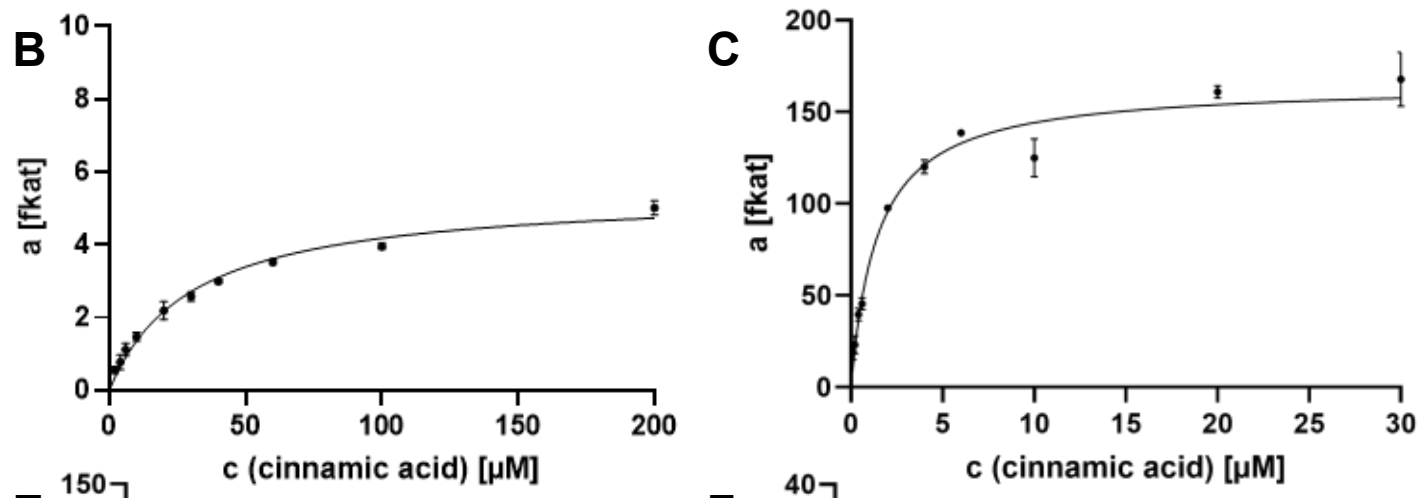

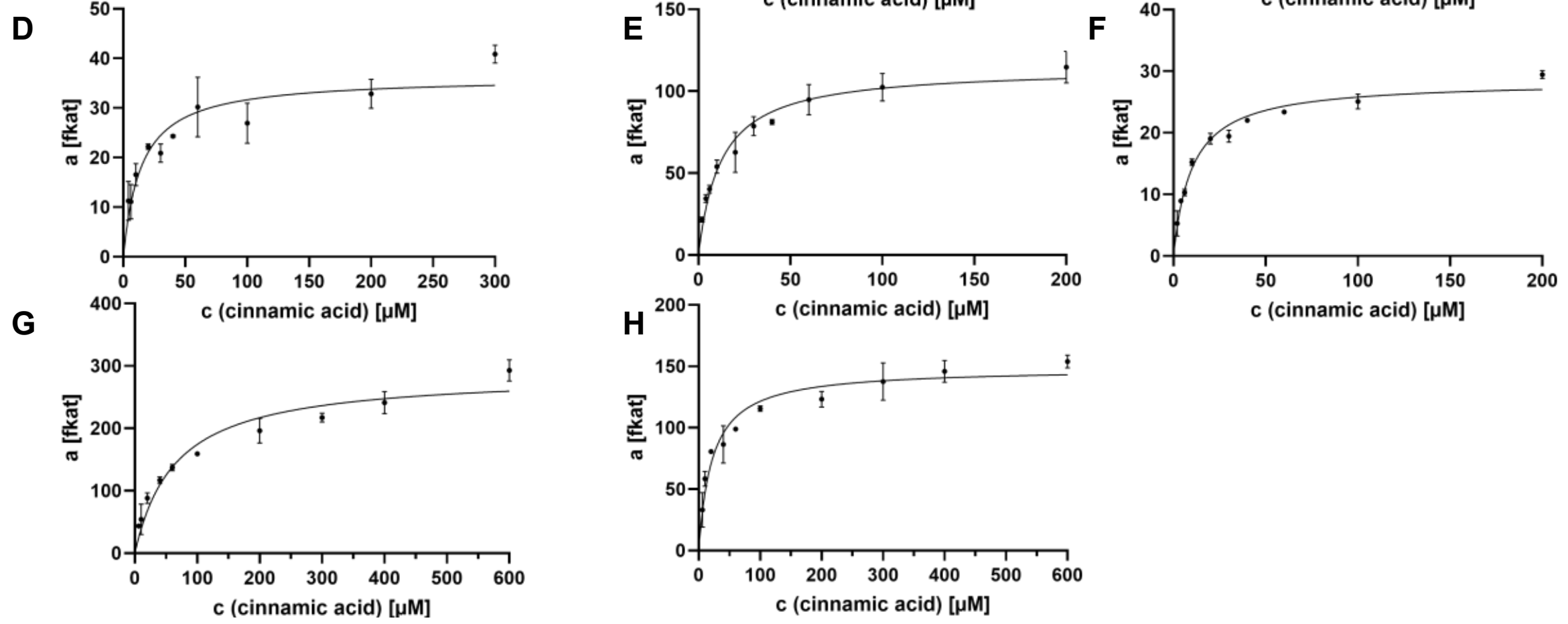

a

Enzyme \ Substrate	3-coumaric acid	
	K_m [µM]	activity [fkat]
AaC4H1	346.33 ± 151.90	54.44 ± 24.93
AaC4H2	40.14 ± 8.09	281.73 ± 36.93
MpC4H1	125.63 ± 8.16	26.68 ± 6.36
MpC4H2	245.87 ± 48.52	180.97 ± 11.44
MpC4H3	202.53 ± 8.74	51.92 ± 4.95
PpC4H1	299.53 ± 33.20	138.93 ± 56.42
PpC4H2	130.52 ± 51.26	271.4 ± 95.03

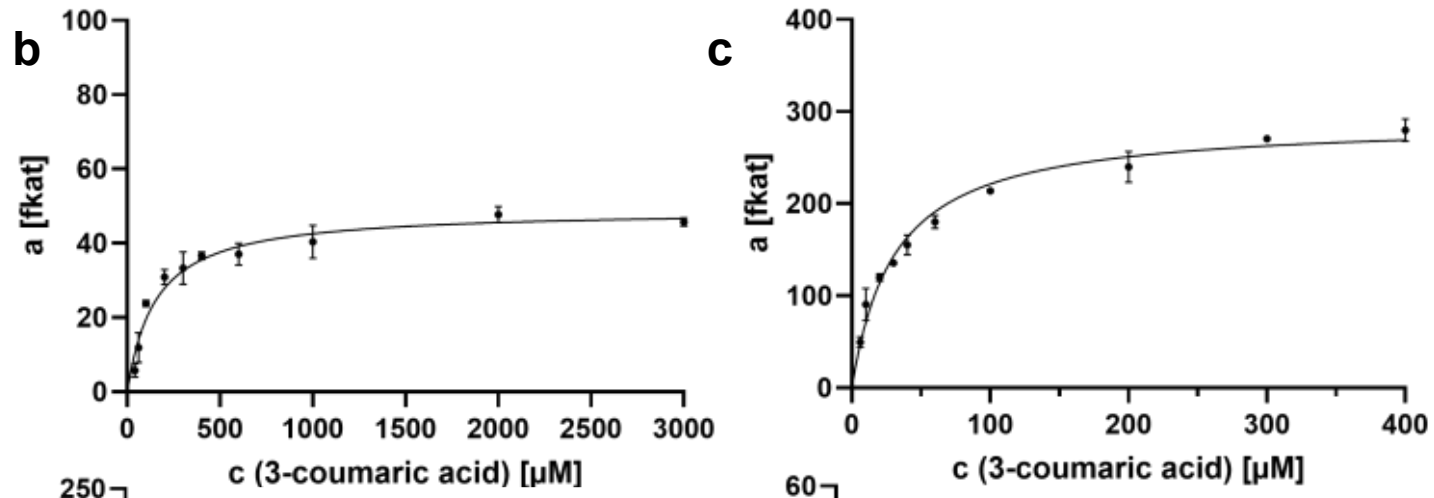

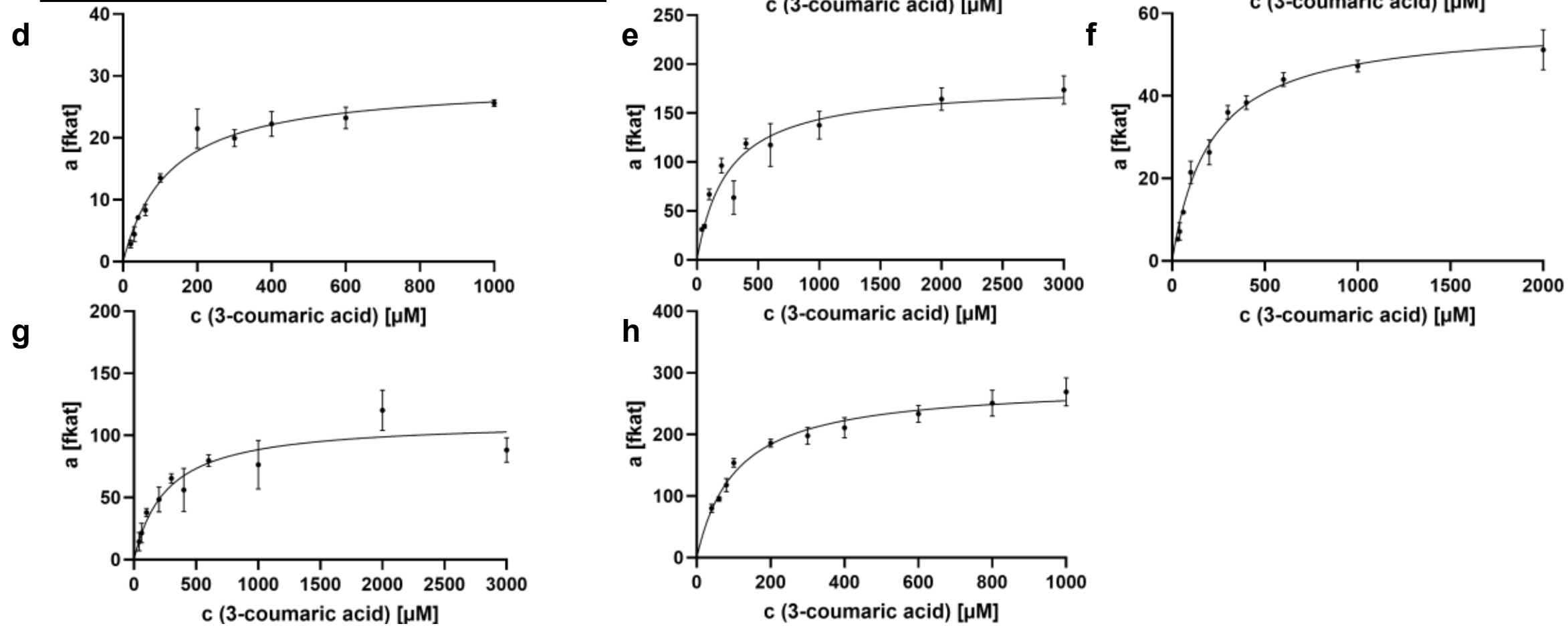

Figure S8 Determination of kinetic parameters for C4H by substrate saturation curves with *t*-cinnamic acid (**B**-**H**) or 3-coumaric acid (**b**-**h**) as substrates. **A, a** K_m values and activities. Selected Michaelis-Menten graphs for AaC4H1 (**B**, **b**), AaC4H2 (**C**, **c**), MpC4H1 (**D**, **d**), MpC4H2 (**E**, **e**), MpC4H3 (**F**, **f**), PpC4H1 (**G**, **g**), PpC4H2 (**H**, **h**)

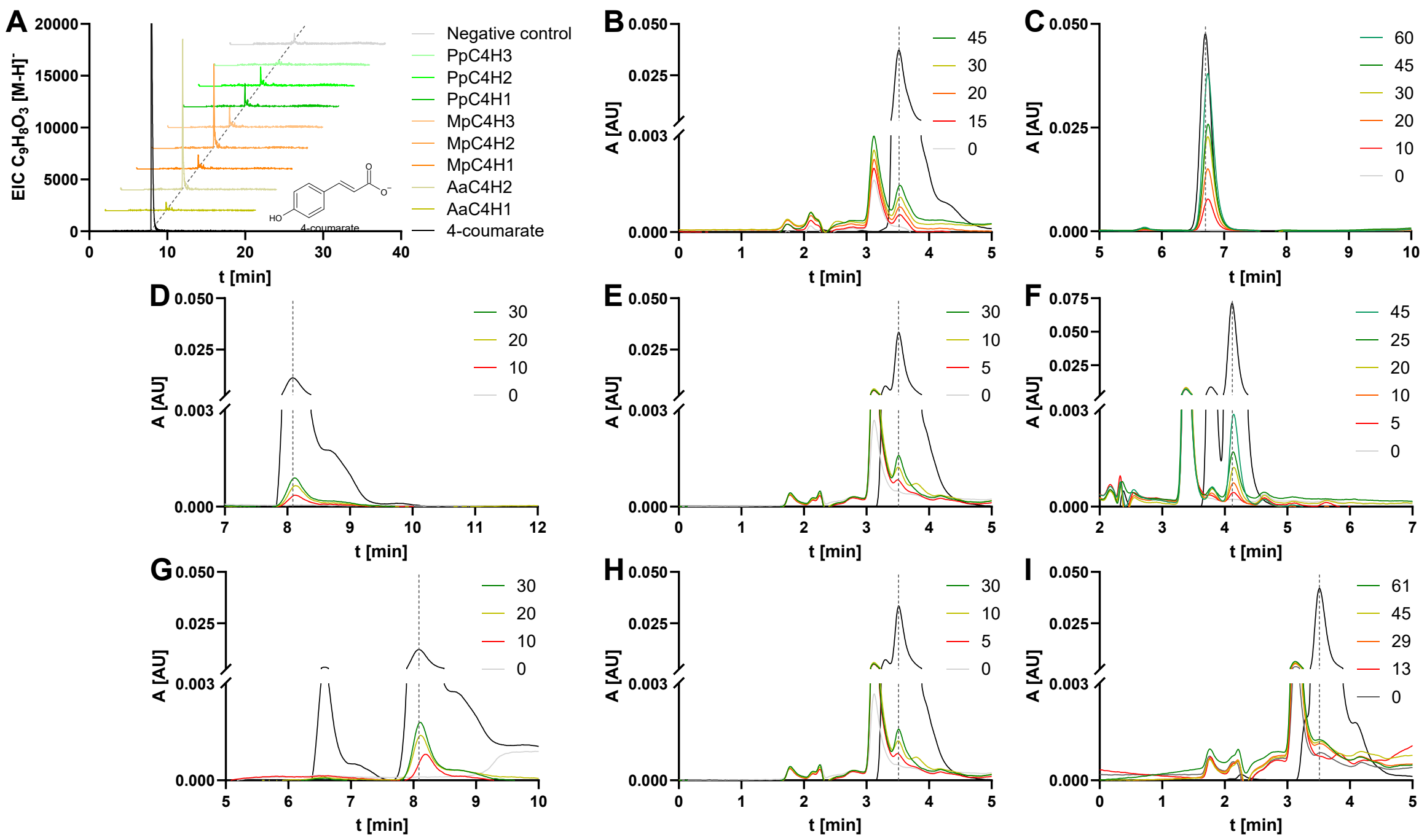

Figure S9 Time-dependent formation of 4-coumaric acid from *t*-cinnamic acid in CYP73 assays. **A** Extracted ion chromatograms (EIC) showing the formation of 4-coumaric acid by the enzymes indicated in the legend; the black line shows the external standard and the gray dotted line indicates the peak corresponding to the mass of the product. **B** to **I** Time-dependent increase of the product peak in reactions with AaC4H1 (**B**), AaC4H2 (**C**), MpC4H1 (**D**), MpC4H2 (**E**), MpC4H3 (**F**), PpC4H1 (**G**), PpC4H2 (**H**) and PpC4H3 (**I**). The grey dotted line represents the rising peak of the same retention time as the external standard depicted by the black lines; the legend indicates the incubation time in minutes

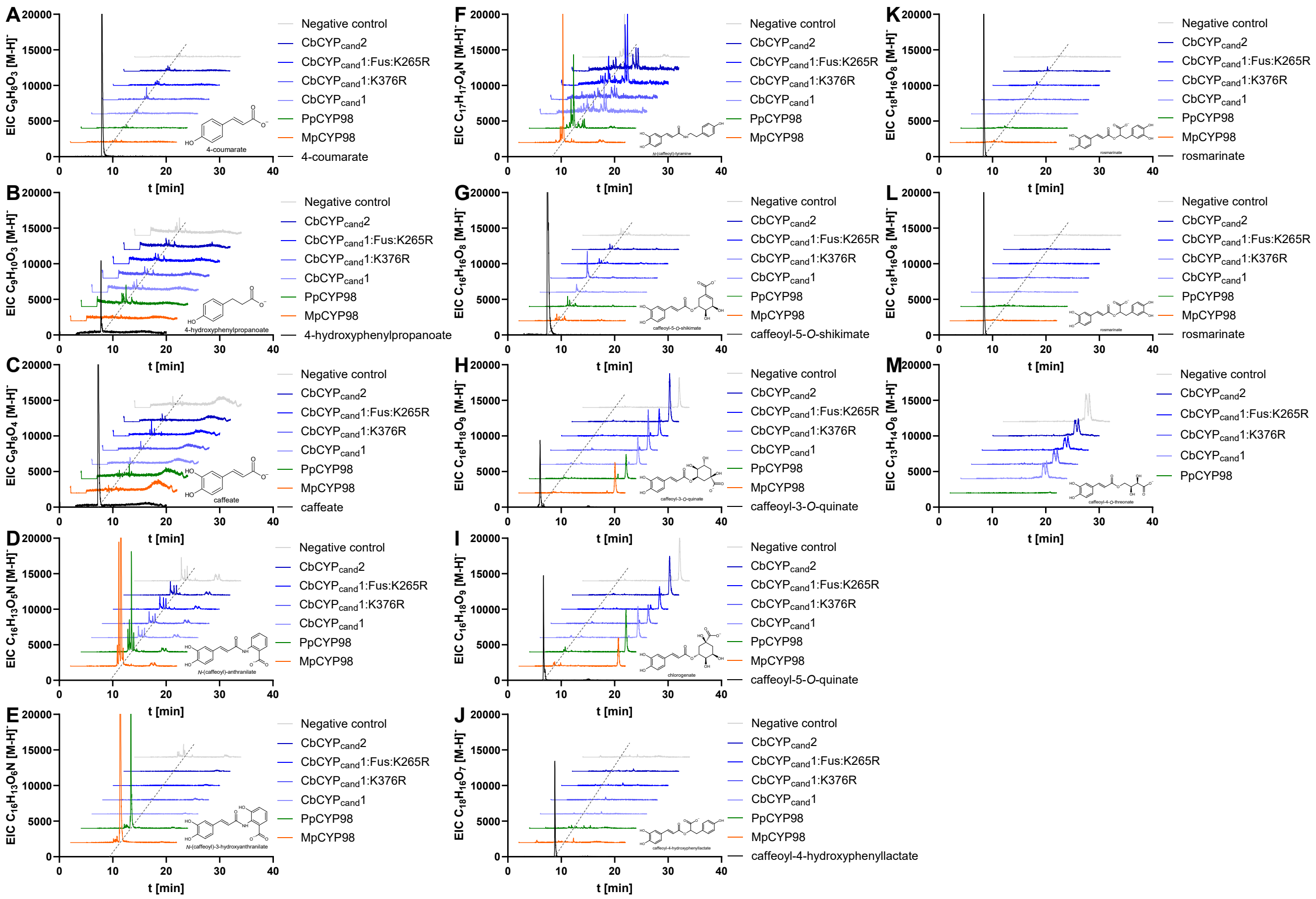

Figure S10 Substrate conversion by (putative) CYP98 (see color code at the right side) shown by extracted ion chromatograms (EIC) of the LC-MS analyses for all tested substrates (**A**-**M**). The Y-axis shows the molecular formular of the negatively charged ion of the product. No standards were available for most products but are shown in black if available. The gray dotted line indicates the peak corresponding to the mass of the product. The following substrates were used: **A** *t*-cinnamic acid, **B** 3-phenylpropanoic acid, **C** 3-coumaric acid, **D** *N*-(4-coumaroyl)-anthranilic acid, **E** *N*-(4-coumaroyl)-3'-hydroxyanthranilic acid, **F** *N*-(4-coumaroyl)-tyramine, **G** 4-coumaroyl-5'-*O*-shikimic acid, **H** 4-coumaroyl-3'-*O*-quinic acid, **I** 4-coumaroyl-5'-*O*-quinic acid, **J** 4-coumaroyl-4'-hydroxyphenyllactic acid, **K** 4-coumaroyl-3',4'-dihydroxyphenyllactic acid, **L** caffeoyl-4-hydroxyphenyllactic acid, **M** 4-coumaroyl-4'-*O*-threonate

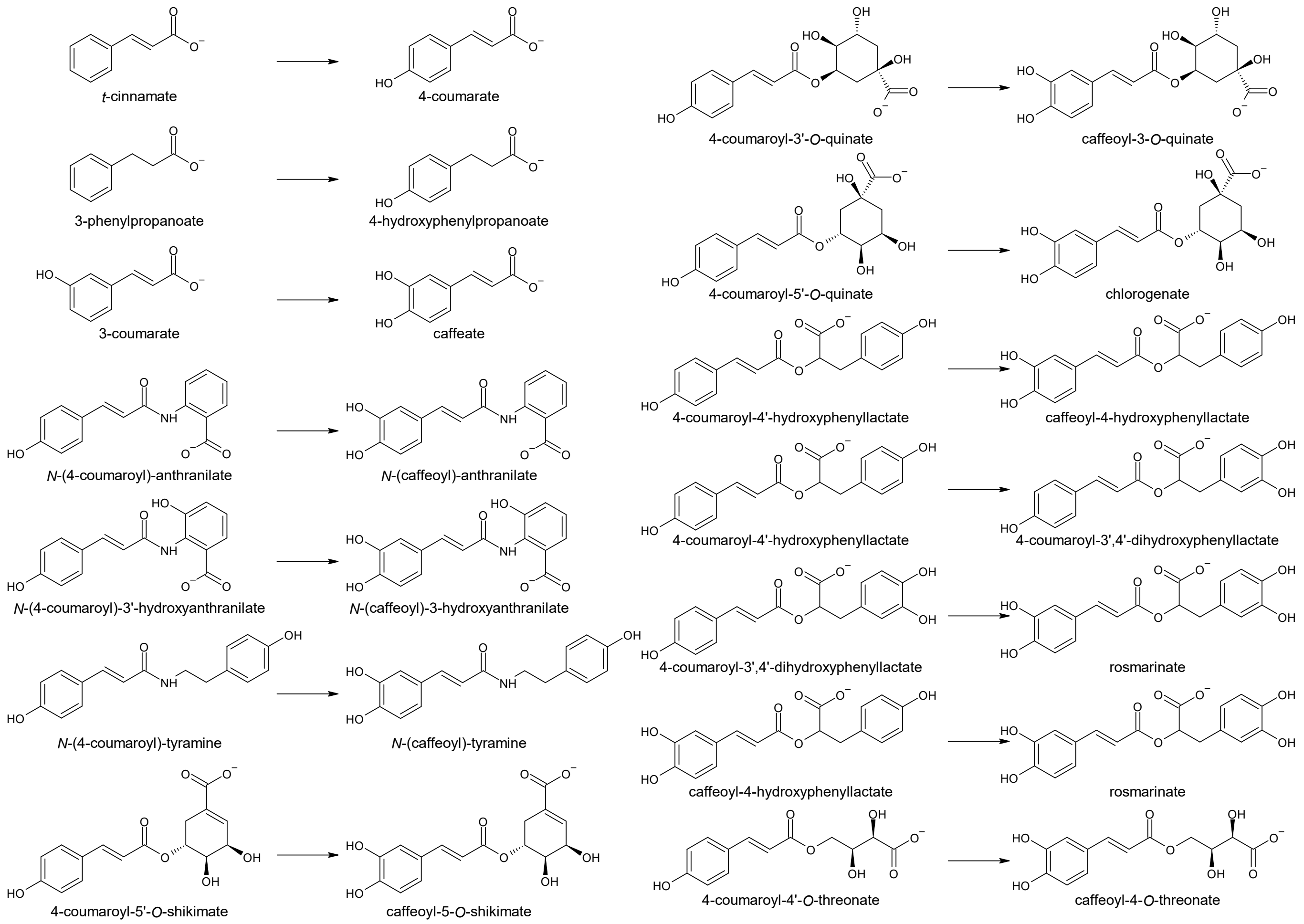

Figure S11 CYP substrates with their respective expected hydroxylation products

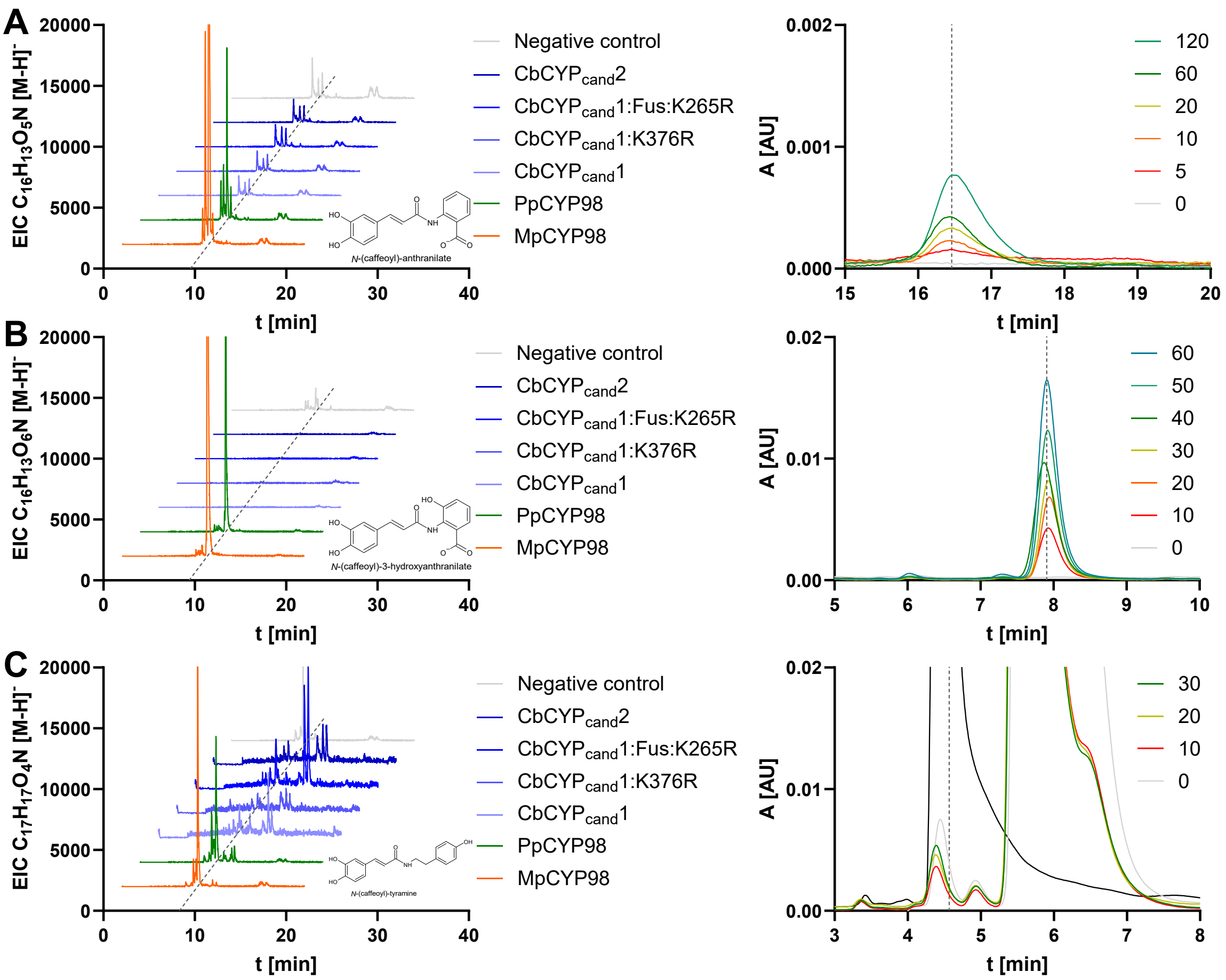

Figure S12 Time-dependent hydroxylation of *N*-(4-coumaroyl)-anthranilic acid (**A**), *N*-(4-coumaroyl)-3'-hydroxyanthranilic acid (**B**) and *N*-(4-coumaroyl)-tyramine (**C**). Left: Extracted ion chromatograms (EIC) showing the product formation by the enzymes indicated in the legend; the gray dotted line indicates the peak corresponding to the mass of the product. Right: Time-dependent increase of the product peak in reactions with MpCYP98. The gray dotted line indicates the peak corresponding to the mass of the product, the legend indicates the incubation time in minutes

A

Substrate \ Enzyme	MpCYP98		PpCYP98	
	K_m [µM]	activity [fkat]	K_m [µM]	activity [fkat]
N-(4-coumaroyl)-anthranilate	22.19 ± 3.22	327.20 ± 68.42	83.47 ± 5.74	86.94 ± 18.20
N-(4-coumaroyl)-3’-hydroxyanthranilate	30.43 ± 6.04	600.20 ± 79.76	24.30 ± 2.33	57.08 ± 7.05
N-(4-coumaroyl)-tyramine	31.16 ± 9.03	84.93 ± 21.27	not quantifiable	not quantifiable

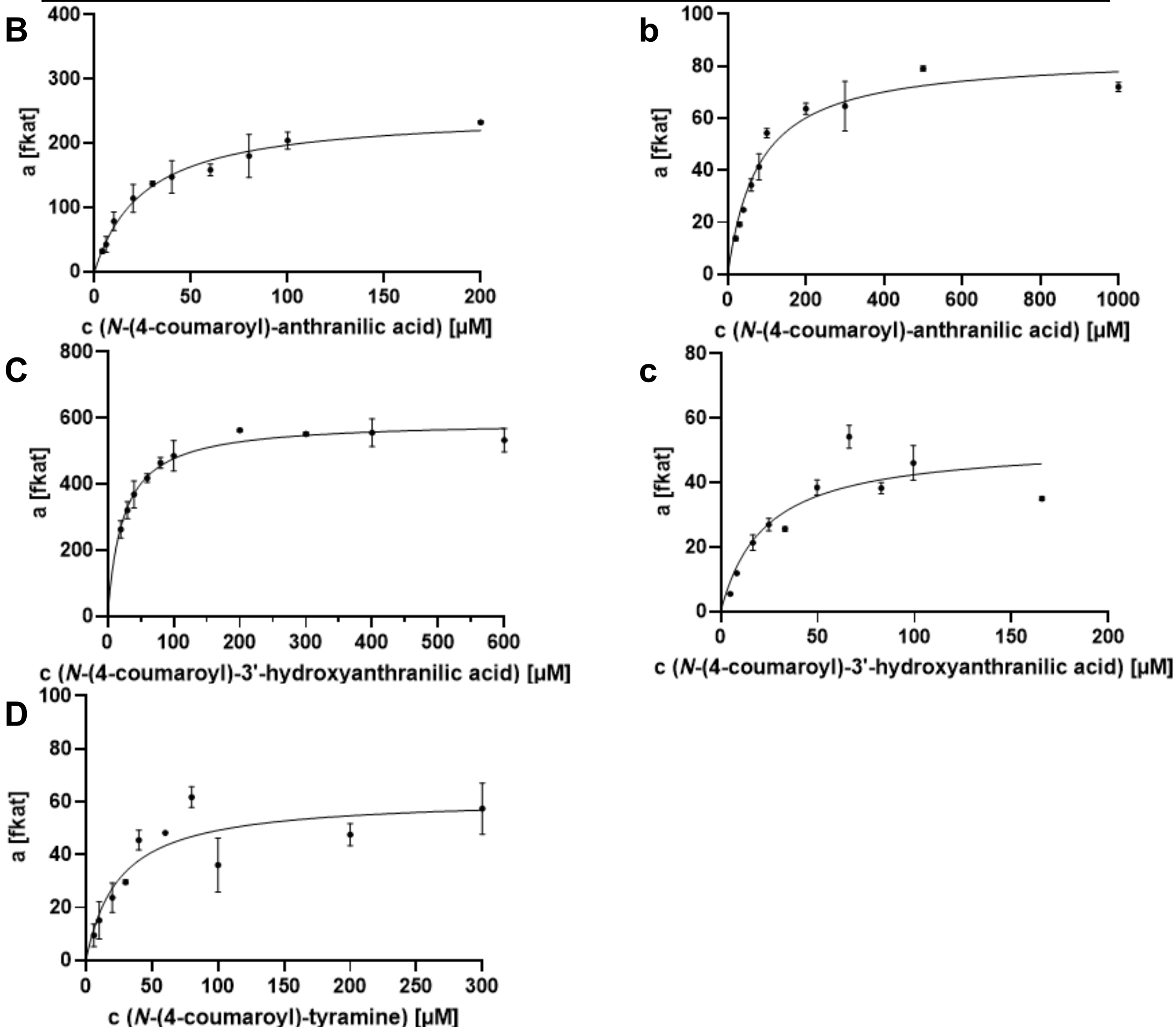

Figure S13 Determination of kinetic parameters for CYP98 by substrate saturation curves with *N*-(4-coumaroyl)-anthranilic acid (**B**, **b**), *N*-(4-coumaroyl)-3’-hydroxyanthranilic acid (**C**, **c**) or *N*-(4-coumaroyl)-tyramine (**D**; only for MpCYP98) as substrates. **A** K_m values and activities. Selected Michaelis-Menten graphs for MpCYP98 (**B**, **C**, **D**) and PpCYP98 (**b**, **c**)

Supporting Tables

Table S1 CYP73 sequences (cinnamic acid 4-hydroxylases, C4H) identified in this work with their amino acid sequences, lengths and accession numbers together with published C4H sequences from *Sorghum bicolor* (Zhang *et al.*, 2020) and *Helianthus* tuberosus (Schoch *et al.*, 2003). Light grey shading indicates parts of the protein inside the endoplasmic reticulum (ER), signal peptides are shaded turquoise, while transmembrane stretches are shaded red as calculated by DeepTMHMM 1.0.57 on https://dtu.biolib.com. Unshaded parts of the protein are on the cytosolic side of the ER membrane. Small letters indicate expressed parts of the protein without function. Written in red are differences in the amino acid sequence compared to the native sequence. The enzymes characterized in this work and the signature motif for CYP73 **AAIETT** or variants thereof are written in **bold**. Aa = number of amino acid residues

Name in this work	Amino acid sequence	Aa	Accession numbers and details
Chara braunii			
$CbCYP_{cand}1$	MGADVGSFAEDMSGKLTAESVWMRRMIAWCPWLSVDDLRSHL<br>ALLPLGSLILTALVCVLTASSLSWLVAKWVIAGAKDRRGRKLRLA<br>PGPAPWPIVGNLLQLQRHPHRVLAKMAEQYGELMTIHMGTALTL<br>VVSSPAAAKEVLLTQGTTFASRPLPPTANAWSHGGRSLGLAPY<br>GPLWRKQRKIASVQLFSPRRMAQVDAVRRKLTNFLEEELLRKG<br>QGGAQKQEGSLRNVGTKEAAEEGNGLSPAPTPLLTETTSSSLP<br>NPVAAVHVAAGGICHPAGNERGPSGAAGTMHGLQPVHLRENLF<br>HFALDTLTYVIFGSPVSPAMQHHMAKAIATSGCSTSTMRMPTRA<br>QEPEDGLEDDATRRIMSKEASLHTMEGGKSGDDVKDDQMAKR<br>FYDVVLSMMQVVGMVQIPDFVPSLRFFYRGYLQYVRGVSNTFR<br>ALTLELIEKRRIFQQNVAGNSGSSCTTLDHHQAALREAGLINPDA<br>MVDTVFNNLSPSGTSTPKNLVASTMKDDDVVYGETEACLLLMAL<br>VAAGTDTTTSTTVFVLMELLRHPEKMALLKAELDEAIGMHKVPSE<br>TDLENLPYLNAFLYETLRMHSVSPMLLPHQAMKDAVVFGYDVPA<br>GTTVLVNVWAIMHDPKVWSEPWEFRPERFIRDGLLADDMHPDG<br>KNFEFFPFGSGRRICPGIRLALTTVRFVVSRLLQRFDVMLPPGIK<br>PEDLDLTEQDGLVIFPENPMPVIFRPRSMG	728	Phycocosm: jgi_Chabra1_325637_rna-CBR_g12414<br>Nucleotide sequence in GenBank: BFEA01000014.1<br>UniProt: A0A388JS94<br>No signal peptide or transmembrane part identified by DeepTMHMM
$CbCYP_{cand}1$:K376R	MGADVGSFAEDMSGKLTAESVWMRRMIAWCPWLSVDDLRSHL<br>ALLPLGSLILTALVCVLTASSLSWLVAKWVIAGAKDRRGRKLRLA<br>PGPAPWPIVGNLLQLQRHPHRVLAKMAEQYGELMTIHMGTALTL<br>VVSSPAAAKEVLLTQGTTFASRPLPPTANAWSHGGRSLGLAPY<br>GPLWRKQRKIASVQLFSPRRMAQVDAVRRKLTNFLEEELLRKG<br>QGGAQKQEGSLRNVGTKEAAEEGNGLSPAPTPLLTETTSSSLP<br>NPVAAVHVAAGGICHPAGNERGPSGAAGTMHGLQPVHLRENLF<br>HFALDTLTYVIFGSPVSPAMQHHMAKAIATSGCSTSTMRMPTRA<br>QEPEDGLEDDATRRIMSKEASLHTMEGGRSGDDVKDDQMAKR<br>FYDVVLSMMQVVGMVQIPDFVPSLRFFYRGYLQYVRGVSNTFR<br>ALTLELIEKRRIFQQNVAGNSGSSCTTLDHHQAALREAGLINPDA<br>MVDTVFNNLSPSGTSTPKNLVASTMKDDDVVYGETEACLLLMAL<br>VAAGTDTTTSTTVFVLMELLRHPEKMALLKAELDEAIGMHKVPSE<br>TDLENLPYLNAFLYETLRMHSVSPMLLPHQAMKDAVVFGYDVPA<br>GTTVLVNVWAIMHDPKVWSEPWEFRPERFIRDGLLADDMHPDG<br>KNFEFFPFGSGRRICPGIRLALTTVRFVVSRLLQRFDVMLPPGIK<br>PEDLDLTEQDGLVIFPENPMPVIFRPRSMG	728	No signal peptide or transmembrane part identified by DeepTMHMM

Name in this work	Amino acid sequence	Aa	Accession numbers and details
CbCYP$_{cand}$1:Fus 1-10:K265R	MGADVGSFAEDMSGKLTAESVWMRRMIAWCPWLSVDDLRSHL<br>ALLPLGSLILTALVCVLTASSLSWLVAKWVIAGAKDRRGRKLRLA<br>PGPAPWPIVGNLLQLQRHPHRVLAKMAEQYGELMTIHMGTALTL<br>VVSSPAAAKEVLLTQGTTFASRPLPPTANAWSHGGRSLGLAPY<br>GPLWRKQRKIASVQLFSPRRMAQVDAVRRKLTNGSLRNVGTKE<br>AAGTMHGLLRENLFHFALDTLTYVIFGSRAQEPEDGLTRRLHTM<br>EGGRSSMMQVVGMVQIPDFVPSLRFFYRGYLQYVRGVSNTFRA<br>LTLELIEKRRIFQQNVAGNSGSSCTTLDHHQAALREAGLIKDDDV<br>VYLLMALVAAGTDTTTSTTVFVLMELLRHPEKMALLKAELDEAIG<br>MHKVPSETDLENLPYLNAFLYETLRMHSVSPMLLPHQAMKDAVV<br>FGYDVPAGTTVLVNVWAIMHDPKVWSEPWEFRPERFIRDGLMH<br>PDGKNFEFFPFGSGRRICPGIRLALTTVRFVVSRLLQRFDVMLPP<br>GIKPEDLDLTEQDGLVIFPENPMPVIFRPRSMG	559	No signal peptide or transmembrane part identified by DeepTMHMM
CbCYP$_{cand}$2	MGVDVGSFSEDTSDKLNAENVWMWMIARFPWLSVDDLGRHVP<br>LVTWGSLILTVLVCVLTASSLSWLVAKWAIVGAKDRHGRKLCLAP<br>GPAPWPIVGSLLQLQRHPHRGLAKMGEQYGELMTVHMGTTPTL<br>VVSSPAAAKEVLLTQRTTFASRPLTPTVGAWSGGRDLGLAPYGP<br>LWRKQRKIASVQLLSPRRMEQVDAIQRKLTDFLEEELLRKGQGG<br>ARQGEGRNVGTKEAAKEGNDLSPAADPLPTETTSSLPNPAAAV<br>HVGATANVEMSHKAGTMDGLQPVHLRENLFHFALDMLAYVIFG<br>SPVSPSMQHHMAKVIATSGLRSTMRMSKRAQEPEEGLEHDATR<br>RMSKEVSLQTTEGGDDVNDDQMAKRFYDVVLSMTQVVGVAQIP<br>DFVPSLRFRALILELIEKRHEVQRNVAGNCGSSCTMLDHHQATL<br>RAAGLINPDAMVDVVFNNLSPHTQELGGFCNEGRRCEKMALLK<br>EELDEAIGMHKVPSENDLENLPYLNAFLYETLRMHSVAPMLLPHL<br>AMKDTVVFGYDVPAGTSVVVNAWAIMHDPKVWSEPWELRPER<br>FIRDGLLAEDMHPDGKNFAFLPFGSGRRMCPGIRLALTTMRFVL<br>SRLLQRFDVMLPHGIKPEDLDLTEHDGIVIFPETPVPFIFRPRSMG	654	Phycocosm: jgi_Chabra1_325651_rna-CBR_g12428<br>Nucleotide sequence in GenBank: BFEA01000014.1<br>UniProt: A0A388JSJ4
Anthoceros agrestis			
**AaC4H1**	masgettlgstntMFLDALSILEALLATIAVLWLVKQVTSSRLKPPPGPL<br>ALPILGNWLQVGDDLNHRNLSDLAKKYGDIFLLRMGQRNVVVVS<br>SPELAKEVLLTQGVEFGSRTRNVVFDIFTGKGQDMVFTVYGEH<br>WRRMRRIMTVPFFTSKVVQQSRVAWEDETDRVIADLKTNPKYA<br>AASTTGVVLRQRLQLLMYNTMYRLMFDRRFESEDDPLFVQLKAL<br>NGERSRLAQSFEYNYGDFIPVLRPLLRNYLKICQDIKDRRLALFK<br>NHFLDERKKLLDTKRSQTAASTGKCAIDHILDAQKNGKINEDNVL<br>YIVENINV**AAIETT**LWSMEWGLAELVNNPDIQQKVREELDHVLGK<br>GTPLTEPDIQAGKLSYLAAVVKETLRLHMAIPLLVPHMNLHQARL<br>GGYDIPAESKVLVNAWYLANNPALWKDPETFRPERFLEGDKGA<br>EPNGTDFRFLPFGVGRRSCPGIILALPLLNLVLGRLVQSFLLTCPP<br>GVEKLDLSEKGGQFSLHIAAHSNIVVKPRPDM	527	1kP: TWUW scaffold 2050103 (partial), BSNI scaffold 2050646 (partial)<br>University of Zurich: AagrBONN evm.model.Sc2ySwM 228.5028.1<br>Nucleotide sequence in GenBank: MK778366.1<br>UniProt: A0A6M3BD98
**AaC4H2**	MAWAWGAMALFSSAVVGAAAEEAVAVEAASGSLFSAQKLQAG<br>LLGLFAVVVGGILISTLTQKKLKLPPGPTAVPIFGNWLQVGDDLN<br>HHTLLGWAKKFGDCFLLRMGQRNLVVISSPELAKEVLHTQGVEF<br>GSRTRNVVFDIFTGNGQDMVFTVYGDHWRRMRRIMTVPFFTNK<br>VVSTLRERWEREVDITLADLKTMEGASTTGIVIRRRLQLMMYNF<br>MYLMMFDRRFTGEDDEVYQKLKVLNAERSRLSQSFEYNYGDFI<br>PILRPFLRKYLNHCRYIKEKRLGLFKDYFLTERKRLLNTKGVATEK<br>LGMDHIFDAERNGEINEDNVLYIVENINV**AAIETT**LWSMEWGIAEL<br>VNHPHIQAKLRNELDSVLGKGVLVTEPDIAGGKLPYLSAVVKETL<br>RLHMAIPLLVPHMNLHQAKLSGYDIPAESKILVNAWWLANNPAT<br>WKKPEEFRPERFLEEEKGVEANGNDFRFLPFGAGRRSCPGIILA<br>LPLLGLVLGRLVQTFELQTPPGVDKIDLAESAGQFSLRIQNHSNV<br>VVKPRA	537	1kP: TWUW scaffold 2050390 (partial), BSNI scaffold 2050769 (partial)<br>University of Zurich: AagrBONN evm.TU.Sc2ySwM_344.41 28 (part of scaffold)
Marchantia polymorpha			
Mapoly0163s0018<br>**MpC4H1**	MWGERAAMALCLAAGGLSGVLAEADDATITASSSSMFTLQNVLF<br>GLLAATLGLILLLNLKGKKLKLPPGPPGIPIFGNWLQIGDDLNHRN<br>LAELAKKYGEVCMLKMGQRNYVIVSSPEVAKEVLHTQGVEFGS<br>RTRNVVFDIFTGNGQDMVFTVYGEHWRRMRRIMTVPFFTNKVV<br>MQSRFAWEDEINHVIKDLTASKEASTTGVIIRRRLQLMMYNVMY<br>RMMFDRRFETVDDPLFVKLKAVNGERSRLAQSFEYNYGDFIPIL<br>RPFLRKYLAYCQQLKDKRLALFKEYFVEERKKVLSTRGSGGGEK<br>AAIDYIFESEQKGEINEDNVLYIVENINV**AAIETT**LWSIEWGVAELC<br>NNPDIFKKLRNELDSILGRGHLVSEPDLPQLPYLGSVVKEVMRLH<br>MAIPLLVPHMNLHQAKLAGYDIPAESKILVNAWWIANNPEHWVE<br>PEKFNPERFMDGKIESSGNDFRFLPFGSGRRSCPGIIIAIPLLSLV<br>LGRLVQSFDLYPPPGVDKIDMTEKGGQFSLFIKTHYNMVVKPRA<br>D	535	Phytozome: Mapoly0163s0018<br>Marpolbase: Mp6g00020.1<br>Nucleotide sequence in GenBank: KZ772833.1<br>UniProt: A0A2R6W3M3

Name in this work	Amino acid sequence	Aa	Accession numbers and details
Mapoly0081s0008<br><br>**MpC4H2**	MWERPGAIDMSLLGAGWMAEQLERVLPGSSAALTTQNVLYGLL VATLMVILMELRKERLNLPPGPRAVPIFGNWLQVGDDLNHRNLA EMSKKYGDIFLLKMGVKNYVGISSPDLAKEVLHTQGIEFGSRTRN VVFDIFTGNGQDMVFTVYGEHWRRMRRIMTVPFFTTKVVQQSR FAWEDEINHVIKDLKADPEASTTGCIIRRRLQLMMYNVMYRMMF DRRFESVKDELFIKLSTVNRERSQLAQSFDYNYGDFIPILRPFLKK YLARCQALKDKRLAIFKEYFIDERKKVLAAREAKRQAGEKVAIDFI FESEQKGEINDDNVLYIVENINV**AAIETT**LWSIEWGVAELCNNPH MLKRIRAELDSKLGRGNLITEPDIPRCEYLTAFVKEVMRLHMAIPL LVPHMNLNKAKLAGYDIPAESKILVNAWWIANNPKYWNEPEKFN PDRFMDESIEATGADFRFLPFGAGRRSCPGIIVAMPLLHLVLGRL VQTFDFSPPPGVDKIDMTEKGGQFSLFIKNHYNIVVTDRKD	533	Phytozome: Mapoly0081s0008<br><br>Marpolbase: Mp8g05070.1<br><br>Nucleotide sequence in GenBank: KZ772753.1<br><br>UniProt: A0A2R6WK84
Mapoly0081s0009<br><br>**MpC4H3**	MMDSVLPGSSASLTLQNFLLALLAATLLVIFIMEMSKEKLRLPPGP WAWPIFGNWLQVGDDLNHRLMADMSKKYGDIFLLKMGVKNYV GISSPELAKEVLHTQGVEFGSRTRNVVFDIFTGNGQDMVFTVYG EHWRRMRRIMTVPFFTNKVVQQSRFAWEDEIDHVIKDLKADPAA STTGCIIRRRLQLMMYNVMYRMMFDRRFETVDDELFIRLSTVNR ERSRLAQSFEYNYGDFIPLLRPFLRRYLKQCQELKDKRLAIFKEY FLDERKKVLEARKAKQQQEGGQKVAMDYIFESEEKGEINSDNVL YIVENINV**AAIETT**LWSIEWGVAELCNNPDIFQKVRDELDTVLGR GSLITEPDIPRCTYLTAVVKEVMRLHMAIPLLVPHMNLHKAKLGG YDIPAQSKILVNAWWIANDPRHWQEPERFNPDRFLHGSIDAAGS DFRFLPFGSGRRSCPGIIVAMPLLHLVLGRLVQCFDLSPPPGVDK IDMSEKGGQFSLFIKNHYNIVVKERTD	515	Phytozome: Mapoly0081s0009<br><br>Marpolbase: Mp8g05080.1<br><br>Nucleotide sequence in GenBank: KZ772753.1<br><br>UniProt: A0A2R6WK95<br><br>No signal peptide or transmembrane part identified by DeepTMHMM
Mapoly0129s0029	MEAMVNWESSWLSYLVAAITTVVAILLYFRLSHWQESRADRERN LPPAPYYYPLVGNLPSLVGDFPFKSLAKLSWNNRWPIFRLKLSG HAGPSTYVISSLELAHEVLKSSGLEFACRNVGVNFAFRMLSQDA RGAPRGVSGNTLRGTWRNARRVMVNGLVQTRKVFGDALLVRD LEQSFRALEADGHADGHYVHVLLPHIAQYLVNHTLHFVYGYQGE NSMGQQINAHNETVVMCLTKAQQCLFFPILQPFLQPQYTKVMEQ TLQRKYQLHETLLSQREIIRAKQGNSSWYFCDTCLEAEKTGEITH DDALHIIDEIFL**AGVMTT**AATIEWGLGELVNQQGIIKKLQQEMDAM IGVGVPLRANDIDRLPYLKATIKEIFRMHPAVPLLVPHMNPEDAKL GSYDIPKESLAMVNVWKLQRDPELWHEPNVFNPDRFLEHPEIDV CGMDNRLLPFGAGRRMCPGLSVGLLNTQMWIGRLVQRYHITLP QGVQNLDLTPKFGASVVIKNGLRVCLKRRQ	517	Phytozome: Mapoly0129s0029<br><br>Marpolbase: Mp2g10040<br><br>Nucleotide sequence in GenBank: KZ772801.1<br><br>UniProt: A0A2R6W8E7<br><br>No signal peptide or transmembrane part identified by DeepTMHMM
Mapoly0084s0023	MAATNASTLLGHMYDPRSWSFGTTSLLVAVVVAVSMGLLKVRV KRKTSNLPPGPRSYPLIGHMLEMRGAEMPEKLWSLSQTNNWPI MSFIFPNIPTVIVSSPEVTKEMLITKGNVFNSRVREMRATRGLRM SPDGKSETGALFTPHGPAWKVTRKIMVAGQNEFKRILGHDYVVR VLLPQLYKEIDSEVSIDARHQIAKTMIVRILMVFVFGPGAEQYAEEI IPLNDQLFVVTDNNHAIETFLPCIGESASWLYNKLVARRLVARKR KAYEEILSQRGVKYNLKNEWSLSDMVQEAERNGEINHDQALEIY EECFF**AGQDPT**VAAVEGTLRMIVENPKILVKVQQEMDEVLGEGK QLRLTDVERLPYFQGVIKEGLRLHRAIPFLIPHCAHEDATLGGFDI PKNMMLLVNLWGLARDPASFPDPYEAKPERFLERDTNFSGNDG KYVVFGVGQRICPGQSMALFILNMVIGSMCQRYNLAVAKDWKH ERDTGTFFKEVALHINLTRRQGFTP	513	Phytozome: Mapoly0084s0023<br><br>Marpolbase: Mp5g17730<br><br>Nucleotide sequence in GenBank: KZ772756.1<br><br>UniProt: A0A2R6WJC8<br><br>No signal peptide or transmembrane part identified by DeepTMHMM
Mapoly0041s0103	MTTIVSSAQDLWTSWTLTTQICVGVAVVLFSIVVMKSSRKIKYKL PPGLPQLPVIGNMLDMRGGEMHEKWWQIAQEKKWPIMSFKFSS KLMLIVSTPEVTKEMLITKGNVFNSRDKDTFLNLALRKSPDGKSE TGALFTPHGPAWKVTRKIMVAGQNEFKKNVGDQYIVDTILPQLY SEIAGETSIDARIRVAKMTVIRILLVFVFGPGAEQYADQIVPLNDDL FHVVDTNNAIEFFLPLVGRQMSWLYYKIYQRGLVARKYKLYDSIL SQRGIKYNQAKQWSLSDMVQLAEKNGEINHYQALQIFEECFFGG QDTAGGAIEGILKMLVENQEVLVKIQKEMDDVVGVGKQFRHTDA DRLPYFQAAIKEGLRLHRAIPVLTPHVAHEDTTLGGYDIPKDTIITV NLWGLARDPASFPDPFEVKPERFLGKDTNFSGNDCKYVVFGVG QRICPGQTLALFMVNVLVGSLAQRYHFELGNDWKHKRDAGTFF KELALHLKLTKRHGFQ	507	Phytozome: Mapoly0041s0103<br><br>Marpolbase:<br><br>Mp4g18220<br><br>Nucleotide sequence in GenBank: KZ772713.1<br><br>UniProt: A0A2R6X2A9<br><br>No signal peptide or transmembrane part identified by DeepTMHMM

Name in this work	Amino acid sequence	Aa	Accession numbers and details
Mapoly0010s0184	MENATFNSLKDSLVNQAWPDMWKPVLALVLLGIVSMKLFGGKS DGRRYPPGPKPLPFIGNMNLLFGDVLPKKLAEIAAQNKWPVMSF KLVNKQKVYVVSSPELTKEVLVTQGQTFASRSPPTFAQRYMVY GKDGKDTGVLFTHYSPTWRTNRKLMTVGQLQHRKIHGDAFIVDL LQVLYNELETKKGALSRNGEHPFEVRTLICRVFLLRIFLVFMFGP GAEKYGEELNALNDIMFYCLDMSEFAKWFFPHIEPLFDLYWGRY VTRKCMADKFRILKTILEDRSRYGGEKKWSFCDLLADSEKNGEIS TDGSMHLLDEVIF**AGQETT**AKAIEGAIKELADRPDILKKIQAEMDE KIGKKQLRLEDVEKLPYFQAFIKECLRLHRTLTILVPHVNMEDTKL DGYDIPKGSSIWINVWAMAHNPEIFPDPFVLKPERFLNKEVGLAG EDPSYIVFGAGRRICPGSPLAMNCTHMIVGSLAQRYDIKFPSDW TKDKKTVFGFIPKERALEVCFTKREGF	517	Phytozome: Mapoly0010s0184<br>Marpolbase: Mp5g22720<br>Nucleotide sequence in GenBank: KZ772682.1<br>UniProt: A0A2R6XL27<br>No signal peptide or transmembrane part identified by DeepTMHMM
Mapoly0084s0010	MENASFESIKDSLVSQSWPEMWKAILAMVVVAVVTVKVLGGKR DDRKYPPGPKPLPIIGNMNLLFGDVLPKKLAELAVANNWPIMTFK LVNKQKVFVVSSPEVTKEVLVTQGQVFASRSPPTFAQRYMVYG EDGKDVGVLFTHYSQTWRTNRKLMTMGQAQHRKIHGDAFIVDL LQVLYKEIETGKGALSRNGEQPFEIRTMVCRVFLLRIFLVFMFGP GAEKYGEELNALNDTMFYCLDMSEFAKWFFPMFEPIFDLYWGRI VTRRCMNSKFHILEQIFDDRAKYGGEKSWSFCDILRDAEKEGEIS ANNAMHLLDEVIF**AGQETT**AKAIEGAFKELAQRPDILKKIQEEMD RVIGKKPFRLDDVEKLPYFQAFIKECLRLHRTLTILVPHMNMEDV KLGGYDIPKGSSIWINVWAMAHDPKVFPDPFVLKPERFLNWDVG LAGEDPKYIVFGAGRRMCPGTPLAMNCTHMIVGSLAQRYDMRF PTDWTKDKVTKFGFIPKERALEICFTRRAGF	517	Phytozome: Mapoly0084s0010<br>Marpolbase: Mp5g17580<br>Nucleotide sequence in GenBank: KZ772756.1<br>UniProt: A0A2R6WJ74<br>No signal peptide or transmembrane part identified by DeepTMHMM
Physcomitrium patens			
Pp3c25_10190<br>**PpC4H1**	mgagwkeamalglagagvarvlaisedvvdnaavssfvkqfMNLEGAVQALLVA VLLGLLIAKLRAPKLNLPPGPVALPIVGNWLQVGDDLNHRNLAEM SQKYGDVFLLKMGQRNLVVVSSPEVAKEVLHTQSVEFGSRTRN VVFDIFTGNGQDMVFTVYGDHWRRMRRIMTVPFFTNKVVQQSR GAWEDEALRVIQDLKAKPEASTTGVVIRKRLQLMMYNIMYRLMF DSRFESEEDPLFLKLKALNGERSRLAQSFEYNYGDFIPVLRPLLR GYLKVCQEIKDRRLALFKEHFLDERKKLLSTLGPRPDGEKAAIDLI LEAQKRGEINEENVLYIVENINV**AAIETT**LWSIEWGVAELVNNPE MQTRIREELDSTLGKGNLITEPDTYNNKLPYLSAFVKEVMRLHMA IPLLVPHMNLHQAKLAGYDIPAESKILVNAWWIANNPNHWDQPE KFIPERFLDGKIEAKGDDFRFLPFGSGRRSCPGIIIAMPLLSIVLGR LVQSLELLPPPGTKKVDVSEKGGQFSLHIATHSTVVCKPIA	542	Phytozome: Pp3c25_10190, Phypa_140533<br>Nucleotide sequence in GenBank: ABEU02000025.1<br>UniProt: A0A2K1IEE9
Pp3c4_21680<br>**PpC4H2**	magtitMEAASLASMFTFNNIVQGLCVAVFLGIVIMKLRAPKLKLPP GPFALPIVGNWLQVGDDLNQRNLAEMSQKYGDVFLLKMGQRNL VVVSSPDIAKDVLHTQGVEFGSRTRNVVFDIFTGNGQDMVFTVY GEHWRRMRRIMTVPFFTNKVVQHSRYAWEEETDYVIKDLKARP EAATSGVIIRRRLQLMMYNIMYRMMFNSRFETEDDPLFVKLKALN GERSRLAQSFDYNYGDFIPILRPFLKGYLKTCQDVKDRRLALFKE HFVDERKKLNSVNPPKSEAEKCAIDHILEAQKKGEINEDNVLYIVE NINV**AAIETT**LWSIEWGIAELVNNQEIQTRIRNELDSILGKGNLVSE PDTYNNKLPYLTAFVKEVMRLHMAIPLLVPHMNINQAKLAGYDIP AESKILVNAWWIANNPKYWDQPEKFMPERFLDGKIEASGNDFRF LPFGVGRRACPGIIAMPLLAIVLGRLIQSFELLTPPGVKKIDLTET GGQFSLRIANHSTVVARPLAV	517	Phytozome: Pp3c4_21680, Phypa_149501<br>Nucleotide sequence in GenBank: ABEU02000004.1<br>UniProt: A0A2K1KPH8
Pp3c3_17840<br>**PpC4H3**	MGKPMGIGVLLVILVFGVVAKVDFKSRILLDFAFSSSLLMQALLLG VFIVIVVSLRREGRNMNLPPCPAAFPIVGNWLQVGNDLKHRKLA EMAQKYGDVFMLRMGRRRFVVVSSPEAAKEVLHTHGVEFASRT RNAVYDVFAGKGQDIVFTNYGDHWRRMRRIMTVPFVTHKVVKH AHFAWEDEVDHCIRDVEARSESATTGVVLRHRFQLMMYNIMYR MMFNSRFADEHDPLYLKLKGLNSERCRLPAQNSKYNYADFIPIL RPFLRGYLKVCQDVRDRRLTLFKERFVDERRKLVNAVGAAGAG EKCAIDHILEAQKKGEISENNVLYLIENINI**AAIESTL**LSIEWGVAEL VNHPEVQKRVQKELDEVLGDGHLVSEPDIHSGKLPFLTAVVKET LRLHMPIPLLVPHMNVKQAKIFGYDIPPESKILVNAWWIGNNPKF WDQPERFMPERFLSATSEATTVDFRFLPFGAGRRSCPGSAIAVP LLAIVLGRLVQKIDLLPPPGMTKVDVTESGGQFSLHMATHSTVVT RPRA	536	Phytozome: Pp3c3_17840, Phypa_150408<br>Nucleotide sequence in GenBank: ABEU02000003.1<br>UniProt: A0A2K1KUY0<br>No signal peptide or transmembrane part identified by DeepTMHMM

Name in this work	Amino acid sequence	Aa	Accession numbers and details
Pp3c13_14870	MKDRKMNLPPGPTALPIVGNWLKVGNDLKHRTLAEMSERYGDV<br>FMLKMGWRNYVVVSSPEAAKGVLHTQGEEFACRTRNAVFDIFA<br>GKGQDIVFTNYGDHWRRMRRILTVPFVTAKVLQHAHFAWEEEV<br>DEVMEDVQSRPESATAGVVLRHRFQLMMYNIVYRMMFNSRFAS<br>EYDPLYLKLKGLNGERCRLPAQNYKYNYADFFPFLKLFIRGYLKI<br>CQEVRDKRLSLFKEHFVDERRKLVNAVGVSGAEEKCAIDHMLEA<br>QKKGEISEDNILYLIENINVAAIESTLLSIEWGIAELVNHPNVQKRL<br>QAELLEVIGEGNLVAEPDTHNNKLPFLTAVVKETLRLHMPVPLLV<br>PLMNMRQAKLAGYDIPPQSKVLVNAWWIGNNSKFWDQPEKFM<br>PERFLPAAAENQSLDFRFLPFGAGRRSCPGTAIAMPLLAIVLGRL<br>LQKFDLLPPPGMSKVDVAESGGQFSLHMATHSIVVLRPRN	480	Phytozome:<br>Pp3c13_14870,<br>Phypa_87503,<br>Phypa_87504<br><br>Nucleotide sequence in GenBank: ABEU02000013.1<br><br>UniProt: A0A7I4F2K6<br><br>No signal peptide or transmembrane part identified by DeepTMHMM
Pp3c16_23740	MGLESAVQGLLVAVLVGLLIAKLRAPKLKLPPGTVALPIVGNWLQ<br>VGDDLNHRNLAEMSKKYGDVFMLKMGQRNLVMVSSPEAAKEV<br>LHTQGTEFGSRERNVVYDILIGNGQDMVFAVYEEDWRKMRRIM<br>TVPFFTSKVVQQSRGTWEDDALRVVEELRTRPEASTTGVVIRNR<br>LNSMMYNSIYRLMFDRRFENEEDPLFLRLKALNGGRSRLAQKGV<br>PGGPGPAPGGLQRTLRGRTQETVRDDGTKPDGEKAAIYLILEAQ<br>RKGEISEDNLLYIVENINVAAIETMLWSIEGGVAELVNNPDIQTRV<br>SEELDHTLGKGNLITELDTYNPKHWDQPGRFMPERFSGNNIEVS<br>GGDFRFLPFGSGRRSCRSIIIAMPLLTIVLSRLVQSMGQLSPPGM<br>KKVDVAEKGGQFSRHIATRSTMVCKPIA	426	Phytozome: Pp3c16_23740<br><br>Nucleotide sequence in GenBank: ABEU02000016.1<br><br>UniProt: A0A2K1J9T1<br><br>No signal peptide or transmembrane part identified by DeepTMHMM
Pp3c12_6560	MATAGTIMMQAVGMASMFTFQNVVQGLIVAMVVWIFVIKLKSPK<br>YKLPPGPVATGSRSDSSSHVGDDLNHRYLAELTKKYGDIFLLKM<br>GQRNVVVISSPEIAKDALQTQGIAFGSRPRNVVFDIFSGKGQDM<br>VFTPYGEHWRLMRRITTAPLFTNKVVQQSRLQLMMYNIIYRMMF<br>TSRFKDEKDPLYLKLKVLNGERSRLGQSFDYNYGDFIPILRPFLR<br>GYLKVCQDVKDRRIALFKEYFVDERRKLTSVNPPKSEEQKCAID<br>YIFEAEKLGEINEDNVLYIVENINGIAELVNNPELDGIREELDAVLG<br>KGNMVIEPDTYNNKLPLLTAFVKEVMRLHMSIPHVVHMNLKHEK<br>LAGYDIPAESKILVNDQPEKFMPERFLDGNIETSGNDFRYLPFGV<br>GRRRCPGIFIAMPLLSIVLGRLIQSFELLPPPGVKEVDVTEFCGQF<br>SLRIANHSTVVVRPLL	464	Phytozome: Pp3c12_6560<br><br>Nucleotide sequence in GenBank: ABEU02000012.1<br><br>UniProt: A0A2K1JPP5<br><br>No signal peptide or transmembrane part identified by DeepTMHMM
Helianthus tuberosus			
HtC4H1	MDLLLIEKTLVALFAAIIGAILISKLRGKKFKLPPGPIPVPIFGNWLQ<br>VGDDLNHRNLTDLAKRFGEILLLRMGQRNLVVVSSPELAKEVLH<br>TQGVEFGSRTRNVVFDIFTGKGQDMVFTVYGEHWRKMRRIMTV<br>PFFTNKVVQQYRYGWEAEAAAVVDDVKKNPAAATEGIVIRRRLQ<br>LMMYNNMFRIMFDRRFESEDDPLFLKLKALNGERSRLAQSFEYN<br>YGDFIPILRPFLRNYLKLCKEVKDKRIQLFKDYFVDERKKIGSTKK<br>MDNNQLKCAIDHILEAKEKGEINEDNVLYIVENINV**AAIETT**LWSIE<br>WGIAELVNHPEIQAKLRHELDTKLGPGVQITEPDVQNLPYLQAVV<br>KETLRLRMAIPLLVPHMNLHDAKLGGFDIPAESKILVNAWWLANN<br>PDQWKKPEEFRPERFLEEEAKVEANGNDFRYLPFGVGRRSCPG<br>IILALPILGITIGRLVQNFELLPPPGQSKIDTDEKGGQFSLHILKHSTI<br>VAKPRSF	505	Nucleotide sequence in GenBank: Z17369.1<br><br>UniProt: Q04468<br><br>CYP73A1
Sorghum bicolor			
SbC4H1	MDLVLLEKALLGLFAAAVLAVAVAKLTGKRYRLPPGPAGAPVVG<br>NWLQVGDDLNHRNLMSLAKRFGDIFLLRMGVRNLVVVSTPELAK<br>EVLHTQGVEFGSRTRNVVFDIFTGKGQDMVFTVYGDHWRKMR<br>RIMTVPFFTNKVVAQNRVGWEEEARLVVEDVRKDPRAAAEGVVI<br>RRRLQLMMYNDMFRIMFDTRFESEQDPLFNKLKALNAERSRLS<br>QSFEYNYGDFIPVLRPFLRGYLNRCHDLKTRRMKVFEDNFVQER<br>KKVMAQTGEIRCAMDHILEAERKGEINHDNVLYIVENINV**AAIETT**<br>LWSIEWGIAELVNHPAIQSKLREEMDSVLGAGVPVTEPDLERLPY<br>LQAIVKETLRLRMAIPLLVPHMNLNDGKLAGYDIPAESKILVNAWF<br>LANDPKRWVRPDEFRPERFLEEEKTVEAHGNDFRFVPFGVGRR<br>SCPGIILALPIIGITLGRLVQNFQLLPPPGQDKIDTTEKPGQFSNQI<br>AKHATIVCKPLEA	501	Nucleotide sequence in GenBank: AY034143.1<br><br>UniProt: Q94IP1<br><br>CYP73A33

Table S2 A Pairwise sequence comparison (EMBOSS Needle) of all retrieved C4H amino acid sequences from *Physcomitrium patens*. **B** Small phylogenetic tree depicting the relationship of these sequences

**A**	**Pp3c3_17840V3 PpC4H3**	**Pp3c4_21680V3 PpC4H2**	Pp3c13_14870V3	**Pp3c25_10190V3 PpC4H1**	Pp3c16_23740V3	Pp3c12_6560V3
**Pp3c3_17840V3 PpC4H3**	100	63.5/77.1	71.9/81.2	64.3/78.5	44.4/55.4	50.1/62.0
**Pp3c4_21680V3 PpC4H2**		100	64.6/77.7	76.7/86.2	54.4/64.6	68.3/76.3
Pp3c13_14870V3			100	59.7/73.3	46.5/58.3	51.5/62.8
**Pp3c25_10190V3 PpC4H1**				100	58.8/65.7	59.8/70.6
Pp3c16_23740V3					100	43.9/54.1
Pp3c12_6560V3						100

B

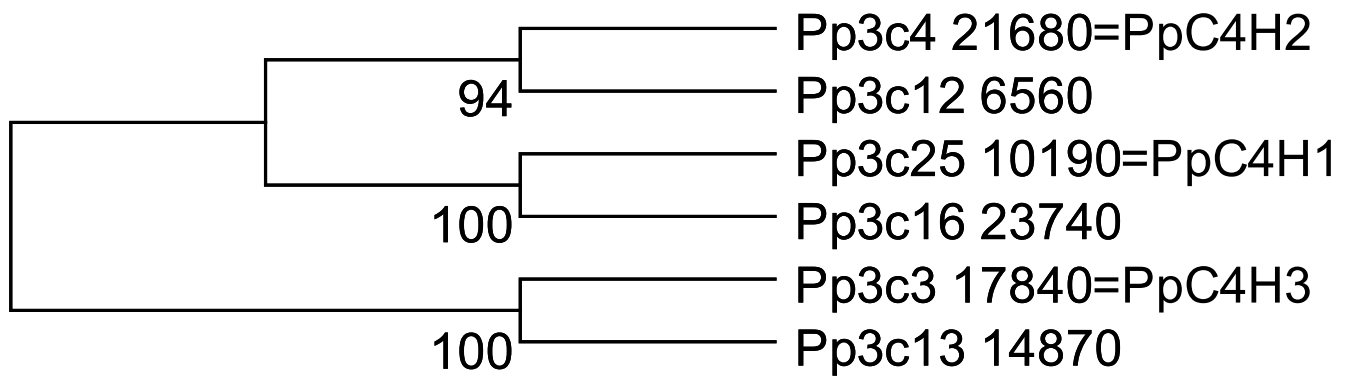

Table S3 A Pairwise sequence comparison (EMBOSS Needle) of all retrieved C4H amino acid sequences from *Marchantia polymorpha.* **B** Small phylogenetic tree depicting the relationship of these sequences

**A**	**0163s0018 MpC4H1**	0129s0029	0084s0023	0041s0103	0010s0184	0084s0010	**0081s0008 MpC4H2**	**0081s0009 MpC4H3**
**0163s0018 MpC4H1**	100	30.2/46.6	28.4/44.5	25.5/43.7	28.2/45.4	25.6/45.5	75.8/87.2	74.3/85.0
0129s0029		100	31.7/47.2	32.0/48.5	34.0/51.5	34.3/52.0	32.2/48.2	33.1/50.6
0084s0023			100	63.1/76.7	38.5/58.1	40.8/57.9	27.0/44.3	25.7/44.7
0041s0103				100	40.3/58.5	40.2/57.6	24.0/42.8	27.7/46.0
0010s0184					100	82.0/91.3	27.5/43.8	29.8/47.0
0084s0010						100	27.2/46.6	27.2/46.6
**0081s0008 MpC4H2**							100	81.0/89.2
**0081s0009 MpC4H3**								100

B

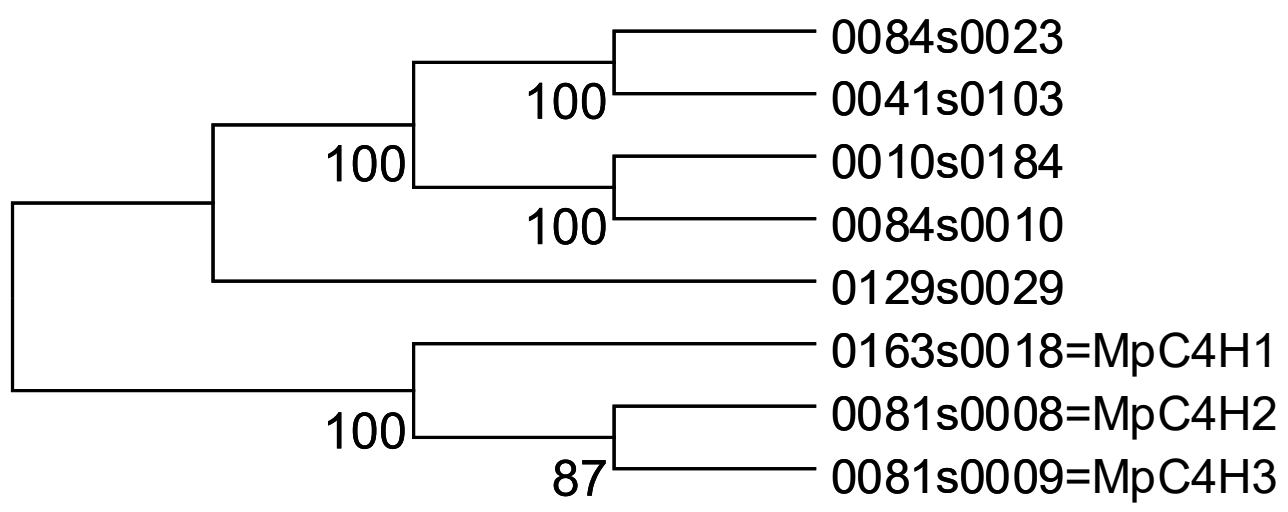

Table S4 CYP98 sequences characterized in this work with their amino acid sequences, lengths and accession numbers together with CYP98 from *Ocimum basilicum* (Gang *et al.*, 2002). Light grey shading indicates parts of the protein inside the endoplasmic reticulum (ER), signal peptides are shaded turquoise, while transmembrane stretches are shaded red as calculated by DeepTMHMM 1.0.57 on https://dtu.biolib.com. Unshaded parts of the protein are on the cytosolic side of the ER membrane. The signature motif for CYP98 **AGMDTT** or variants thereof are written in **bold**. Aa = number of amino acid residues

Name in this work	Amino acid sequence	Aa	Accession numbers and details
Anthoceros agrestis (Ernst *et al.*, 2022)			
AaCYP98	MAELVVRAVLALIAVAVAAGFLFYRARWSRYKLPPGPKPRLF<br>VGNLLDVTAVRFKCFEQWAKVYGPVMSVWMGATLNVVVSS<br>AEGAKEVLKDKDQSLAARARSRAAAKFSRDGQDLIWADYG<br>PHYVKVRKVCNVELFTPKRLEALRPIRQDEVSAMVHKIHRDA<br>PKGPVNPKKYLSAMAFNNITRLAFGKRFVDDEGKIDSQGVE<br>FKEVIGQGMKLGASLKISEHIPSIRWMFPLQNEEFAKHGARR<br>DTLTRTIMKEHAALRETLGPQSHFVDALLGLQKQYDLSETTII<br>GLLWDMVT**AGMDTV**AITVEWALAELIRNPEVQRKAQEELDA<br>VVGDETVLTEAHFSKLPYLTALTKESLRLHPPTPLMLPHKCT<br>EDVKIQGYDIPKGTVVHCNIYVIARDPAVWKEPLAFRPERFLE<br>QDVDIKGHDFRLLPFGAGRRVCPGAQLGLNMVQLMIGRLLH<br>QFSWSPPEGVRAEDISLEERSGIVTYMAKSLEAVATPRLPQA<br>LYARNV	505	1kP: TWUW_scaffold_2050274 (partial)<br>University of Zurich: AagrOXF_evm.model.utg00000 9l.515.1; AagrBONN_evm.model.Sc2ySw M_228.160.1<br>Nucleotide sequence in GenBank: MT119883.1<br>UniProt: A0A8K0ZJB3
Marchantia polymorpha			
MpCYP98	MAEPDLDEMPSRGVIDLAGLQVDTYYTVVVATAVVLLFILKA<br>VWPSKKLAPGPRPRPFVGNLYEVKPVRFRCFMEWAEKYGP<br>ILSFWMGTTLNTVVSSAALAKEVLKDQDQFLSGRPLTRAAAR<br>FSRNGQDLIWADYGPHYVKVRKLCTLELFTHKRLEALRSVR<br>EEEVGGMVNAIYNDCQTGAGTVDVKTYLSAVAFNNITRIVFG<br>KKFVNQDGEIDAQGKEFKAVIAEGMKLGASLKTSEHIKYLRW<br>MFPLQEEAFARNNARRDNLTKAIMQEFATGGGDRGSDADS<br>AGSSQHFVNALLNFQKQYDLSEVTVITLLWDMIT**AGMDTT**AI<br>TVEWAMAELIRNPDVQRKAQEELDNVVGYERVVTEGDFRD<br>LPYLQAVAKEALRLHPPTPLMLPHKATEDVKVGGFDIPKGST<br>VHVNAFAIARDPSTWKDPESFRPERFLEEDIDIKGTDFRVIPF<br>GAGRRVCPGAQLGLVLVQIMLGRLLHHFTWSPPEGMRPEDI<br>DLTERPGVVTFMLKPLQAVATPRLPSALYRTV	529	Phytozome: Mapoly0037s0087<br>Marpolbase: Mp_3g11100<br>Nucleotide sequence in GenBank: KZ772709.1<br>UniProt: A0A2R6X492, A0AAF6AZK8-1
Physcomitrium patens			
PpCYP98	MAVMWENTYTVAAIVAALLFMMYKSLRSSHKLPPGPRPLPV<br>VGNLTHITPVRFKCFMEWAQTYGSVLSVWMGPTLNVVVSS<br>ADAAKEMLKERDHALSSRPLTRAAARFSRNGQDLIWADYGP<br>HYVKVRKVCTLELFTFKRLESLKPVREDEVGAMVAALFKDC<br>ADSRPLNLKKYVSAMAFNNITRIVFGKRFVDDKGNIDNQGVE<br>FKEIVSQGMKLGASLKMSEHIPYLRWMFPLQEEEFAKHGAR<br>RDNLTKAIMQEHRLQSQKNGPGHHFVDALLSMQKQYDLSE<br>TTIIGLLWDMIT**AGMDTT**AISVEWAIAELVRNPDVQVKAQQEL<br>DQVVGQDRVVTEADFSQLPYLQAVAKEALRLHPPTPLMLPH<br>KATETVKIGGYDVPKGTVVHCNVYAISRDPTVWEEPLRFRP<br>ERFLEEDIDIKGHDYRLLPFGAGRRVCPGAQLGLNMVQLML<br>ARLLHHFSWAPPPGVTPAAIDMTERPGVVTFMAAPLQVLAT<br>PRLRAALYKNGSSPS	508	Phytozome: Pp3c22_19010, Phypa_98753<br>Nucleotide sequence in GenBank: ABEU02000022.1<br>UniProt: A0A2K1IP10

Name in this work	Amino acid sequence	Aa	Accession numbers and details
Ocimum basilicum			
ObCYP98	MAALLLLLLLPLLLPAIFLLHHLYYRLRFRLPPGPRPLPIVGNL<br>YDVKPVRFRCFADWAQSYGPIISVWFGSTLNVIVSNTELAKE<br>VLKEKDQQLADRHRSRSAAKFSRDGQDLIWADYGPHYVKV<br>RKVCTLELFSPKRLEALRPIREDEVTAMVESIYHDCTAPDNA<br>GKSLLVKKYLGAVAFNNITRLAFGKRFVNSEGIIDKQGLEFKA<br>IVSNGLKLGASLAMAEHIPSLRWMFPLDEDAFAKHGARRDQ<br>LTREIMEEHTRAREESGGAKQHFFDALLTLKDKYDLSEDTIIG<br>LLWDMIT**AGMDTT**AISVEWAMAELIKNPRVQQKAQEELDRVI<br>GYERVMTELDFSNLPYLQCVAKEALRLHPPTPLMLPHRSNS<br>NVKIGGYDIPKGSNVHVNVWAVARDPAVWKNPCEFRPERFL<br>EEDVDMKGHDFRLLPFGAGRRVCPGAQLGINLVTSMIGHLL<br>HHFNWAPPSGVSSDELDMGENPGLVTYMRTPLEAVPTPRL<br>PSDLYKRIAVDL	512	Nucleotide sequence in GenBank: AY082611.1<br>UniProt: Q8L5H8<br>No signal peptide or transmembrane part identified by DeepTMHMM
Mesotaenium kramstae			
scaffold-NBYP-2004150	LLLVGIATALLWRAFSSVLCSGRQFPPGPRPWPVVGNIPNLA<br>AGGPLPYQTLATLAQEYGPVMSVWLGSNLTVVVSSPEAARE<br>VLREFDQACQSRPLPESAKVLTYAGRDIGFAPAGDHWKLMR<br>KLCTTNLLTKRRLETFKPIRMEETSLMCGAVKEEAERRDGLV<br>NVRKHLFRTSLNNIMRIALGKRFEYSVPGRPNPEADEFFAYV<br>KDAFELIAAFNLADHVPALRLFDPAGVRRRCRLVRAYLQKFF<br>EDAIDAHQRQLDALHLAPSHGGSAPSHGSTSGSTAGEAVVV<br>PEDFCDALLTMEGEERLGRIETVGMLQDMLV**AGADTS**STAA<br>EWALLELLRHPEKLSRLQAEVDTVLGHKSGEASGKDTDVEA<br>QLNELPYLRAVIKETLRLHPVGPLLVPHQTIAPVMLGGGKYYV<br>PAGTMVQVNVWAITHDPAIWPKPHEFLPERFLEQDVDFKGT<br>DFRFLPFGSGRRMCPGMALGASMVQLMLAHLVHAFDWSAG<br>PAGLDNAEKFGLAVGPRKALEAIATPR	524	1kP database:<br>scaffold-NBYP-2004150<br>No signal peptide or transmembrane part identified by DeepTMHMM

Table S5 Pairwise sequence comparison (EMBOSS Needle) of all retrieved amino acid sequences for CYP98 in *Anthoceros agrestis, Marchantia polymorpha* and *Physcomitrium patens*

	AaCYP98	MpCYP98	PpCYP98
AaCYP98	100	64.0/76.8	67.7/80.7
MpCYP98		100	65.5/77.3
PpCYP98			100

Table S6 CYP73 and CYP98 amino acid sequences used to construct the phylogenetic tree (Fig. 1). CYP98 sequences are shaded in grey. Sequences characterized in this study are written in **bold**

Accession	Species	Abbreviation	Family
ADO24190.1	*Allium sativum*	Allsat	Amaryllidaceae
**QHF16161.1**	*Anthoceros agrestis*	Antagr	Anthocerotaceae
**TWUW_scaffold_2050391**			
QPI70542.1			
AGP25594.1	*Aquilaria sinensis*	Aqusin	Thymelaeaceae
NP_180607.1	*Arabidopsis thaliana*	Aratha	Brassicaceae
NP_850337.1			
XP_013629897.1	*Brassica oleracea var. oleracea*	Braole	

AAG43824.1	*Capsicum annuum*	Capann	Solanaceae
AAC35857.1	*Capsicum chinense*	Capchi	
KAL1220111.1	*Cardamine amara subsp. amara*	Carama	Brassicaceae
P48522.1	*Catharanthus roseus*	Carros	Apocynaceae
KAH7446516.1	*Ceratopteris richardii*	Cerric	Pteridaceae
**GBG60678.1**	*Chara braunii*	Chabra	Characeae
**GBG60692.1**			
O81928.2	*Cicer arietinum*	Cicari	Fabaceae
AAK57011.1	*Citrus x paradisi*	Citpar	Rutaceae
AAF66066.2	*Citrus sinensis*	Citsin	
ASA39650.1	*Conocephalum japonicum*	Conjap	Conocephalaceae
XP_057845640.1	*Cryptomeria japonica*	Cryjap	Cupressaceae
CAK95273.1	*Cucumis sativus*	Cucsat	Cucurbitaceae
AFX98061.1	*Cunninghamia lanceolata*	Cunlan	Cupressaceae
KAJ7568619.1	*Diphasiastrum complanatum*	Dipcom	Lycopodiaceae
AIS92509.1	*Epimedium sagittatum*	Episag	Berberidaceae
AAY54293.1	*Ginkgo biloba*	Ginbil	Ginkgoaceae
Q96423.1	*Glycyrrhiza echinata*	Glyech	Fabaceae
Q42797.1	*Glycine max*	Glymax	
NP_001235563.1			
AAG10197.1	*Gossypium arboreum*	Gosarb	Malvaceae
Q04468.1	*Helianthus tuberosus*	Heltub	Asteraceae
KAJ0262444.1	*Hirschfeldia incana*	Hirinc	Brassicaceae
XP_062077445.1	*Humulus lupulus*	Humlup	Cannabaceae
QWK52435.1	*Isatis tinctoria*	Isatin	Brassicaceae
kfl00035_0080_v1.1	*Klebsormidium nitens*	Klenit	Klebsormidiaceae
BAB71717.1	*Lithospermum erythrorhizon*	Litery	Boraginaceae
AWW24970.1	*Lycoris radiata*	Lycrad	Amaryllidaceae
ASA39653.1	*Marchantia emarginata*	Marema	Marchantiaceae
ASA39648.1	*Marchantia paleacea*	Marpal	
**PTQ28464.1**	*Marchantia polymorpha*	Marpol	
**PTQ34277.1**			
**PTQ34278.1**			
BFI27419.1			
BFI36307.1			
BFI33772.1			
BFI36872.1			
BFI36292.1			
**PTQ40913.1**			
P37114.1	*Medicago sativa*	Medsat	Fabaceae
AAD11427.1	*Mesembryanthemum crystallinum*	Mescry	Aizoaceae
AUG71936.1	*Narcissus pseudonarcissus*	Narpse	Amaryllidaceae
AAK62344.1	*Nicotiana tabacum*	Nictab	Solanaceae
AAL99200.1	*Ocimum basilicum*	Ocibas	Lamiaceae
ACA25599.1	*Parthenocissus henryana*	Parhen	Vitaceae
Q43033.1	*Petroselinum crispum*	Petcri	Apiaceae
O24315	*Phaseolus vulgaris*	Phavul	Fabaceae
**XP_024365375.1**	*Physcomitrium patens*	Phypat	Funariaceae
**XP_024373833.1**			
**XP_024369607.1**			
XP_024392294.1			
PNR43514.1			
PNR38288.1			
**XP_024360823.1**			
AAD23378.1	*Pinus taeda*	Pintae	Pinaceae
AAL47685.1			
Q43067.2	*Pisum sativum*	Pisat	Fabaceae

ASA39646.1	*Plagiochasma appendiculatum*	Plaapp	Aytoniaceae
Q43054.1	*Populus sieboldii x Populus grandidentata*	Popsie	Salicaceae
AFZ78542.1	*Populus tomentosa*	Poptom	Salicaceae
AAG50231.1	*Populus trichocarpa x Populus deltoides*	Poptri	
AHY94893.1	*Prunella vulgaris*	Pruvul	Lamiaceae
CAC35977.1	*Ruta graveolens*	Rutgra	Rutaceae
I32769.1	*Scutellaria baicalensis*	Scubai	Lamiaceae
QIC52990.1	*Solanum nigrum*	Solnig	Solanaceae
AAK54447.1	*Sorghum bicolor*	Sorbic	Poaceae
AAG17469.1	*Triticum aestivum*	Triaes	
P37115.1	*Vigna radiata var. radiata*	Vigrad	Fabaceae
Q43240.1	*Zinnia elegans*	Zinele	Asteraceae

Table S7 List of primers used for PCR and sequencing. Underlined are the base pairs binding to the inserted gene, restriction sites are marked in **bold letters**

**Name**	**Amino acid sequence (5'→ 3')**	**Restriction site**	**Comment**
Mutagenesis primer for CbCYP$_{cand}$1:K376R			
CbCYP$_{cand}$1:K376R_f	GGAAGGTGGTAGATCTGGTGACG		Mutation in red
CbCYP$_{cand}$1:K376R_r	CGTCACCAGATCTACCACCTTCC		Mutation in red
Homologous recombination for CbCYP$_{cand}$1			
CbCYP$_{cand}$1_pESC-Ura_HR_f	TTTTTGAAAATTC**GAATTC**ATGGG TGCTGATGTTGGTTCTTTTGCTG	EcoRI	Used for mutated variants as well
CbCYP$_{cand}$1_pESC-Ura_HR_r	TCGATACTAGT**GCGGCCGC**ACCC ATAGATCTTGG	NotI	Used for mutated variants as well
Homologous recombination for PpCYP98			
PpCYP98_pESC-Ura_HR_f	TTTTGAAAATTC**GAATTC**ATGGCT GTTATGTGGGAAAATACATATACA GTTGCTGC	EcoRI	
PpCYP98_pESC-Ura_HR_r	CGTCATCCTTGTAATCCATCGATA CTAGT**GCG**		
Primer for colony PCR of pESC-Ura MCS I and II			
pESC-Ura_MCS_I_f	CTGAAAGTTCCAAAGAGAAG		
pESC-Ura_MCS_I_r	TTTCTGGCAAGGTAGACAAG		
pESC-Ura_MCS_II_f	GCCTTATTTCTGGGGTAATTAATC AGCG		

pESC-Ura_MCS_II_r	GTCCCAAAACCTTCTCAAGCAAG<br>G		
Sequencing primer for pESC-Ura vector			
pESC-Ura_MCS_I_f	CTGAAAGTTCCAAAGAGAAG		
pESC-Ura_MCS_I_r	TTTCTGGCAAGGTAGACAAG		
pESC-Ura_MCS_II_f	GCCTTATTTCTGGGGTAATTAATC<br>AGCG		
pESC-Ura_MCS_II_r	GTCCCAAAACCTTCTCAAGCAAG<br>G		
$CbCYP_{cand}1$_mid	GAATTAATGACTATTCACATGG		

Table S8 PCR setups for each method. Standard protocols for each PCR were used according to the manufacturer. The primer concentration was adjusted before adding 0.5 µl of each primer to the reaction

Reaction	Polymerase	c (primer) [µM]	Mastermix	V [µl]
Mutagenesis	CloneAmp™ HiFi PCR Premix Takara Bio	10	Provided in kit	25
Homologous recombination	AccuPrime™ Taq DNA Polymerase invitrogen	10-100	Prepared according to manufacturer	25
Colony PCR for MCS I and MCS II	GoTaq® G2 DNA Polymerase Promega	10	Prepared according to manufacturer	25

Table S9 PCR programs for each method. Each protocol was preceded by an additional denaturation period at 95 °C for 2 minutes and an additional elongation period of 8 minutes after the last cycle at the same temperature. The mutagenesis PCR used the CloneAmp™ HiFi PCR Premix (Takara Bio). Amplification for homologous recombination were performed with the AccuPrime™ *Taq* DNA polymerase standard protocol (Invitrogen) and Colony PCR used the GoTaq® G2 DNA polymerase standard protocol (Promega)

Reaction	Denaturation	Annealing	Elongation	Cycles
Mutagenesis	95.0 °C for 0.5 min	63.0 °C for 0.5 min	72.0 °C for 3.0 min	30
Homologous recombination $CbCYP_{cand}1$	95.0 °C for 1.0 min<br>95.0 °C for 1.0 min	72.0 °C for 1.0 min<br>67.0 °C for 1.0 min	68.0 °C for 2.5 min<br>68.0 °C for 2.5 min	10<br>30
Homologous recombination	95.0 °C for 1.0 min	76.0 °C for 1.0 min<br>-1 °C per cycle	68.0 °C for 2.0 min	8

PpCYP98	95.0 °C for 1.0 min	68.0 °C for 1.0 min	68.0 °C for 2.0 min	40
Colony PCR MCS I	95.0 °C for 1.0 min	49.0 °C for 1.0 min	70.0 °C for 2.4 min	40
Colony PCR MCS II	95.0 °C for 1.0 min	59.4 °C for 1.0 min	70.0 °C for 2.4 min	40

Table S10 Table of solvents and detection wavelengths used to analyze each product by HPLC

Product	% MeOH	nm
4-coumaric acid	35	309
3-hydroxypropanoic acid	30	280
caffeic acid	30	325
N-(caffeoyl)-anthranilate	45	333
N-(caffeoyl)-3-hydroxyanthranilate	45	333
N-(caffeoyl)-tyramine	30	325
caffeoyl-5-*O*-shikimate	30	325
chlorogenate	20	333
caffeoyl-3-*O*-quinate	20	333
caffeoyl-4-hydroxyphenyllactate	45	333
rosmarinate	45	333
caffeoyl-4-*O*-threonate	30	333

References

Ernst, L., Wohl, J., Bauerbach, E. and Petersen, M. (2022) Hydroxycinnamoyltransferase and CYP98 in phenolic metabolism in the rosmarinic acid-producing hornwort *Anthoceros agrestis*. *Planta*, 255, 75. doi.org/10.1007/s00425-022-03856-9

Gang, D.R., Beuerle, T., Ullmann, P., Werck-Reichhart, D. and Pichersky, E. (2002) Differential production of *meta* hydroxylated phenylpropanoids in Sweet Basil peltate glandular trichomes and leaves is controlled by the activities of specific acyltransferases and hydroxylases. *Plant Physiol.*, 130, 1536–1544. doi.org/10.1104/pp.007146

Schoch, G.A., Attias, R., Le Ret, M. and Werck-Reichhart, D. (2003) Key substrate recognition residues in the active site of a plant cytochrome P450, CYP73A1. Homology guided site-directed mutagenesis. *Eur. J. Biochem.*, 270, 3684–3695. doi.org/10.1046/j.1432-1033.2003.03739.x

Zhang, B., Lewis, K.M., Abril, A., Davydov, D.R., Vermerris, W., Sattler, S.E. and Kang, C. (2020) Structure and Function of the Cytochrome P450 Monooxygenase Cinnamate 4-hydroxylase from *Sorghum bicolor*. *Plant Physiol.*, 183, 957. doi.org/10.1104/pp.20.00406
